# Standardization of a Developmental Milestone Scale Using Data From Children in Israel

**DOI:** 10.1001/jamanetworkopen.2022.2184

**Published:** 2022-03-14

**Authors:** Tamar Sudry, Deena R. Zimmerman, Hadar Yardeni, Adina Joseph, Ravit Baruch, Itamar Grotto, Dan Greenberg, Roni Eilenberg, Guy Amit, Pinchas Akiva, Meytal Avgil Tsadok, Yitzhak Rize, Hani Zaworbach, Moshe Uziel, Dror Ben Moshe, Irit Lior Sadaka, Eitan Bachmat, Judah Freedman, Yair Sadaka

**Affiliations:** 1 Neuro-Developmental Research Center, Mental Health Institute, Be’er-Sheva, Israel; 2 KI Research Institute, Kfar Malal, Israel; 3 Public Health Services, Israel Ministry of Health, Jerusalem, Israel; 4 Department of Child Development and Rehabilitation, Israel Ministry of Health, Jerusalem, Israel; 5 School of Public Health, Faculty of Health Sciences, Ben-Gurion University of the Negev, Be’er-Sheva, Israel; 6 Department of Health Policy and Management, School of Public Health, Faculty of Health Sciences, Ben-Gurion University of the Negev, Be’er-Sheva, Israel; 7 TIMNA Initiative, Big Data Platform, Israel Ministry of Health, Jerusalem, Israel; 8 Department of Computer Science, Ben-Gurion University of the Negev, Be’er-Sheva, Israel; 9 Faculty of Health Sciences, Ben-Gurion University of the Negev, Be’er-Sheva, Israel

## Abstract

**Importance:**

Routine developmental screening tests for children are used worldwide for early detection of developmental delays. However, assessment of developmental milestone norms lacks strong normative data, and there are inconsistencies among different screening tools.

**Objective:**

To establish milestone norms and build an updated developmental scale.

**Design, Setting, and Participants:**

This is a cross-sectional, population-based study conducted between 2014 and 2020. Developmental assessments were conducted by trained public health nurses, documented in national maternal child health clinics, known as *Tipat Halav*, which serve all children in Israel. Participants included all children born between January 2014 and September 2020, who were followed at the maternal child health clinics from birth to age 6 years. Exclusion criteria were preterm birth, missing gestational age, low birth weight (<2.5 kg), abnormal weight measurement (<3% according to standardized child growth charts), abnormal head circumference measurement (<3% or >97% according to standardized child growth charts), and visits without developmental data or without the child’s age. Data analysis was performed from September 2020 to June 2021.

**Exposures:**

In total, 59 milestones in 4 developmental domains were evaluated, and the achievement rate per child’s age was calculated for each milestone.

**Main Outcomes and Measures:**

A contemporary developmental scale, the Tipat Halav Israel Screening (THIS) Developmental Scale, was built, presenting the 75%, 90%, and 95% achievement rates for each milestone. The THIS scale was compared with other commonly used screening tests, including the Denver Developmental Screening Test II (Denver II), the Alberta Infant Motor Scale (AIMS), and the Centers for Disease Control and Prevention (CDC) Developmental Assessment.

**Results:**

A total of 839 574 children were followed in the maternal child health clinics between January 2014 and September 2020 in Israel, and 195 616 children were excluded. A total of 3 774 517 developmental assessments were performed for the remaining 643 958 children aged 0 to 6 years (319 562 female children [49.6%]), resulting in the establishment of new developmental norms. In terms of the comparable milestones, THIS milestones had a match of 18 of 27 (67%) with the Denver II, 7 of 7 (100%) with AIMS, and 10 of 19 (53%) with the CDC Developmental Assessment. The remaining unmatched milestones were achieved earlier in the THIS scale compared with other screening tools.

**Conclusions and Relevance:**

The THIS developmental scale is based on the largest population evaluated to date for developmental performance, representing the heterogeneous, multicultural population comprising this cohort. It is recommended for further evaluation worldwide.

## Key Points

**Question:**

Can accurate developmental norms for children be determined on the basis of national developmental assessments of a multicultural population?

**Findings:**

This cross-sectional, population-based study analyzed 3 774 517 developmental assessments of 643 958 children from birth to age 6 years, conducted by trained nurses in Maternal Child Health Clinics (known as *Tipat Halav*) in Israel. A contemporary developmental scale, the Tipat Halav Israel Screening Developmental Scale, was built accordingly, presenting the 75%, 90%, and 95% achievement rate for evaluated milestones.

**Meaning:**

This developmental scale based on a diverse population in Israel is recommended for further evaluation worldwide.

## Teaser

This cross-sectional study aims to establish milestone norms and build an updated developmental scale for children from birth to age 6 years.

## Introduction

According to recent statistics, in the US as many as 1 in 4 children from birth to age 5 years are at moderate or high risk for developmental, behavioral, or social delay.^1^ Early detection of delay is crucial for determining which children require close surveillance and intervention.^2,3,4,5^ Early intervention has been proven to be critical in optimizing language, cognitive, motor, socioemotional development, and educational success, alongside prevention of additional future developmental delay.^6,7,8,9,10,11,12,13^

Because of the importance of early intervention, international health organizations recommend conducting regular developmental surveillance and screening for all children.^1,3,14,15,16^ Tools for assessing child development are of central importance in allocating resources and making decisions for the benefit of children with or at risk of developmental delay. Therefore, it is important that the developmental screening tools will most accurately reflect the natural distribution of developmental stage timing.

Worldwide, there are multiple developmental screening tools in use.^3^ However, assessment of developmental milestones lacks strong normative data,^17,18,19^ and there are inconsistencies among different screening tools regarding normative attainment age of commonly evaluated milestones.^18,20,21,22^ On the basis of the importance of milestone norms in any developmental screening tool, the current study aimed to establish milestones of normative timing and build a developmental scale accordingly. The new scale is based on developmental assessments of children conducted by trained public health nurses in a nationwide program. The establishment of normal attainment age of each milestone and the developmental scale built by this process in a multicultural country, with a diverse population of multiple ethnicities and languages, may further strengthen other developmental screening or diagnostic tools.

## Methods

All methods were performed in accordance with relevant guidelines and regulations. The study protocol was approved by the Soroka Medical Center institutional review board and was conducted in accordance to the principles of the Declaration of Helsinki.^23^ An exemption of informed consent was granted by this ethics committee because the data were anonymous. This cross-sectional study follows the Strengthening the Reporting of Observational Studies in Epidemiology (STROBE) reporting guidelines for cross-sectional studies.

### Maternal Child Health Clinics

In Israel, universal, free-of-charge pediatric preventive care for children from birth to age 6 years is provided in maternal child health clinics (MCHCs) known as *Tipat Halav* (“drop of milk”).^20^ The objectives of the services provided are prevention of infectious diseases by means of immunization, early detection of health problems by conducting routine growth, physical, and development examinations, and health education. Parents are instructed to visit the MCHC after hospital discharge and then at ages 1, 2, 4, 6, 9, 12, 18, 24, 36, 48, and 60 months. Developmental surveillance is conducted at all of the scheduled routine visits.

There are approximately 1000 MCHCs distributed throughout the country, managed by the Ministry of Health (MoH), 2 municipalities (Tel Aviv and Jerusalem), or 1 of the 4 Israeli health maintenance organizations. All clinics work according to the same national care protocols.^24^ This study is based on the database of all visits to the clinics run by the MoH, the municipalities, and 1 health maintenance organization (Leumit), all of which use the same electronic medical record. This group of children represent approximately 70% of the nation’s children in the age group of birth to 6 years.

### Developmental Screening and Documentation

One of the standardized protocols of MCHC refers to age-based developmental screening. The current protocol^25^ uses developmental milestones in 4 domains: gross motor, fine motor, language, and personal-social. The protocol has adapted milestones from other scales, including the Sally Provence Developmental Profile,^26^ Denver Developmental Scale,^17,27^ and Gesell Developmental Schedule,^28^ and was developed by an MoH task force consisting of pediatricians, nurses, physical therapists, speech pathologists, occupational therapists, and child psychologists. Description of these milestones can be found in eTable 1 in the Supplement.

Developmental assessment is performed by the MCHC nursing staff at each routine visit from birth to 6 years. The MCHC nursing staff includes certified public health nurses who are qualified to assess the child’s health, growth, and development. Each nurse has been trained specifically to conduct the developmental assessment protocol, and their training is evaluated and monitored at the beginning of their clinical practice. At each visit, a predefined group of age-related milestones is evaluated, according to the expected development at that age, as described in eTable 1 in the Supplement. However, some of the children arrive to the clinics for developmental assessment at a later age than expected. In these cases, previous milestones that were not yet evaluated are examined during the later visit.

The child’s performance of each milestone is reported as observed in the clinic. In cases of objective difficulty in appropriate evaluation, milestone achievements are documented according to the parent’s report. If the evaluated milestone was not achieved by observation or parental report, it is documented as unachieved. All results are documented within an electronic medical record (EMR).

### Electronic Medical Records

The clinical and demographic data collected by the clinic’s staff (nurses and physicians) are entered into an EMR system, specially developed for the MCHC. The demographic data contain parent-reported ethnicity, which is included in this study to emphasize the multicultural and multilingual nature of this database. The EMR system *Machshava Briah* (“Healthy Thought”)^24^ is used at the MCHCs. An anonymized version of the EMR database, with data from 2014 to 2020, was constructed by the Israel MoH Big Data Department for the current study.

### Tipat Halav Israel Screening Developmental Scale Study Cohort

The current study included all children born between January 1, 2014, and September 1, 2020, who were followed at the MCHCs and who had at least 1 developmental evaluation recorded during the study period. To assess the developmental norms for a healthy, low-risk population, exclusion criteria were defined to identify children with potential of abnormal development. The exclusion criteria included preterm birth (gestational age of <37 weeks), low birth weight (<2.5 kg), abnormal weight measurement (<3% according to standardized child growth charts), abnormal head circumference measurement (microcephaly [<3%] or macrocephaly [>97%] according to standardized child growth charts), missing gestational age at delivery, and visits without developmental data or without the child’s age.

### Statistical Analysis

**Figure 1.**
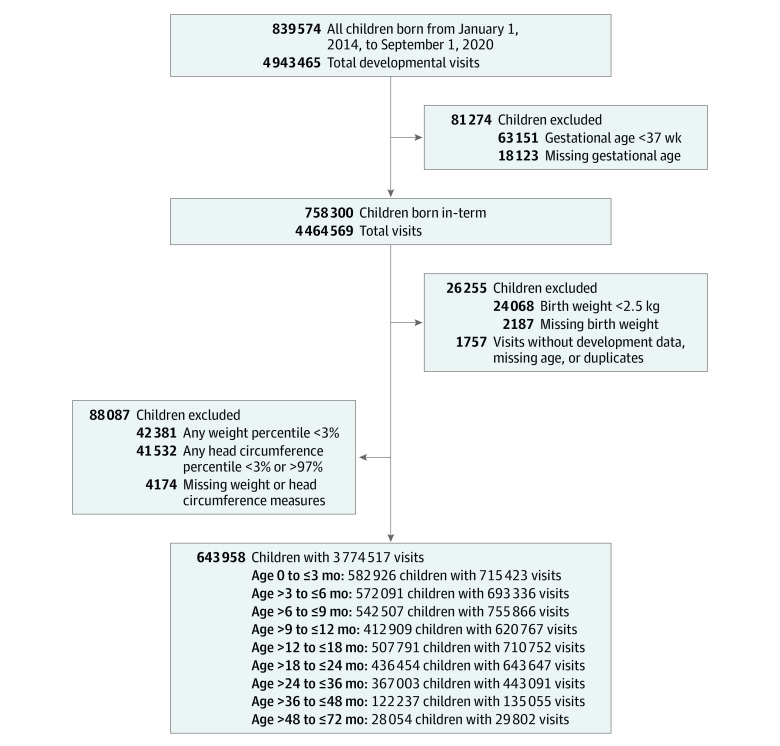
Participant Enrollment Flowchart Overview of the study population, including the exclusion criteria and total developmental assessments at each age group.

There were 59 developmental milestones, grouped into 9 age groups, as shown in Figure 1. For each developmental milestone, the first evaluation of the milestone for each child was extracted. Repeated evaluations that may have been performed for the same milestone were excluded to analyze a population undergoing initial screening and, thus, to avoid a potential bias toward children with developmental delays. For example, if a child aged 6 to 9 months had failed to crawl in the first evaluation and then succeeded at the following visit between the ages of 9 to 12 months, only the first failed evaluation was included in data analysis. Because the exact timing of the evaluation visits varied, we grouped children by their age on the date of the milestone evaluation and calculated the achievement rate for each age range. The time ranges that were used for grouping the children were 1 week for young age milestones (birth to 2 years) and 1 month for the older age milestones (2-6 years). Children were considered as achievers of the milestone if they were either observed in the clinic or reported by their parent to have accomplished the milestone. To determine success rates of 75%, 90%, and 95% of the children for each milestone, the achievement rate was calculated by dividing the number of achieving children by the total number of children examined at each age group, resulting in a series of success rates per age group. The final curve of a milestone’s achievement rate by age (eFigure 1, eFigure 2, eFigure 3, eFigure 4, eFigure 5, eFigure 6, eFigure 7, eFigure 8, and eFigure 9 in the Supplement) was obtained from this series by averaging adjacent values using a window of size 5 weeks or 3 months for the early age and older age, respectively. We applied linear interpolation to increase the temporal resolution of the trends and calculated the ages at which the success rate surpassed 75%, 90%, and 95% for the first time. To assess potential biases caused by the timing of the screening, we divided the visit time into quartiles and examined, within each age, the differences between the children with early and late screening time.

The Tipat Halav Israel Screening (THIS) Developmental Scale was compared with milestone attainment age of 3 other commonly used developmental tests: the Denver Developmental Screening Test II (Denver II), the Alberta Infant Motor Scale (AIMS), and the Centers for Disease Control and Prevention (CDC) Developmental Assessment.^15,17,29^ Because the exact definition of the evaluated milestones varies among these scales, we compared specific milestones that bear enough resemblance among the screening tests. For the THIS developmental scale, Denver II, and AIMS, failing to achieve a milestone was defined as not achieving the milestone at an age in which a 90% success rate was accomplished. For the CDC scale, it was defined as the age at which failure to achieve a milestone requires further medical evaluation. A meaningful clinical difference in milestone achievement among screening scales was defined as more than 1 month within the first 6 months of the child’s life, more than 2 months between ages 6 and 12 months, more than 3 months within the second and third year of life, and more than 6 months at age 3 to 6 years.

 Data analysis was performed from September 2020 to June 2021. Data analysis was performed using Python programming language version 3.6 (Python Software Foundation).

## Results

The electronic medical records of 839 574 children who visited the study MCHCs between January 2014 and September 2020 were available for analysis. Preterm children (gestational age <37 weeks; 63 151 children), infants with low birth weight (<2.5 kg; 24 068 infants) or missing birth weight (2187 infants), children with abnormal weight measurement (<3% according to standardized child growth charts; 42 381 children), abnormal head circumference measurement (<3% or >97% according to standardized child growth charts; 41 532 children), and children with missing gestational age (18 123 children) or missing weight or head circumference measures (4174 children) were excluded from the analysis. Visits without developmental data or with missing age were removed (1757 children). Thus, 195 616 children were excluded. A total of 643 958 children (319 562 female children [49.6%]) with 3 774 517 developmental evaluations were included in the analysis (Figure 1). The mean (SD) number of developmental evaluation visits per child was 5.9 (2.4) visits.

To assess a potential selection bias, the study cohort was compared with the Israeli population documented by the Israeli Central Bureau of Statistics.^30^ The study cohort represents approximately 70% of the children born in Israel within the study period, without meaningful changes throughout the years 2014 to 2019. The distributions of child’s sex and mother’s age in the cohort were similar to the national statistics, whereas the subpopulation of Arab children was slightly overrepresented compared with the Jewish population (76%-80% of the Israeli-Arab population vs 59%-68% of the Israeli-Jewish population).

The main demographic, birth, and maternal characteristics for each age group evaluated are summarized in the Table. The child population was composed of a nearly equal distribution of male (324 396 children [50.4%]) and female (319 562 children [49.6%]) children. The study cohort demonstrates the diverse population attending the MCHCs in Israel, with multiple ethnic groups, including Jewish, Arab, Druze, and others. Most mothers were Israel-born (528 861 mothers [82.1%]) and married (566 747 mothers [88.0%]), whereas maternal education varied, with less than one-third of the population having an academic degree (195 799 mothers [30.4%]) and approximately one-quarter having a high school education (171 411 mothers [26.6%]).

**Table.** Study Population Characteristics by Age Group

Cohort characteristics	Participants, No. (%)									
	Total (N = 643 958)	0 to ≤3 mo (n = 582 926)	>3 to ≤6 mo (n = 572 091)	>6 to ≤9 mo (n = 542 507)	>9 to ≤12 mo (n = 412 909)	>12 to ≤18 mo (n = 507 791)	>18 to ≤24 mo (n = 436 454)	>2 to ≤3 y (n = 367 003)	>3 to ≤4 y (n = 122 237)	>4 to ≤6 y (n = 28 054)
Child sex										
Female	319 562 (49.6)	289 559 (49.7)	284 256 (49.7)	270 017 (49.8)	205 273 (49.7)	252 684 (49.8)	217 267 (49.8)	182 435 (49.7)	61 344 (50.2)	13 930 (49.7)
Male	324 396 (50.4)	293 367 (50.3)	287 835 (50.3)	272 490 (50.2)	207 636 (50.3)	255 107 (50.2)	219 187 (50.2)	184 568 (50.3)	60 893 (49.8)	14 124 (50.3)
Ethnic group										
Arab	140 525 (21.8)	133 418 (22.9)	130 810 (22.9)	125 257 (23.1)	102 498 (24.8)	117 197 (23.1)	103 101 (23.6)	88 740 (24.2)	34 949 (28.6)	7082 (25.2)
Druse	11 796 (1.8)	11 475 (2.0)	11 206 (2.0)	10 890 (2.0)	9423 (2.3)	10 176 (2.0)	9233 (2.1)	7971 (2.2)	4319 (3.5)	538 (1.9)
Jewish	392 634 (61.0)	349 441 (59.9)	342 307 (59.8)	322 634 (59.5)	236 543 (57.3)	300 667 (59.2)	254 959 (58.4)	211 728 (57.7)	64 854 (53.1)	16 360 (58.3)
Missing	76 333 (11.9)	67 281 (11.5)	66 715 (11.7)	63 333 (11.7)	47 142 (11.4)	60 846 (12.0)	52 313 (12.0)	44 317 (12.1)	11 836 (9.7)	2921 (10.4)
Other^a^	22 670 (3.5)	21 311 (3.7)	21 053 (3.7)	20 393 (3.8)	17 303 (4.2)	18 905 (3.7)	16 848 (3.9)	14 247 (3.9)	6279 (5.1)	1153 (4.1)
Mother’s birth country										
Israel	528 861 (82.1)	479 985 (82.3)	470 756 (82.3)	446 192 (82.2)	338 327 (81.9)	418 073 (82.3)	360 165 (82.5)	304 631 (83.0)	103 045 (84.3)	23 264 (82.9)
Other^b^	58 983 (9.2)	51 603 (8.9)	50 793 (8.9)	47 829 (8.8)	35 485 (8.6)	45 024 (8.9)	37 766 (8.7)	30 250 (8.2)	7920 (6.5)	1983 (7.1)
Europe	25 180 (3.9)	22 462 (3.9)	22 123 (3.9)	21 066 (3.9)	16 110 (3.9)	19 346 (3.8)	16 324 (3.7)	13 946 (3.8)	4756 (3.9)	1213 (4.3)
Former Soviet Union	22 240 (3.5)	20 748 (3.6)	20 510 (3.6)	19 846 (3.7)	16 885 (4.1)	18 347 (3.6)	16 139 (3.7)	13 241 (3.6)	4645 (3.8)	1101 (3.9)
Ethiopia	8644 (1.3)	8081 (1.4)	7866 (1.4)	7535 (1.4)	6069 (1.5)	6964 (1.4)	6027 (1.4)	4905 (1.3)	1861 (1.5)	492 (1.8)
Missing	50 (<0.1)	47 (<0.1)	43 (<0.1)	39 (<0.1)	33 (<0.1)	37 (<0.1)	33 (<0.1)	30 (<0.1)	10 (<0.1)	<5 (<0.1)
Employment status										
Missing	199 544 (31.0)	178 338 (30.6)	176 036 (30.8)	167 653 (30.9)	126 114 (30.5)	159 720 (31.5)	138 090 (31.6)	118 748 (32.4)	38 743 (31.7)	9322 (33.2)
Not working	131 703 (20.5)	119 167 (20.4)	117 947 (20.6)	112 276 (20.7)	88 922 (21.5)	106 306 (20.9)	92 686 (21.2)	78 824 (21.5)	28 558 (23.4)	6265 (22.3)
Student	28 514 (4.4)	26 065 (4.5)	25 427 (4.4)	24 008 (4.4)	18 101 (4.4)	22 249 (4.4)	19 050 (4.4)	15 948 (4.3)	5423 (4.4)	1146 (4.1)
Working	284 197 (44.1)	259 356 (44.5)	252 681 (44.2)	238 570 (44.0)	179 772 (43.5)	219 516 (43.2)	186 628 (42.8)	153 483 (41.8)	49 513 (40.5)	11 321 (40.4)
Mother’s education level										
Academic degree	195 799 (30.4)	180 889 (31.0)	178 087 (31.1)	170 057 (31.3)	136 364 (33.0)	158 102 (31.1)	137 449 (31.5)	115 961 (31.6)	42 643 (34.9)	9083 (32.4)
Elementary school	13 442 (2.1)	12 208 (2.1)	12 046 (2.1)	11 300 (2.1)	9152 (2.2)	10 732 (2.1)	9235 (2.1)	7784 (2.1)	2493 (2.0)	688 (2.5)
High school	171 411 (26.6)	158 223 (27.1)	155 430 (27.2)	148 418 (27.4)	116 353 (28.2)	139 077 (27.4)	121 002 (27.7)	102 192 (27.8)	37 027 (30.3)	8272 (29.5)
Missing	199 021 (30.9)	174 040 (29.9)	171 094 (29.9)	160 972 (29.7)	114 971 (27.8)	152 122 (30.0)	128 663 (29.5)	108 483 (29.6)	30 739 (25.1)	7835 (27.9)
Tertiary education	64 285 (10.0)	57 566 (9.9)	55 434 (9.7)	51 760 (9.5)	36 069 (8.7)	47 758 (9.4)	40 105 (9.2)	32 583 (8.9)	9335 (7.6)	2176 (7.8)
Mother’s family status										
Married	566 747 (88.0)	513 990 (88.2)	504 205 (88.1)	478 649 (88.2)	364 300 (88.2)	448 556 (88.3)	386 976 (88.7)	326 829 (89.1)	110 505 (90.4)	25 175 (89.7)
Missing	43 380 (6.7)	37 999 (6.5)	37 191 (6.5)	34 593 (6.4)	24 872 (6.0)	32 096 (6.3)	26 217 (6.0)	20 914 (5.7)	5286 (4.3)	1291 (4.6)
Unmarried	20 005 (3.1)	18 354 (3.1)	18 174 (3.2)	17 244 (3.2)	13 975 (3.4)	15 814 (3.1)	13 428 (3.1)	10 931 (3.0)	3494 (2.9)	855 (3.0)
Other	7160 (1.1)	6449 (1.1)	6430 (1.1)	6147 (1.1)	4985 (1.2)	5761 (1.1)	4940 (1.1)	4112 (1.1)	1370 (1.1)	319 (1.1)
Divorced	6343 (1.0)	5828 (1.0)	5795 (1.0)	5576 (1.0)	4544 (1.1)	5296 (1.0)	4656 (1.1)	4005 (1.1)	1505 (1.2)	391 (1.4)
Widowed	323 (0.1)	306 (0.1)	296 (0.1)	298 (0.1)	233 (0.1)	268 (0.1)	237 (0.1)	212 (0.1)	77 (0.1)	23 (0.1)
Consanguinity										
No	606 227 (94.1)	547 578 (93.9)	537 525 (94.0)	509 570 (93.9)	386 126 (93.5)	476 956 (93.9)	409 582 (93.8)	344 240 (93.8)	114 342 (93.5)	26 439 (94.2)
Yes	37 731 (5.9)	35 348 (6.1)	34 566 (6.0)	32 937 (6.1)	26 783 (6.5)	30 835 (6.1)	26 872 (6.2)	22 763 (6.2)	7895 (6.5)	1615 (5.8)
Pregnancy week, median (IQR)	39.5 (38.6-40.3)	39.5 (38.6-40.3)	39.5 (38.6-40.3)	39.5 (38.6-40.3)	39.5 (38.6-40.3)	39.5 (38.6-40.3)	39.5 (38.6-40.3)	39.5 (38.6-40.3)	39.5 (38.6-40.2)	39.5 (38.6-40.1)
Birth weight, mean (SD), kg	3.3 (0.4)	3.3 (0.4)	3.3 (0.4)	3.3 (0.4)	3.3 (0.4)	3.3 (0.4)	3.3 (0.4)	3.3 (0.4)	3.3 (0.4)	3.3 (0.4)
Apgar score 1 min										
<8	18 862 (2.9)	17 220 (3.0)	16 945 (3.0)	16 123 (3.0)	12 848 (3.1)	14 980 (3.0)	13 042 (3.0)	11 070 (3.0)	3643 (3.0)	973 (3.5)
≥8	613 747 (95.3)	557 743 (95.7)	547 015 (95.6)	518 649 (95.6)	394 298 (95.5)	485 059 (95.5)	416 868 (95.5)	350 700 (95.6)	117 018 (95.7)	26 714 (95.2)
Missing	11 349 (1.8)	7963 (1.4)	8131 (1.4)	7735 (1.4)	5763 (1.4)	7752 (1.5)	6544 (1.5)	5233 (1.4)	1576 (1.3)	367 (1.3)
Apgar score 5 min										
<8	4112 (0.6)	3696 (0.6)	3655 (0.6)	3465 (0.6)	2863 (0.7)	3304 (0.7)	2910 (0.7)	2616 (0.7)	872 (0.7)	307 (1.1)
≥8	624 688 (97.0)	567 884 (97.4)	556 979 (97.4)	528 208 (97.4)	402 143 (97.4)	493 971 (97.3)	424 724 (97.3)	357 280 (97.4)	119 260 (97.6)	27 280 (97.2)
Missing	15 158 (2.4)	11 346 (1.9)	11 457 (2.0)	10 834 (2.0)	7903 (1.9)	10 516 (2.1)	8820 (2.0)	7107 (1.9)	2105 (1.7)	467 (1.7)
Head circumference, mean (SD), cm	34.5 (1.3)	34.5 (1.3)	34.5 (1.3)	34.5 (1.3)	34.5 (1.3)	34.5 (1.3)	34.5 (1.3)	34.5 (1.3)	34.5 (1.4)	34.5 (1.4)
Type of birth										
Cesarean delivery	92 540 (15.1)	85 191 (15.4)	84 181 (15.5)	80 263 (15.6)	62 285 (16.2)	73 949 (15.4)	63 792 (15.6)	53 317 (15.7)	18 152 (16.6)	3455 (16.3)
Instrumental	34 008 (5.6)	31 221 (5.6)	30 690 (5.7)	29 262 (5.7)	22 619 (5.9)	27 031 (5.6)	23 299 (5.7)	19 239 (5.7)	6782 (6.2)	1249 (5.9)
Spontaneous	485 464 (79.3)	436 337 (78.9)	427 590 (78.8)	404 393 (78.7)	299 370 (77.9)	377 874 (78.9)	321 920 (78.7)	267 567 (78.7)	84 259 (77.2)	16 549 (77.9)
Newborn position										
Breech	14 532 (2.3)	13 392 (2.3)	13 299 (2.3)	12 732 (2.3)	9931 (2.4)	11 815 (2.3)	10 227 (2.3)	8655 (2.4)	2909 (2.4)	543 (1.9)
Head	540 304 (83.9)	489 661 (84.0)	479 778 (83.9)	454 322 (83.7)	339 875 (82.3)	422 414 (83.2)	360 035 (82.5)	297 751 (81.1)	94 340 (77.2)	17 526 (62.5)
Missing	82 832 (12.9)	74 287 (12.7)	73 467 (12.8)	70 333 (13.0)	59 464 (14.4)	68 902 (13.6)	62 267 (14.3)	57 423 (15.6)	24 088 (19.7)	9836 (35.1)
Other	6290 (1.0)	5586 (1.0)	5547 (1.0)	5120 (0.9)	3639 (0.9)	4660 (0.9)	3925 (0.9)	3174 (0.9)	900 (0.7)	149 (0.5)
Mother’s age, y										
≤20	7101 (1.1)	6372 (1.1)	6469 (1.1)	6239 (1.2)	4900 (1.2)	6125 (1.2)	5764 (1.3)	5560 (1.5)	1919 (1.6)	232 (0.8)
>20 to ≤40	584 833 (90.8)	530 281 (91.0)	520 093 (90.9)	493 521 (91.0)	376 257 (91.1)	462 003 (91.0)	398 357 (91.3)	336 816 (91.8)	114 187 (93.4)	26 109 (93.1)
>40	45 095 (7.0)	40 517 (7.0)	40 153 (7.0)	37 956 (7.0)	29 207 (7.1)	35 585 (7.0)	29 572 (6.8)	23 022 (6.3)	5934 (4.9)	1667 (5.9)
Missing	6929 (1.1)	5756 (1.0)	5376 (0.9)	4791 (0.9)	2545 (0.6)	4078 (0.8)	2761 (0.6)	1605 (0.4)	197 (0.2)	46 (0.2)

^a^ Refers to Circassians, other Christians (non-Arab), and unknown.

^b^ Refers to North America, South and Central America, Asia, Africa, Australia, and Oceania.

### Comparison of Age Groups

The number of children examined at each of the 9 groups of age-related milestones ranged from 582 926 children at 0- to 3-month milestones to 28 054 children at 4- to 6-year milestones. The 9 age groups were defined according to the recommended time for evaluation of the milestones. Because the visit time varied and children who were not evaluated during the recommended time frame were evaluated later, during their actual visit, we performed a sensitivity analysis in which within each group of milestones we examined potential differences between the children with early and late screening time. As presented in eTable 2, eTable 3, eTable 4, eTable 5, eTable 6, eTable 7, eTable 8, eTable 9, and eTable 10 in the Supplement, some minor differences were observed; however, these differences do not appear to have clear clinical importance.

High parental compliance with MCHC visits was observed at early ages when visits include vaccine administration, as described in the Table. A comparison between the populations examined at age 0 to 3 years and 3 to 6 years, presented in eTable 11 in the Supplement, showed some minor differences between these groups. The main difference was the lower percentage of Jewish children in the older age group (72 830 children [54.2%] in the older age group vs 392 621 children [61.0%] in the younger age group). This is consistent with the greater overall compliance of the Jewish population in Israel with preventative health programs, which is also evidenced by their routine immunization coverage rates.^31^ In addition, the mother’s education level was lower in the older age group.

### Establishment of Milestone Norms and Building the THIS Developmental Scale

The curves of success rate by age for each milestone are presented in eFigure 1, eFigure 2, eFigure 3, eFigure 4, eFigure 5, eFigure 6, eFigure 7, eFigure 8, and eFigure 9 in the Supplement. For 18 of the 59 milestones evaluated, the achievement rate already exceeded 95% at the earliest screening time. Thus, the 95% achievement rate might have been reached at an earlier age. For 3 of the milestones, success rates did not exceed 95%; therefore, they were evaluated up to an achievement rate of 90% to 95%.

**Figure 2.**
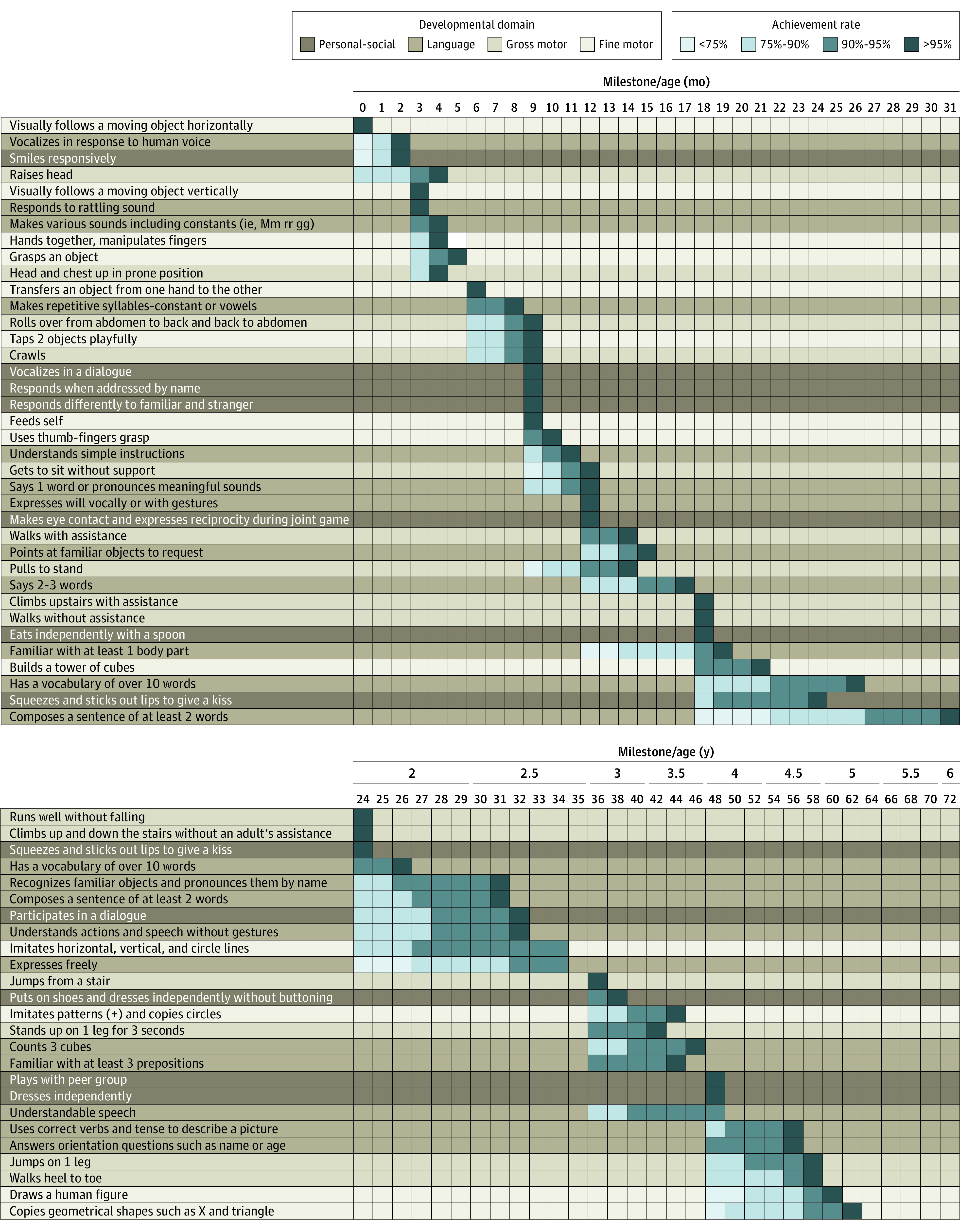
Tipat Halav Israel Screening Developmental Scale The rows represent the evaluated milestones, the columns represent the age by month (top panel) and year (bottom panel). Each milestone is colored according to the relevant field, as shown in the key (fine motor, gross motor, personal-social, and language). For each milestone the success rate is shown, each indicating a success threshold of less than 75% to 90%, 90% to 95%, and greater than 95%. For example, the milestone “vocalizes in response to human voice” is achieved by less than 75% of children at the age of 0 months, 75% to 90% of children at the age of 1 month, and more than 95% of children at the age of 2 months.

Because achievement of a milestone was defined as either clinical observation or parental report (in cases where observation was impossible), we compared this definition with the alternative of defining achievement only by clinical observation. The overall percentage of milestone achievement reported by parents (of all achieved milestones) was 8.7%. Per milestone, the mean (SD) percentage was 9.3% (10.3%) (median [IQR], 5.7% [2.3%-11.55%]). As shown in eFigure 10 in the Supplement, there is good overall agreement between the 2 percentages. Differences were observed in individual tasks related to language expression assessment and daily functions, such as getting dressed independently, which are difficult to assess in the clinical setting. The thresholds of 95% milestone achievement when including the parental report preceded those obtained using clinical observation alone by a mean (SD) of 2.0 (4.4) weeks (median [IQR] difference, 0.6 [0.2-1.2] weeks). Figure 2 presents the final THIS developmental scale, including all evaluated milestones, the ages at which they were screened, and the observed success rate per task.

### Comparison Between THIS Developmental Scale and Milestone Norms of Other Screening Tools

**Figure 3.**
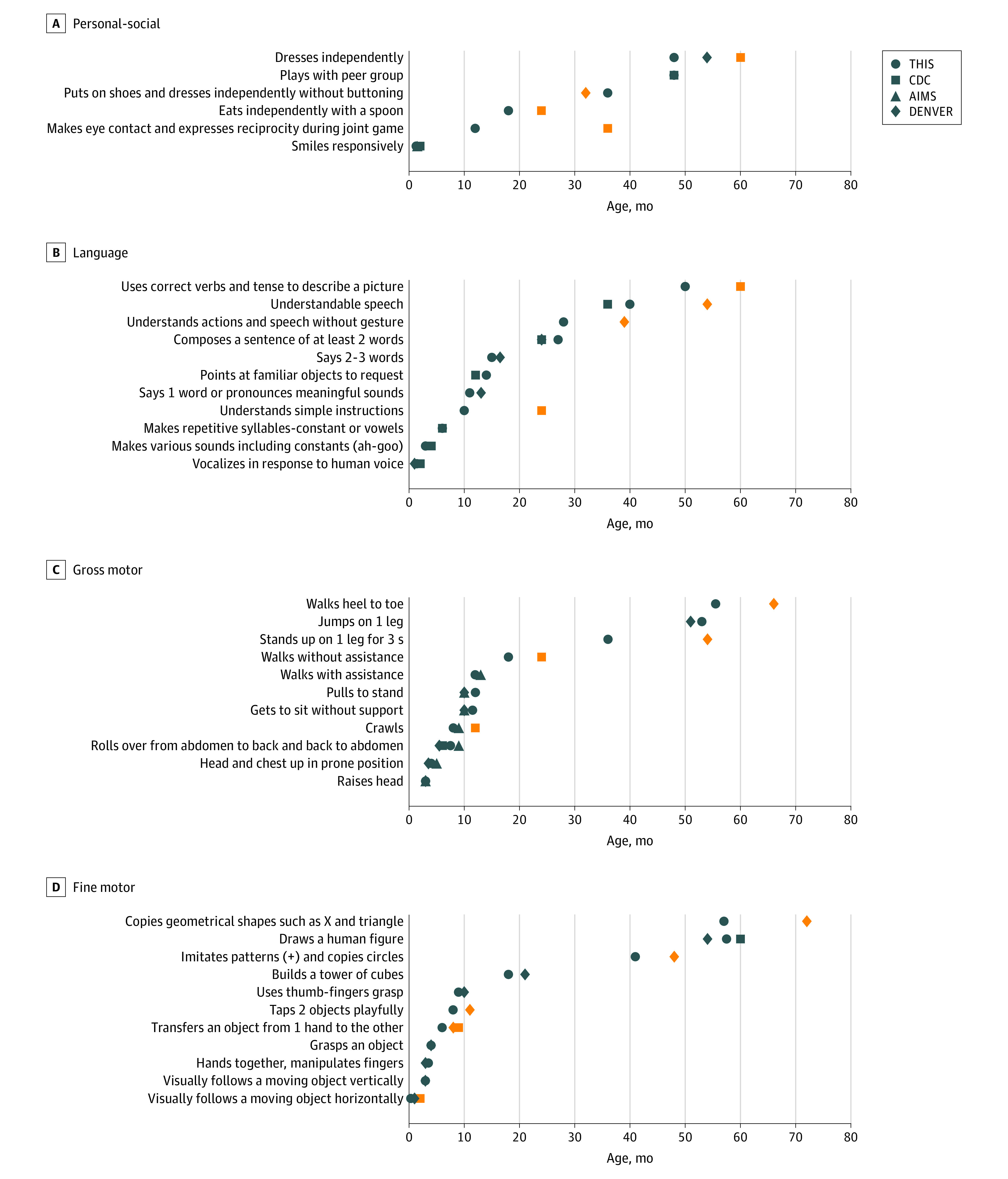
Comparison of Developmental Milestones Between Different Screening Tools Graphs show comparisons of the milestones at each field (personal-social, language, fine, and gross motor), between the Tipat Halav Israel Screening (THIS) developmental scale and the different commonly used screening tools (Centers for Disease Control and Prevention [CDC] Developmental Assessment, Alberta Infant Motor Scale [AIMS], and Denver Developmental Screening Test II [Denver]). The orange symbols represent milestones with clinically meaningful differences in achievement age between the different screening tools.

The THIS Developmental Scale was compared with milestones norms of 3 other commonly used developmental tests: Denver II, AIMS, and the CDC Developmental Assessment.^15,17,29^ Figure 3 presents a comparison of the equivalent milestones from the 4 scales, demonstrating a match of 18 of 27 milestones (67%) with the Denver II scale, 7 of 7 (100%) with the AIMS scale, and 10 of 19 (53%) with the CDC Developmental Assessment. The remaining unmatched milestones were achieved earlier in the THIS scale compared with the other screening tools.^15,17,29^

## Discussion

This cross-sectional study describes the establishment of milestones norms and building of THIS, an evidence-based developmental scale from birth to the age of 6 years. Updated performance norms were established according to a large national database of standardized developmental milestone assessments in the domains of gross motor, fine motor, language, and social skills. The THIS developmental scale represents the heterogeneous population comprising this cohort.

To the best of our knowledge, the current study is based on the largest population evaluated for developmental performance to date, including approximately 3.8 million evaluations conducted over a period of 6 years. Because Israel is a multicultural country, with a diverse population of multiple ethnicities and languages, we can use our representative data set to define subpopulations of interest, thereby enabling adapting of the scale for specific populations of interest.

The THIS developmental scale currently enables evaluation of milestone attainment age and comparison with the population norms, allowing early identification of potential delay and further guidance, surveillance, and in-depth evaluation when needed. In Israel, the standardized MoH protocol for MCHCs has been updated to reflect the findings of the current study. Further monitoring and fine-tuning of the updated scale, as well as validating THIS developmental scale performance as a screening tool for early detection of developmental delay, will be performed and made accessible for public use.

The THIS developmental scale was compared with milestone attainment ages for 3 commonly used developmental tests.^15,17,29^ The Denver II^17^ is widely performed by health care practitioners globally to identify children up to age 6 years with developmental problems, in low-risk populations. Denver II norms were established using data collected in 1988 to 1989. Among the 27 milestones that were comparable between THIS and Denver II, 18 were achieved during a similar time frame, and 9 were achieved earlier in the THIS developmental scale. The main discrepancies found were in fine motor skills, with mismatch of 5 milestones, alongside 2 discrepant milestones in each of gross motor and language tasks. It is yet to be determined whether these differences originate from nuances of the milestone evaluation method or from cultural disparities.

The AIMS^29^ is an observational assessment scale, constructed to specifically measure gross motor maturation in infants from birth through 18 months. It enables evaluation of motor developmental delay among high-risk populations. AIMS norms were established using data collected between 1989 and 1992. All 7 comparable milestones were achieved during a similar time frame in the THIS scale.

The THIS scale, Denver II, and AIMS all involve comprehensive evaluation based on developmental testing, structured observations, and interviews with the child’s parents, which require substantial resources of time and staff. In contrast, the CDC Developmental Assessment^15^ comprises a brief checklist of social, language, fine motor, and gross motor age-related milestones completed by the parents, alongside a list of alert signs—that is, milestones that require further medical evaluation if not accomplished by a certain age. The CDC data were collected between 2004 and 2008. Because using brief questionnaires is easier and more feasible for worldwide use, the THIS scale was also compared with the CDC Developmental Assessment. Because the CDC Developmental Assessment differs from the Denver II and AIMS by not providing the exact age in which 90% of children fail to achieve each milestone, the comparison may hold some discrepancies. Of 19 milestones that were comparable to THIS, only 10 were achieved during a similar time frame, whereas 9 were achieved earlier in the THIS developmental scale. Because only 53% of the comparable milestones were achieved during a similar time frame, the CDC Developmental Assessment might require further fine-tuning. Earlier studies demonstrated inconsistency between the CDC Developmental Assessment and other screening tools,^18^ which is in agreement with our findings. The observed changes between the various scales might be attributed to several factors, such as possible differences of the populations characteristics, the exact manner in which the milestones were evaluated, and the gap of 15 to 30 years between the contemporary data presented in this study and the other tools.

Further study is required to examine the efficacy of the THIS developmental scale in screening low-risk populations for developmental delays. Furthermore, it is essential to evaluate the effect of demographic variables, such as cultural ones, maternal education, socioeconomic status, or sex, on the success rate of the milestones, to enable a better culture-adjusted and personalized developmental evaluation.

### Limitations

Our study has several limitations. First, the primary objective of this study was to standardize developmental norms for a healthy population. However, the EMRs on which this study is based do not include data regarding genetic syndromes or other congenital anomalies that may result in developmental delay. We have minimized the influence of this gap by excluding children with abnormal developmental potential, including preterm infants, those with low birth weight, and those with abnormal weight or head circumference measurements, which may reflect factors associated with the risk of developmental delay. Second, among 59 evaluated milestones, 18 had already exceeded 95% success rates during initial assessment. This gap may indicate that these milestones are normally achieved at an earlier age; however, because they were not screened for earlier, we lack these normative data. Third, although the new THIS developmental scale includes milestones that are evaluated at Israeli MCHCs, many additional, potentially important, milestones were excluded because of the technical difficulty of performing wide developmental screening assessments to the entire children population regularly. An effort was made to include a balanced set of milestones, representing 4 developmental domains at each age group.

## Conclusions

The current study describes the validation and establishment of new milestones norms, composing the THIS developmental scale, an evidence-based developmental scale from birth to the age of 6 years. The THIS developmental scale is based on the largest population evaluated to date for developmental performance, representing the heterogeneous, multicultural population comprising this cohort. It is recommended for further evaluation worldwide.

## Notes

Supplement. **eFigure 1. **Task Performance Results Over Time at the Age 0-3 Months **eFigure 2.** Task Performance Results Over Time at the Age 3-6 Months **eFigure 3.** Task Performance Results Over Time at the Age 6-9 Months **eFigure 4.** Task Performance Results Over Time at the Age 9-12 Months **eFigure 5.** Task Performance Results Over Time at the Age 12-18 Months **eFigure 6.** Task Performance Results Over Time at the Age 18-24 Months **eFigure 7.** Task Performance Results Over Time at the Age 2-3 Years (24-36 Months) **eFigure 8.** Task Performance Results Over Time at the Age 3-4 Years (36-48 Months) **eFigure 9.** Task Performance Results Over Time at the Age 4-6 Years (48-72 Months) **eFigure 10.** Comparison of Milestone Achievement Threshold by Clinical Observation and Parental Report Versus Clinical Observation Alone **eTable 1.** Israeli Ministry of Health: A Guide to Performing Developmental Assessments for Infants and Toddlers From Birth up to the Age of Six Years **eTable 2.** Population Analysis of Children Evaluated at Each Visit at the Age 0-3 Months **eTable 3.** Population Analysis of Children Evaluated at Each Visit at the Age 3-6 Months **eTable 4.** Population Analysis of Children Evaluated at Each Visit at the Age 6-9 Months **eTable 5.** Population Analysis of Children Evaluated at Each Visit at the Age 9-12 Months **eTable 6.** Population Analysis of Children Evaluated at Each Visit at the Age 12-18 Months **eTable 7.** Population Analysis of Children Evaluated at Each Visit at the Age 18-24 Months **eTable 8.** Population Analysis of Children Evaluated at Each Visit at the Age 2-3 Years (24-36 Months) **eTable 9.** Population Analysis of Children Evaluated at Each Visit at the Age 3-4 Years (36-48 Months) **eTable 10.** Population Analysis of Children Evaluated at Each Visit at the Age 4-6 Years (48-72 Months) **eTable 11.** Comparison of the Characteristics of Evaluated Children Between the Ages 0-3 Years and 3-6 Years
