# Supplementary material for: Standardization of a Developmental Milestone Scale Using Data From Children in Israel
Source: JAMA Netw Open. 2022 Mar 14;5(3):e222184. doi: 10.1001/jamanetworkopen.2022.2184 (PMC9907346; doi:10.1001/jamanetworkopen.2022.2184)
Supplement: Supplement. — eFigure 1. Task Performance Results Over Time at the Age 0-3 Months eFigure 2. Task Performance Results Over Time at the Age 3-6 Months eFigure 3. Task Performance Results Over Time at the Age 6-9 Months eFigure 4. Task Performance Results Over Time at the Age 9-12 Months eFigure 5. Task Performance Results Over Time at the Age 12-18 Months eFigure 6. Task Performance Results Over Time at the Age 18-24 Months eFigure 7. Task Performance Results Over Time at the Age 2-3 Years (24-36 Months) eFigure 8. Task Performance Results Over Time at the Age 3-4 Years (36-48 Months) eFigure 9. Task Performance Results Over Time at the Age 4-6 Years (48-72 Months) eFigure 10. Comparison of Milestone Achievement Threshold by Clinical Observation and Parental Report Versus Clinical Observation Alone eTable 1. Israeli Ministry of Health: A Guide to Performing Developmental Assessments for Infants and Toddlers From Birth up to the Age of Six Years eTable 2. Population Analysis of Children Evaluated at Each Visit at the Age 0-3 Months eTable 3. Population Analysis of Children Evaluated at Each Visit at the Age 3-6 Months eTable 4. Population Analysis of Children Evaluated at Each Visit at the Age 6-9 Months eTable 5. Population Analysis of Children Evaluated at Each Visit at the Age 9-12 Months eTable 6. Population Analysis of Children Evaluated at Each Visit at the Age 12-18 Months eTable 7. Population Analysis of Children Evaluated at Each Visit at the Age 18-24 Months eTable 8. Population Analysis of Children Evaluated at Each Visit at the Age 2-3 Years (24-36 Months) eTable 9. Population Analysis of Children Evaluated at Each Visit at the Age 3-4 Years (36-48 Months) eTable 10. Population Analysis of Children Evaluated at Each Visit at the Age 4-6 Years (48-72 Months) eTable 11. Comparison of the Characteristics of Evaluated Children Between the Ages 0-3 Years and 3-6 Years [file jamanetwopen-e222184-s001.pdf]

Supplemental Online Content

Sudry T, Zimmerman DR, Yardeni H, et al. Standardization of a developmental milestone scale using data from children in Israel. *JAMA Netw Open*. 2022;5(3):e222184. doi:10.1001/jamanetworkopen.2022.2184

eFigure 1. Task Performance Results Over Time at the Age 0-3 Months

eFigure 2. Task Performance Results Over Time at the Age 3-6 Months

eFigure 3. Task Performance Results Over Time at the Age 6-9 Months

eFigure 4. Task Performance Results Over Time at the Age 9-12 Months

eFigure 5. Task Performance Results Over Time at the Age 12-18 Months

eFigure 6. Task Performance Results Over Time at the Age 18-24 Months

eFigure 7. Task Performance Results Over Time at the Age 2-3 Years (24-36 Months)

eFigure 8. Task Performance Results Over Time at the Age 3-4 Years (36-48 Months)

eFigure 9. Task Performance Results Over Time at the Age 4-6 Years (48-72 Months)

eFigure 10. Comparison of Milestone Achievement Threshold by Clinical Observation and Parental Report Versus Clinical Observation Alone

eTable 1. Israeli Ministry of Health: A Guide to Performing Developmental Assessments for Infants and Toddlers From Birth up to the Age of Six Years

eTable 2. Population Analysis of Children Evaluated at Each Visit at the Age 0-3 Months

eTable 3. Population Analysis of Children Evaluated at Each Visit at the Age 3-6 Months

eTable 4. Population Analysis of Children Evaluated at Each Visit at the Age 6-9 Months

eTable 5. Population Analysis of Children Evaluated at Each Visit at the Age 9-12 Months

eTable 6. Population Analysis of Children Evaluated at Each Visit at the Age 12-18 Months

eTable 7. Population Analysis of Children Evaluated at Each Visit at the Age 18-24 Months

eTable 8. Population Analysis of Children Evaluated at Each Visit at the Age 2-3 Years (24-36 Months)

eTable 9. Population Analysis of Children Evaluated at Each Visit at the Age 3-4 Years (36-48 Months)

eTable 10. Population Analysis of Children Evaluated at Each Visit at the Age 4-6 Years (48-72 Months)

eTable 11. Comparison of the Characteristics of Evaluated Children Between the Ages 0-3 Years and 3-6 Years

This supplemental material has been provided by the authors to give readers additional information about their work.

© 2022 Sudry T et al. *JAMA Network Open.*

eFigure 1. Task Performance Results Over Time at the Age 0-3 Months

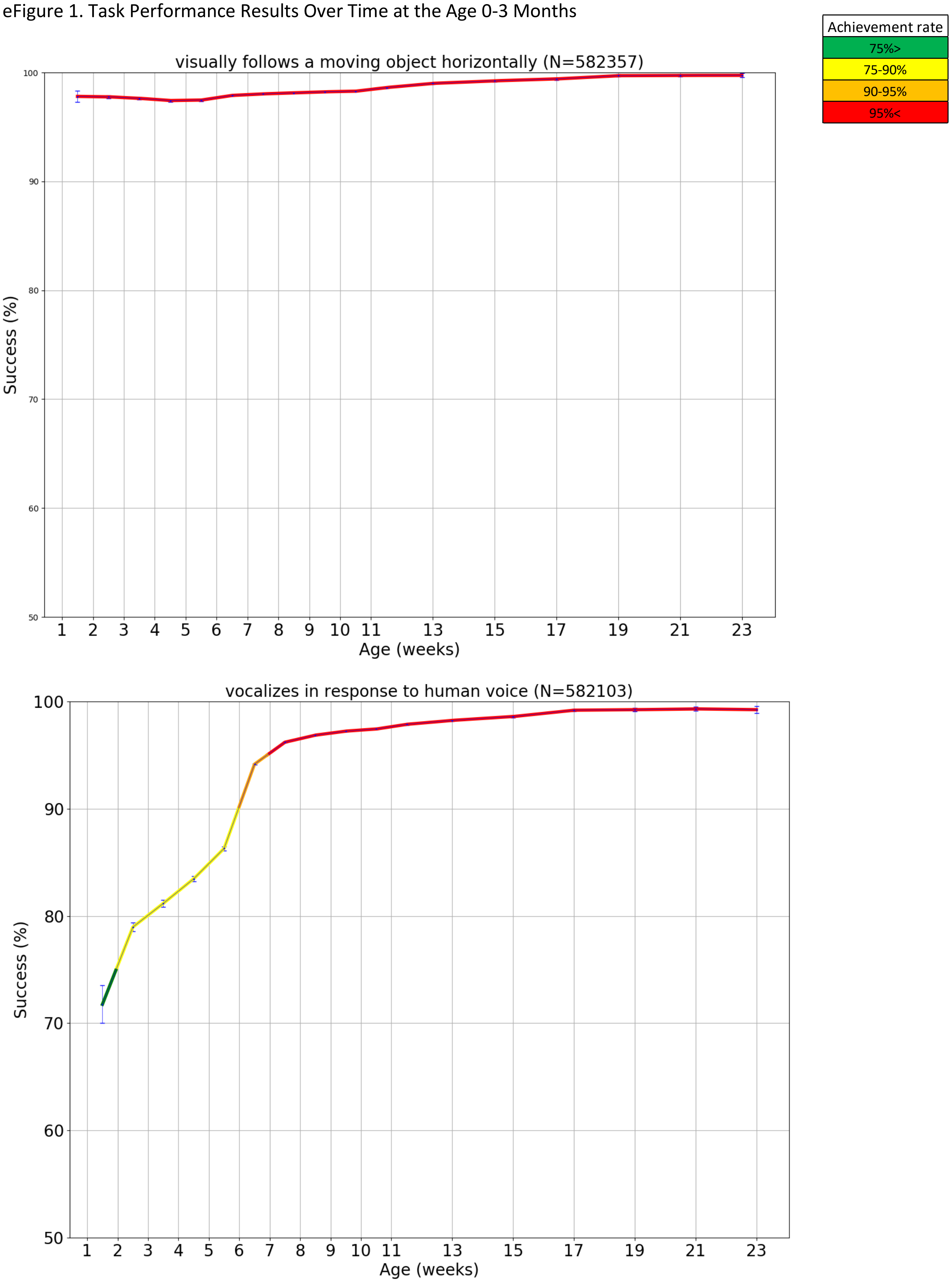

© 2022 Sudry T et al. *JAMA Network Open.*

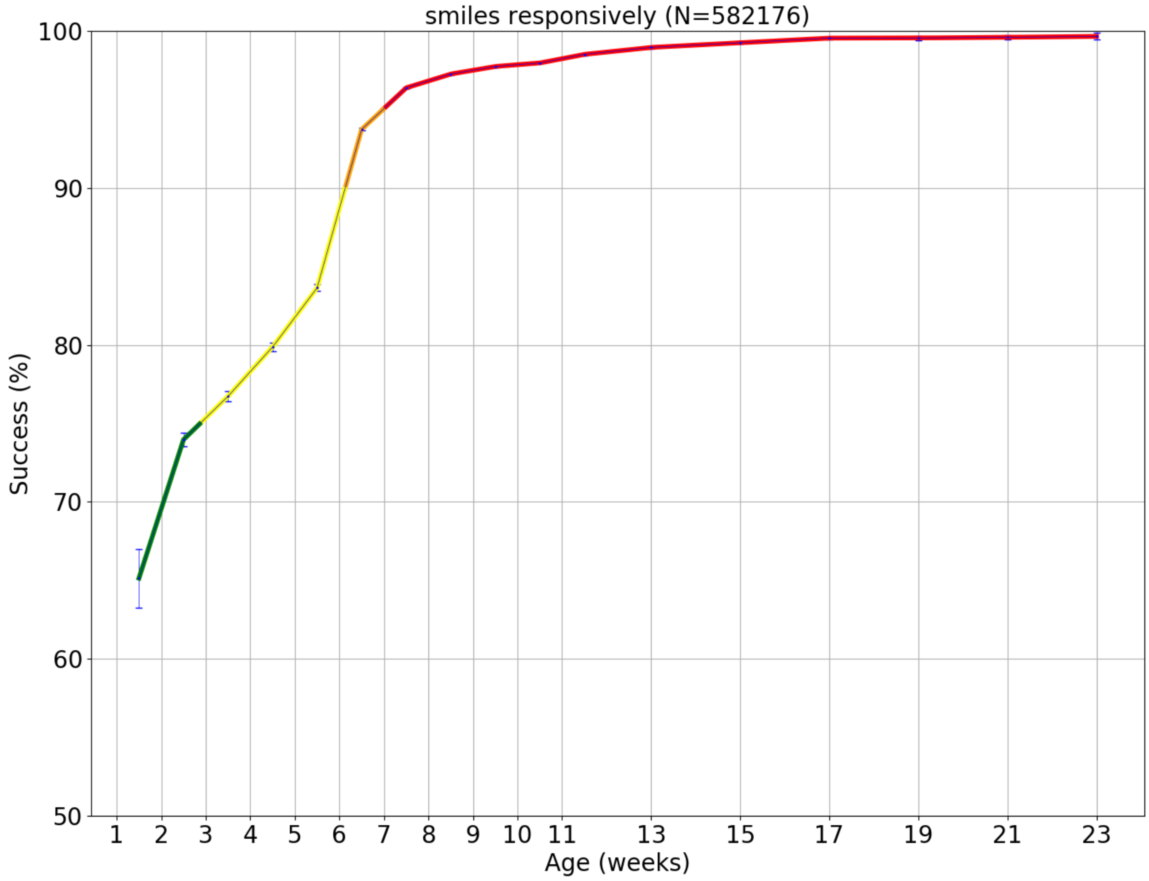

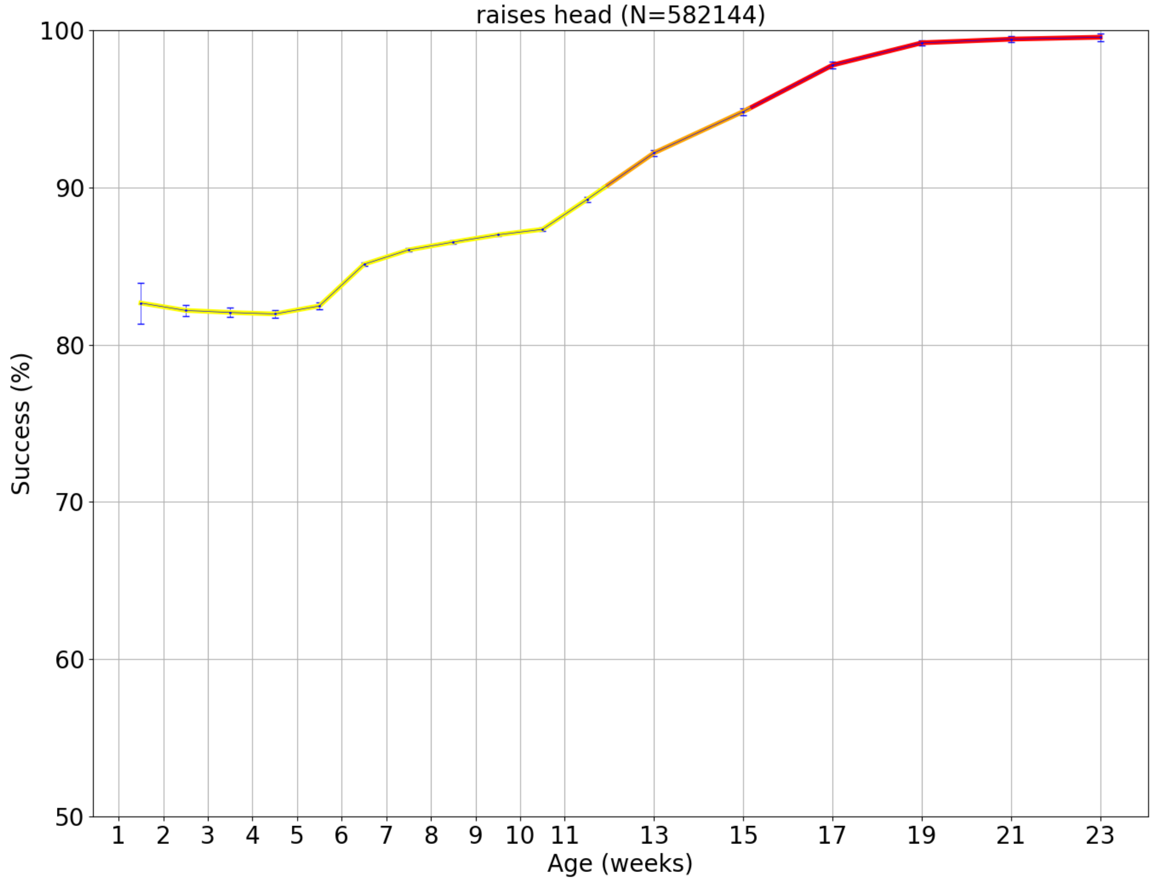

eFigure 1: The success rates over time of the different milestones evaluated between the ages 0-3 months. The achievement rates of < 75%, 75-90%, 90-95% and > 95% of children are indicated by the colors green, yellow, orange and red, respectively. The error bars are 95% confidence intervals of a binomial proportion, where larger bars indicate smaller sample size.

© 2022 Sudry T et al. *JAMA Network Open.*

eFigure 2. Task Performance Results Over Time at the Age 3-6 Months

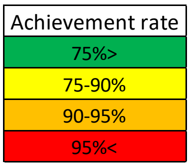

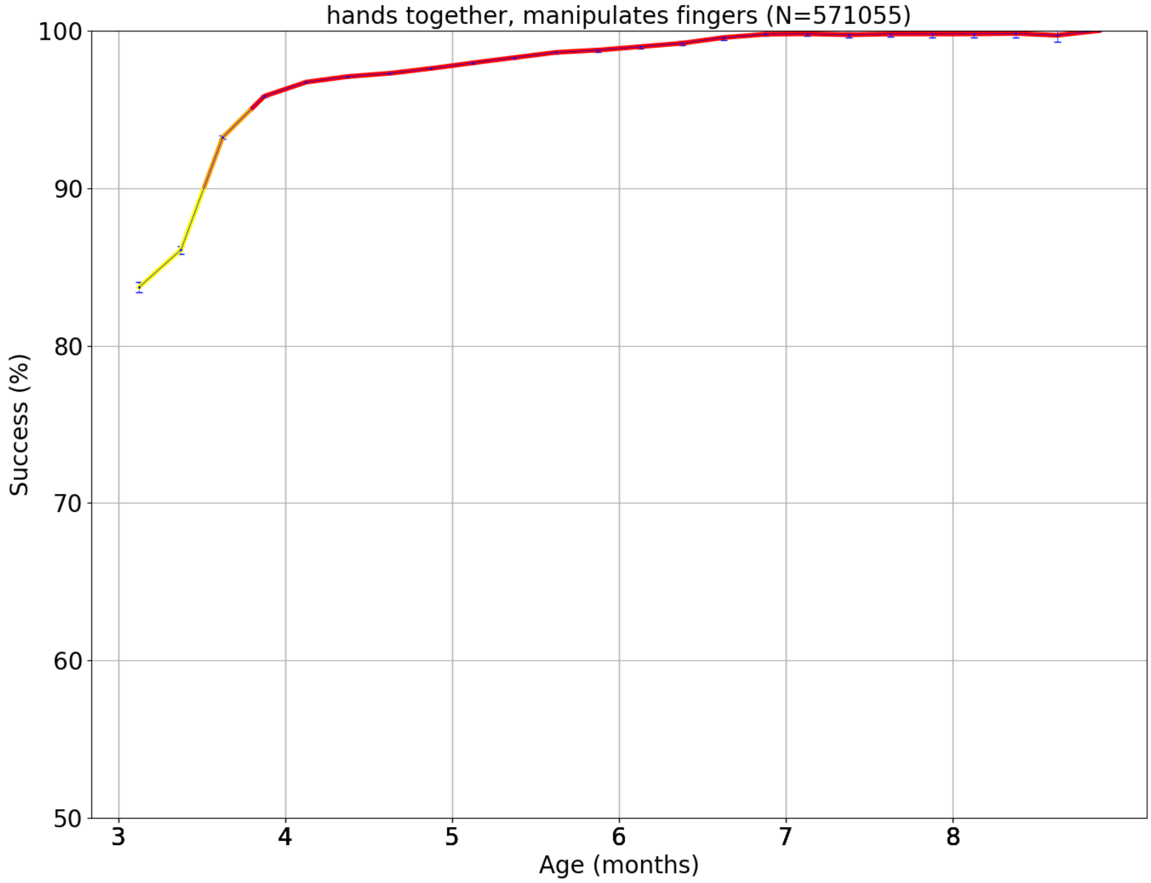

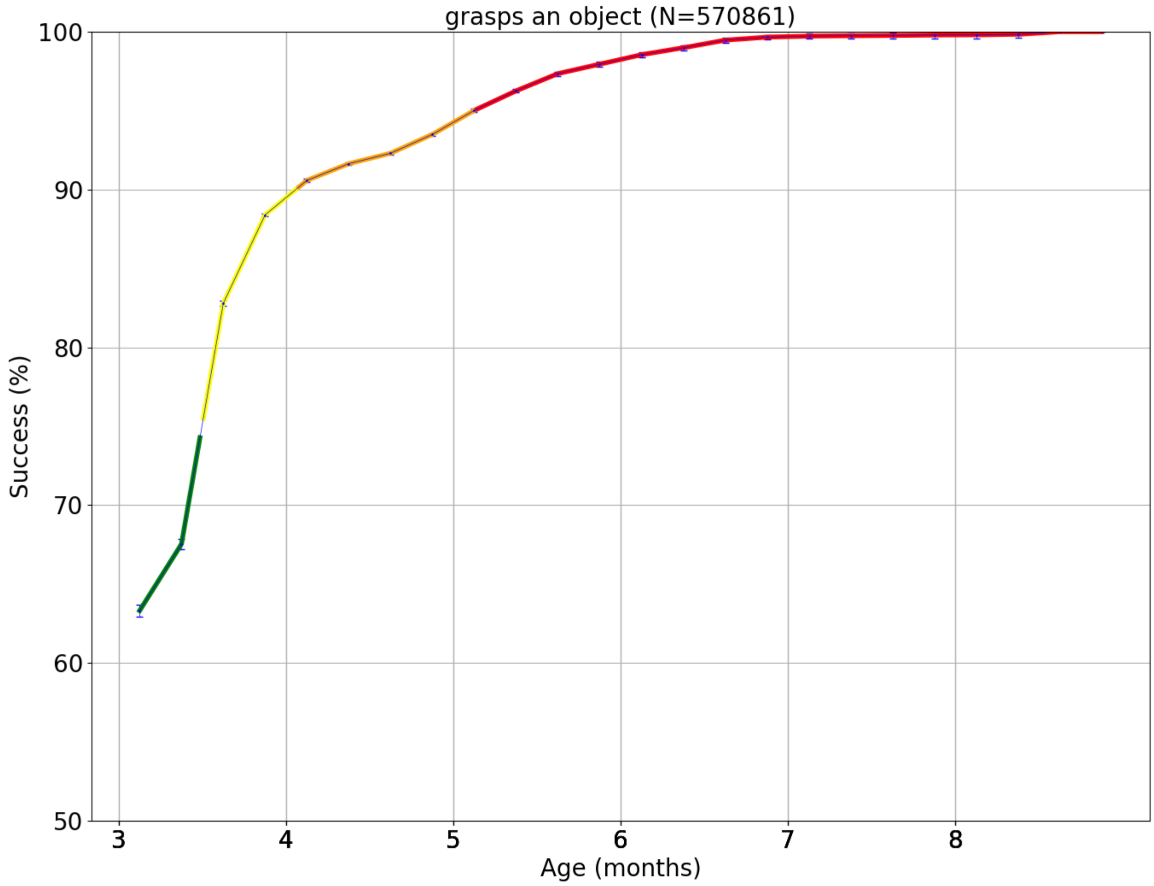

© 2022 Sudry T et al. *JAMA Network Open.*

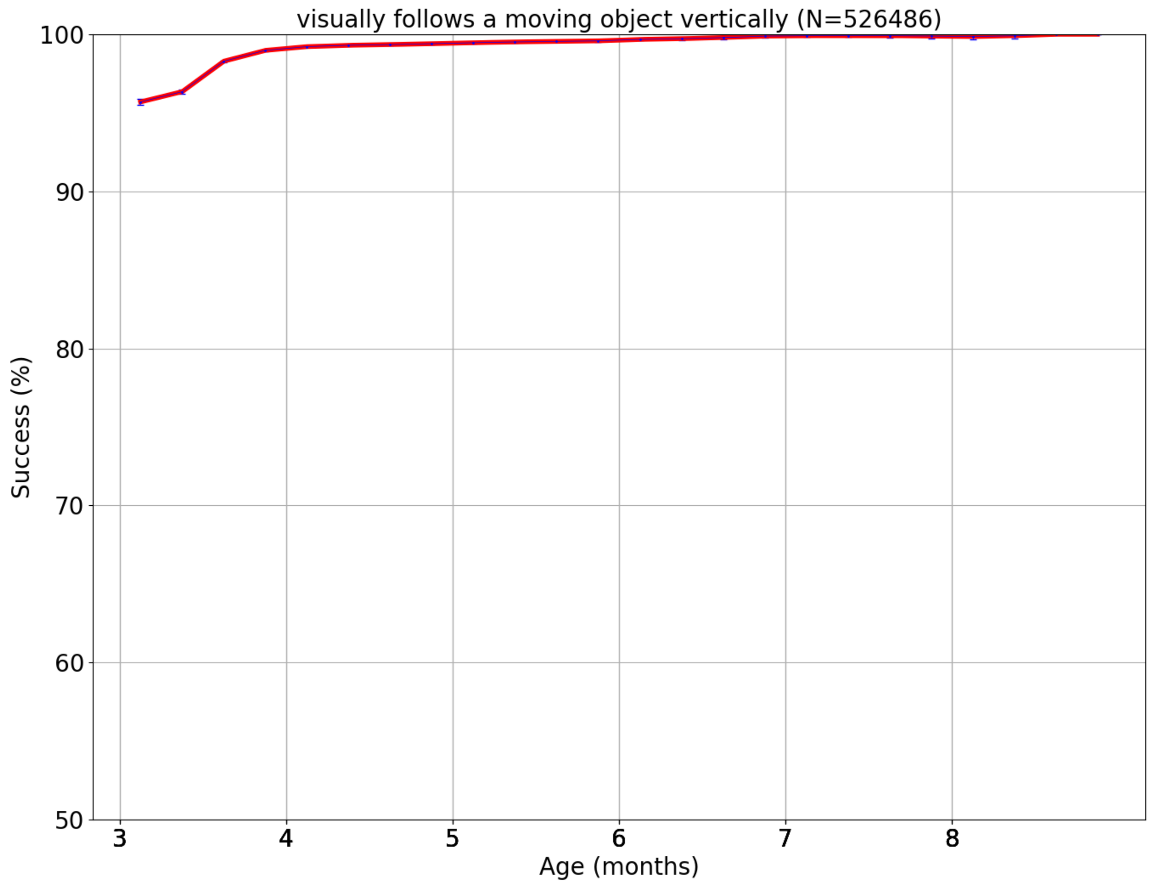

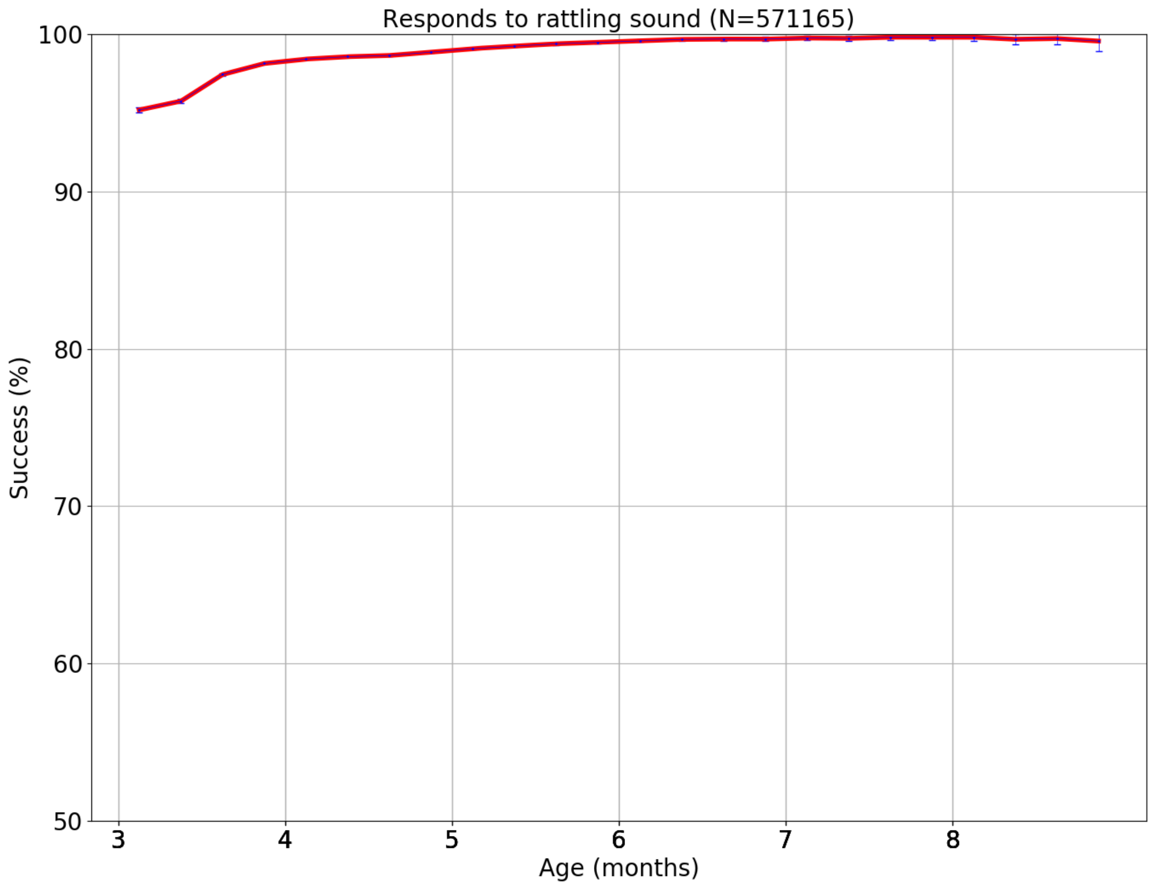

© 2022 Sudry T et al. *JAMA Network Open.*

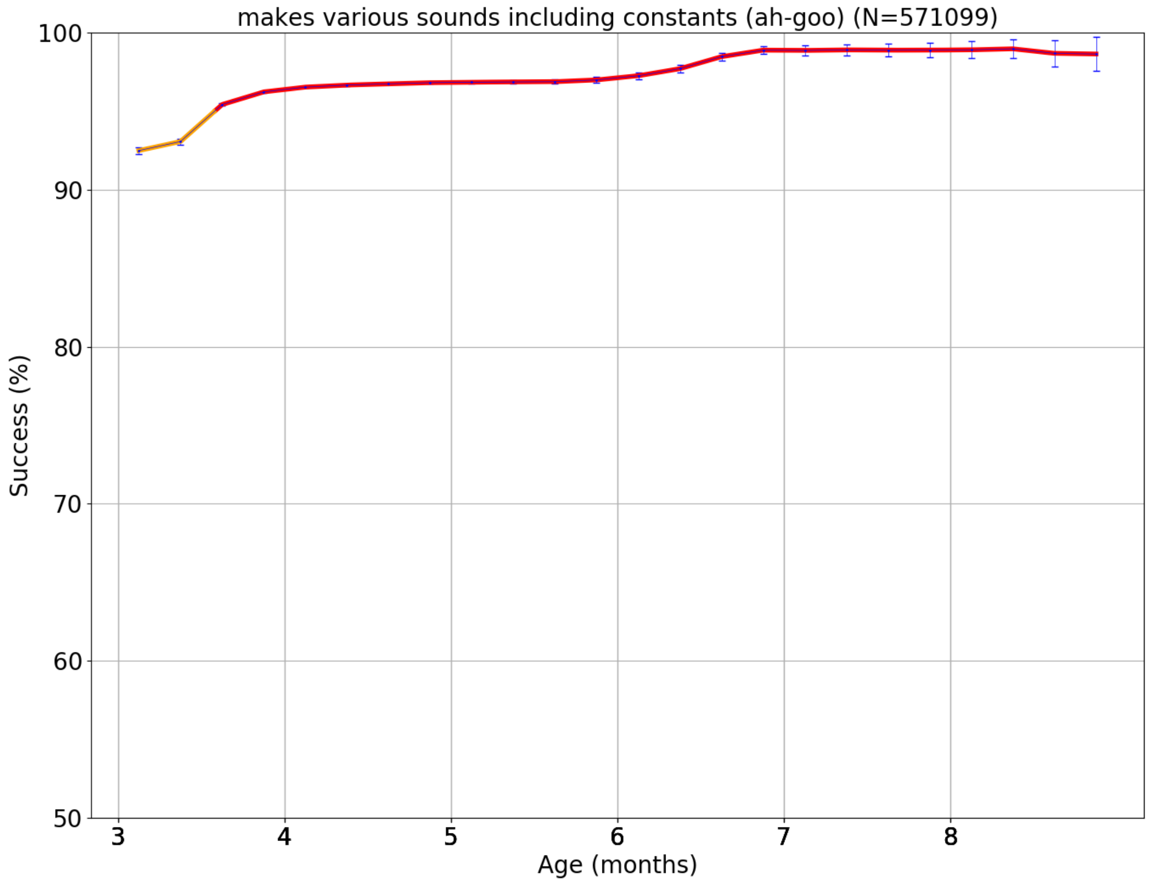

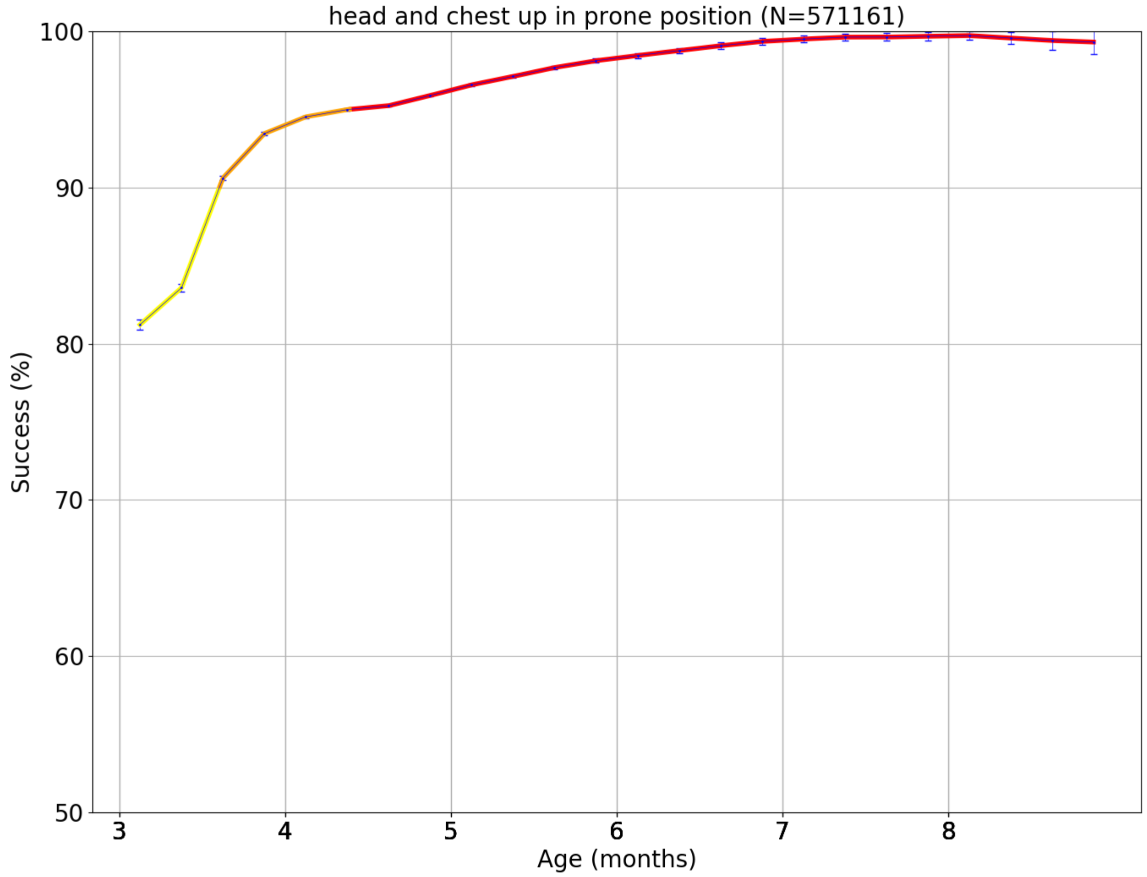

eFigure 2: The success rates over time of the different milestones evaluated between the ages 3-6 months. The achievement rates of < 75%, 75-90%, 90-95% and > 95% of children are indicated by the colors green, yellow, orange and red, respectively. The error bars are 95% confidence intervals of a binomial proportion, where larger bars indicate smaller sample size.

© 2022 Sudry T et al. *JAMA Network Open.*

Achievement rate
75%>
75-90%
90-95%
95%<

eFigure 3. Task Performance Results Over Time at the Age 6-9 Months

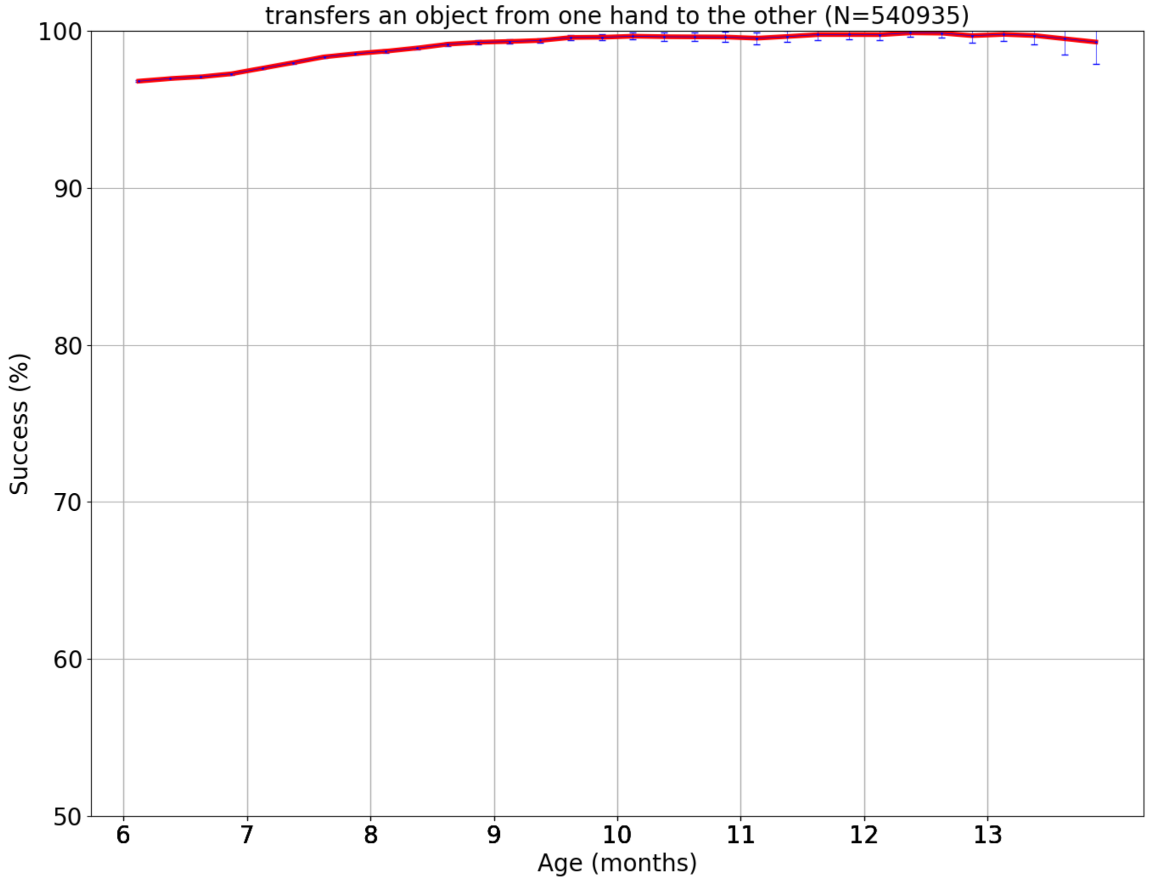

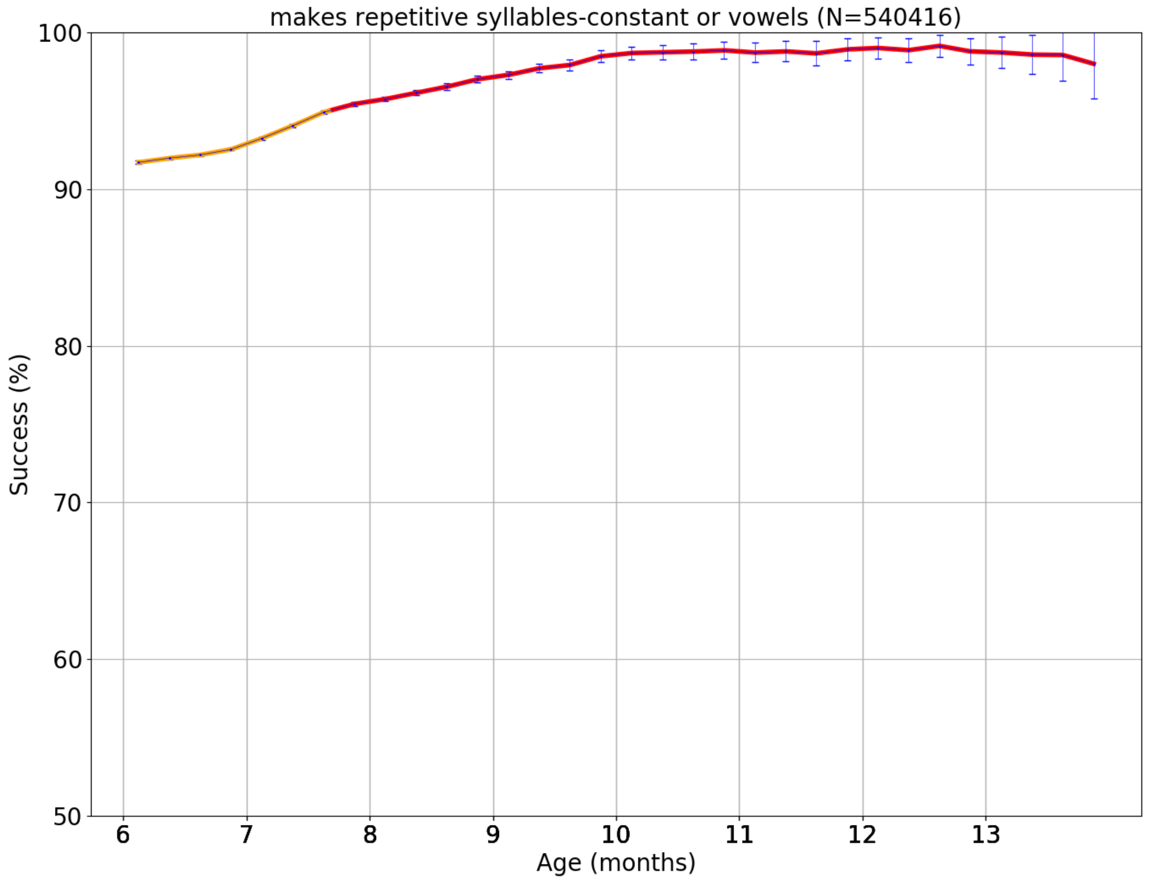

© 2022 Sudry T et al. *JAMA Network Open.*

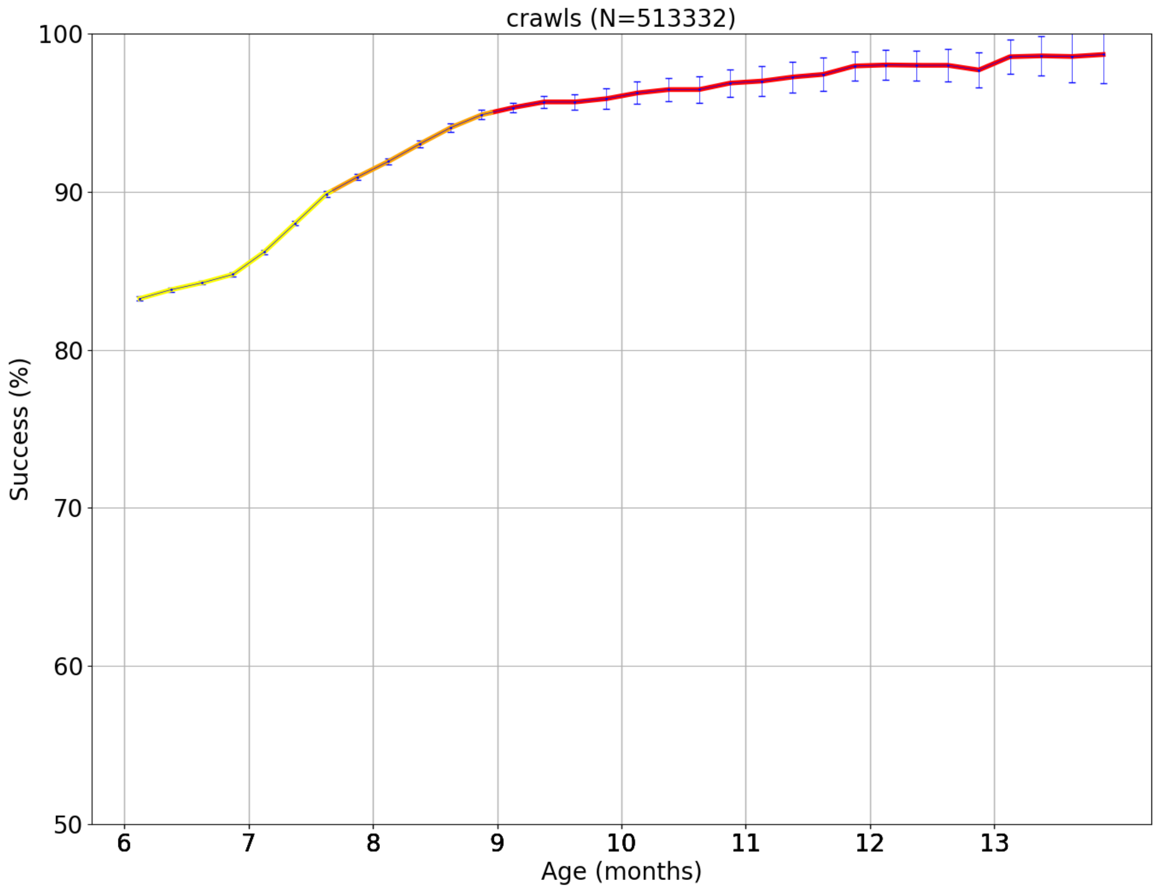

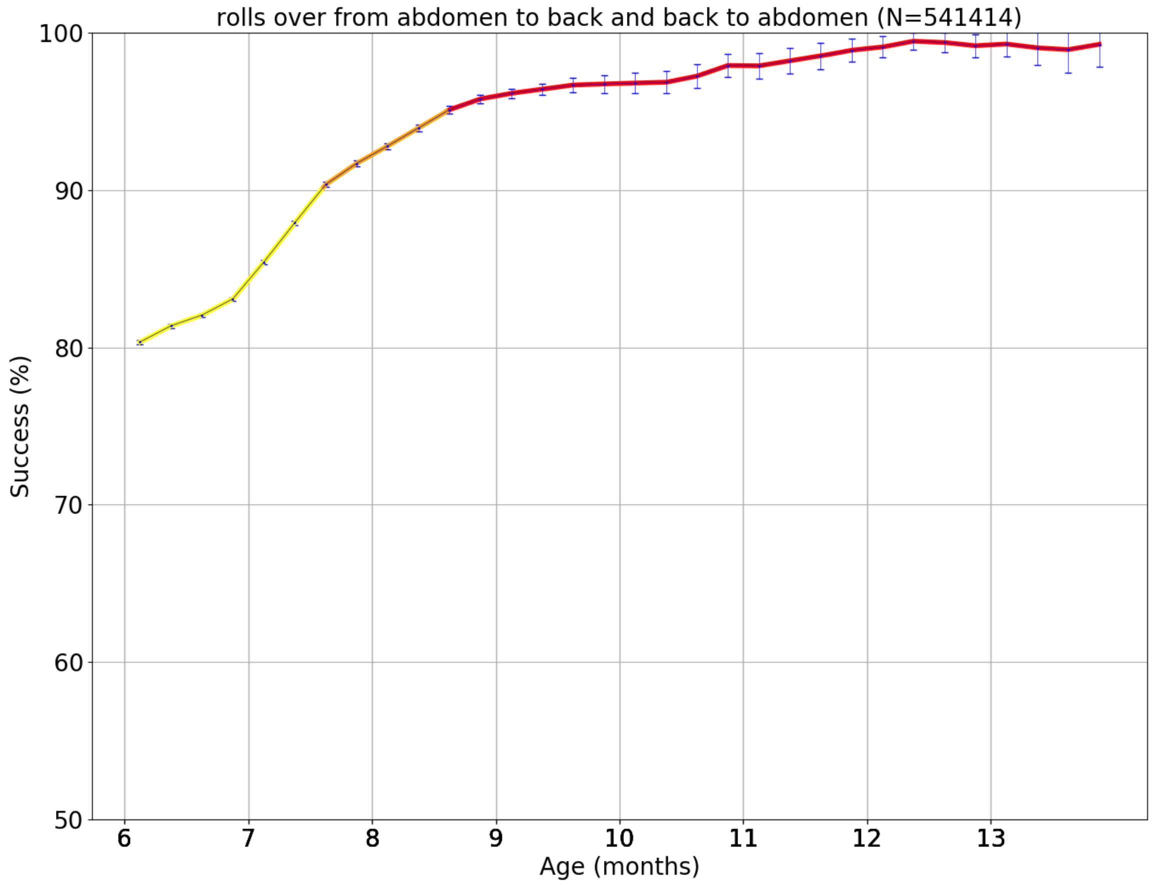

© 2022 Sudry T et al. *JAMA Network Open.*

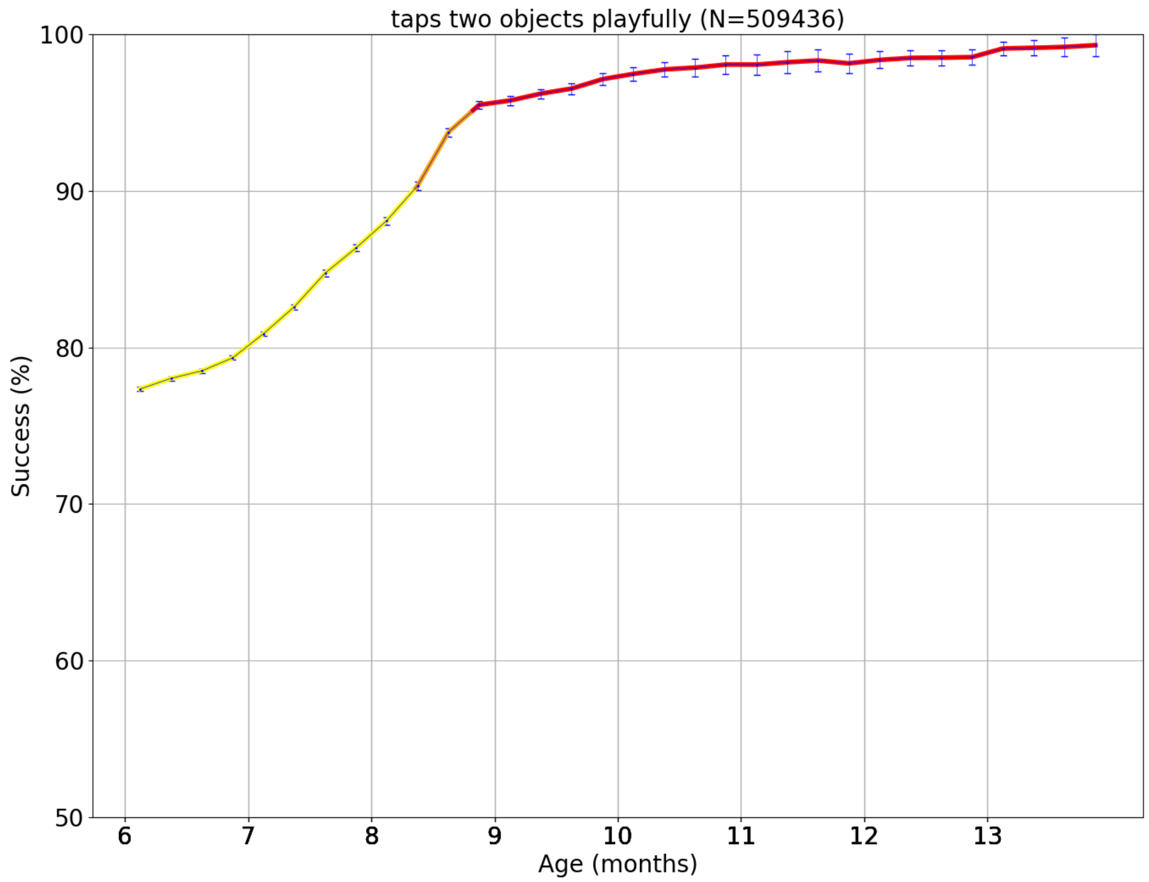

eFigure 3: The success rates over time of the different milestones evaluated between the ages 6-9 months. The achievement rates of < 75%, 75-90%, 90-95% and > 95% of children are indicated by the colors green, yellow, orange, and red, respectively. The error bars are 95% confidence intervals of a binomial proportion, where larger bars indicate smaller sample size.

© 2022 Sudry T et al. *JAMA Network Open.*

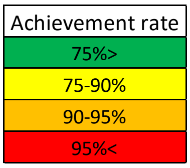

eFigure 4. Task Performance Results Over Time at the Age 9-12 Months

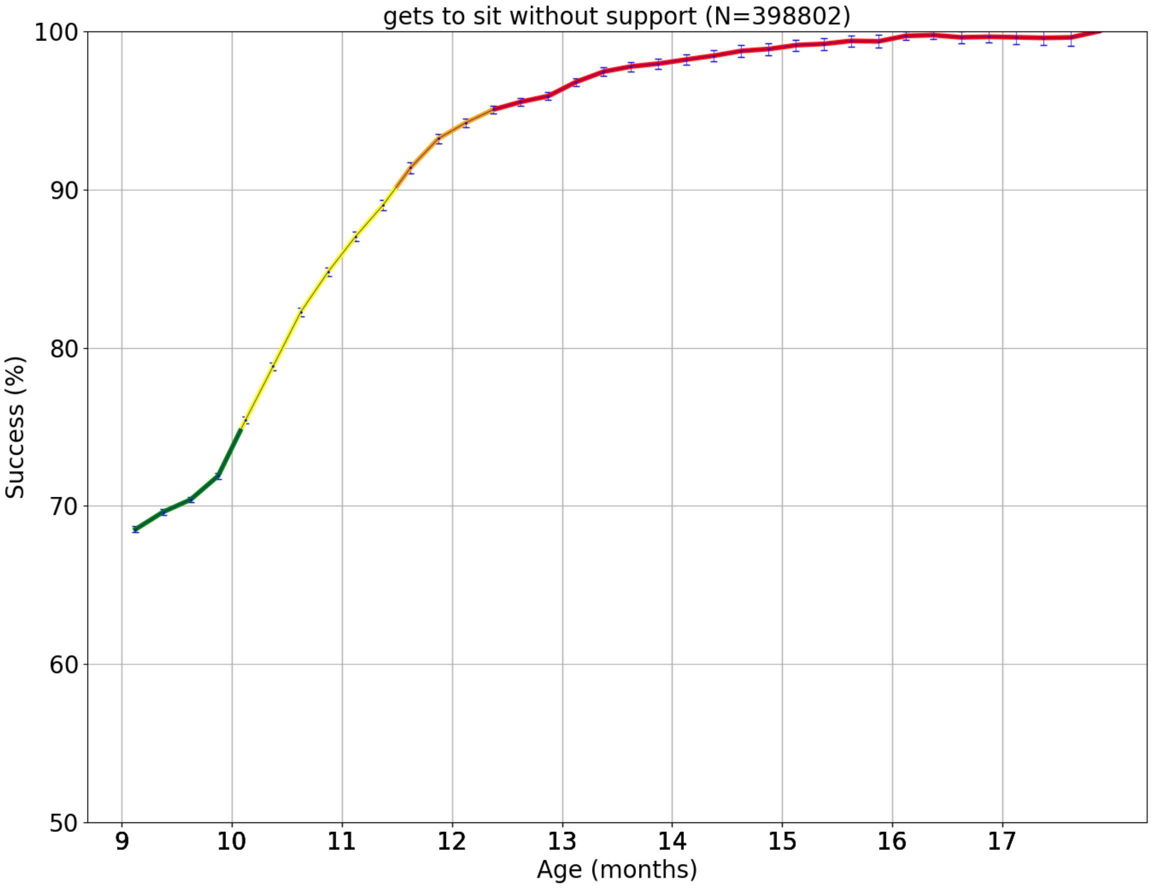

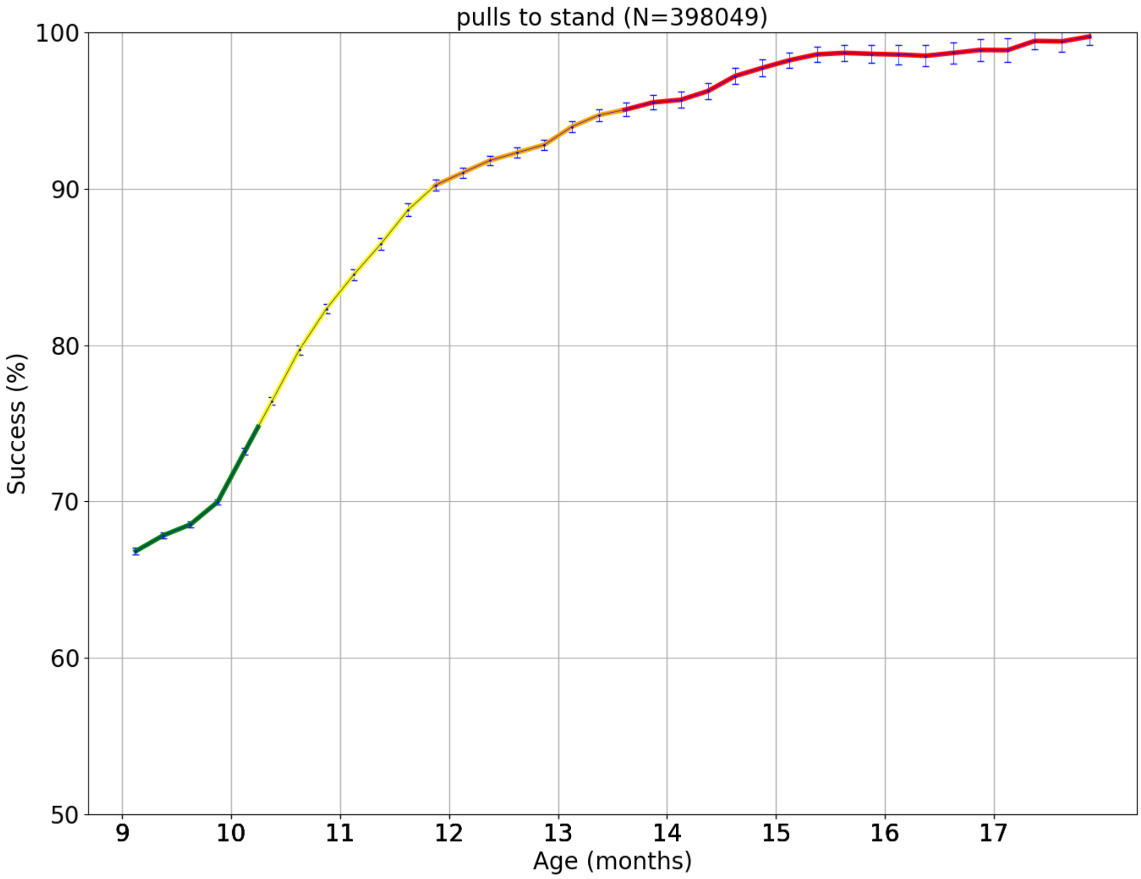

© 2022 Sudry T et al. *JAMA Network Open.*

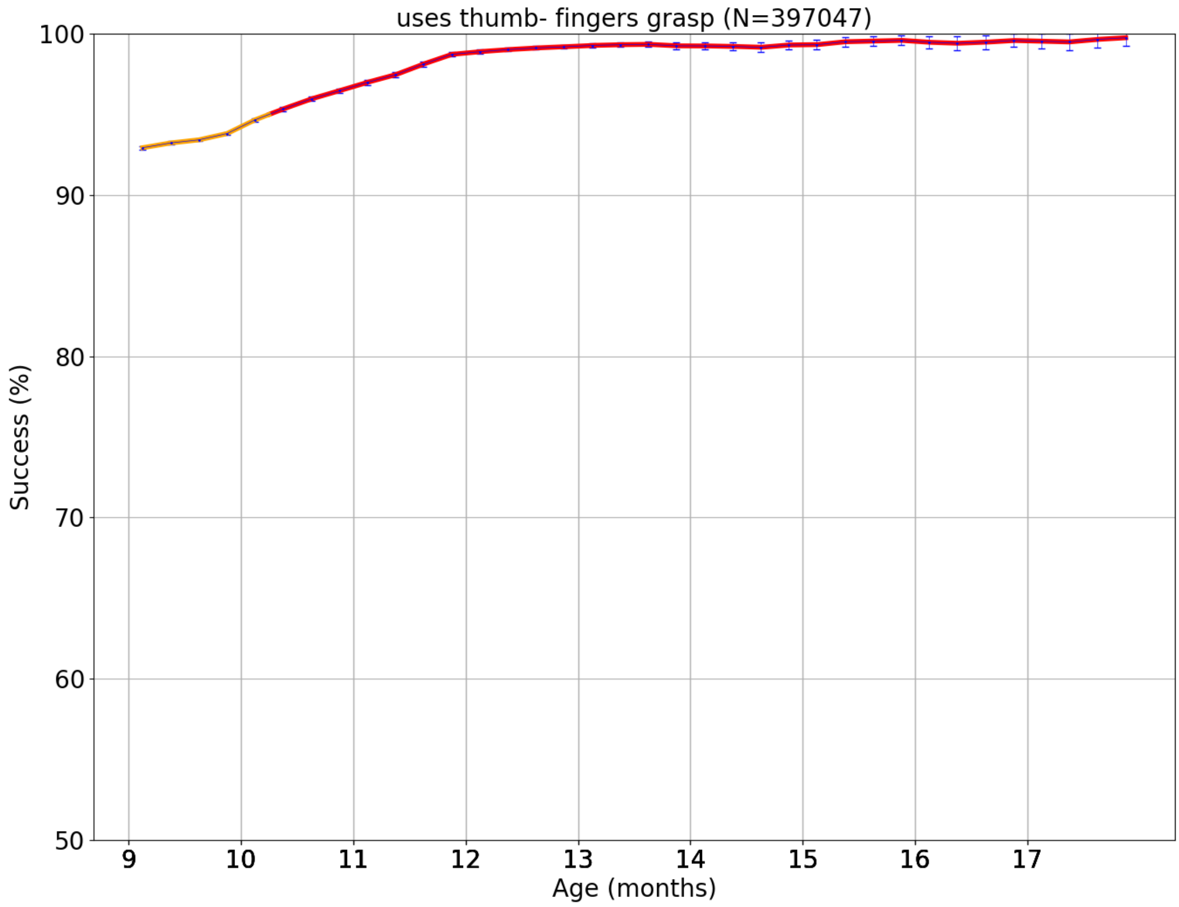

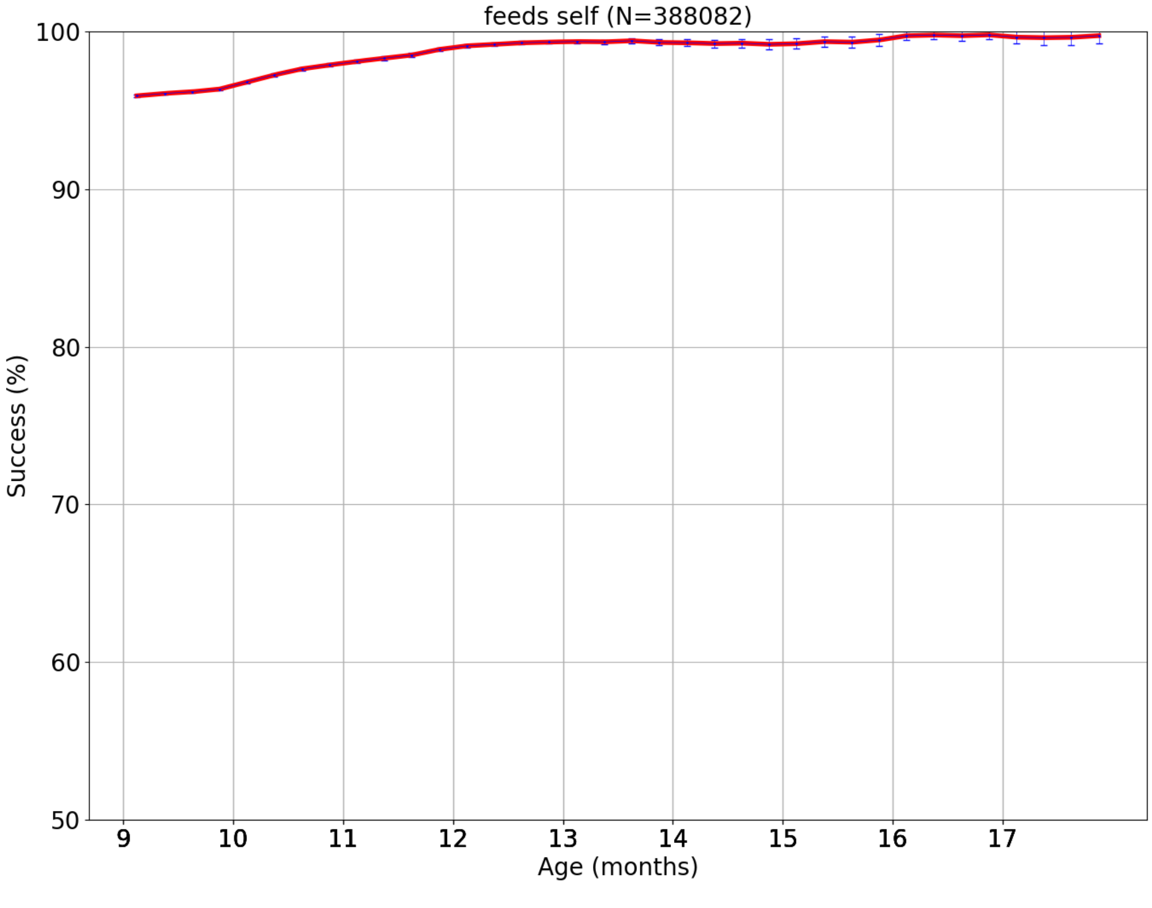

© 2022 Sudry T et al. *JAMA Network Open.*

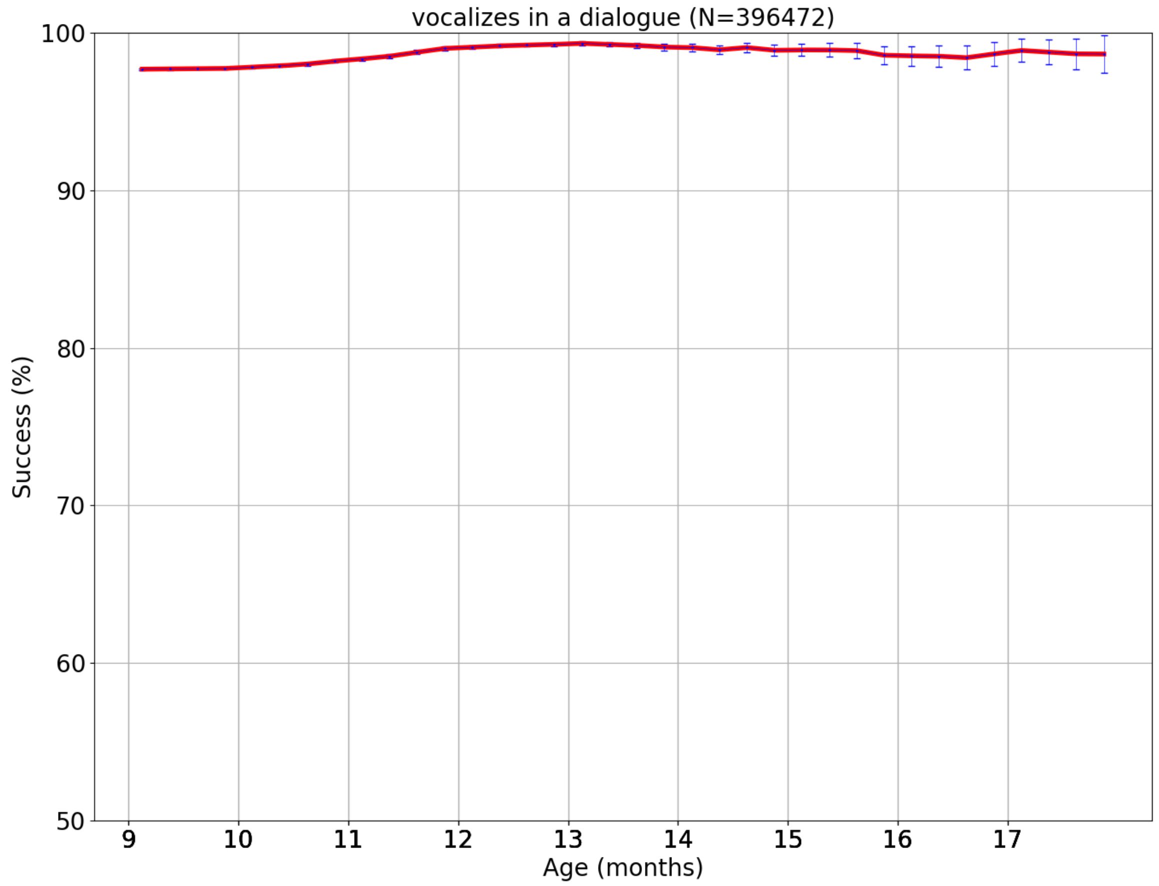

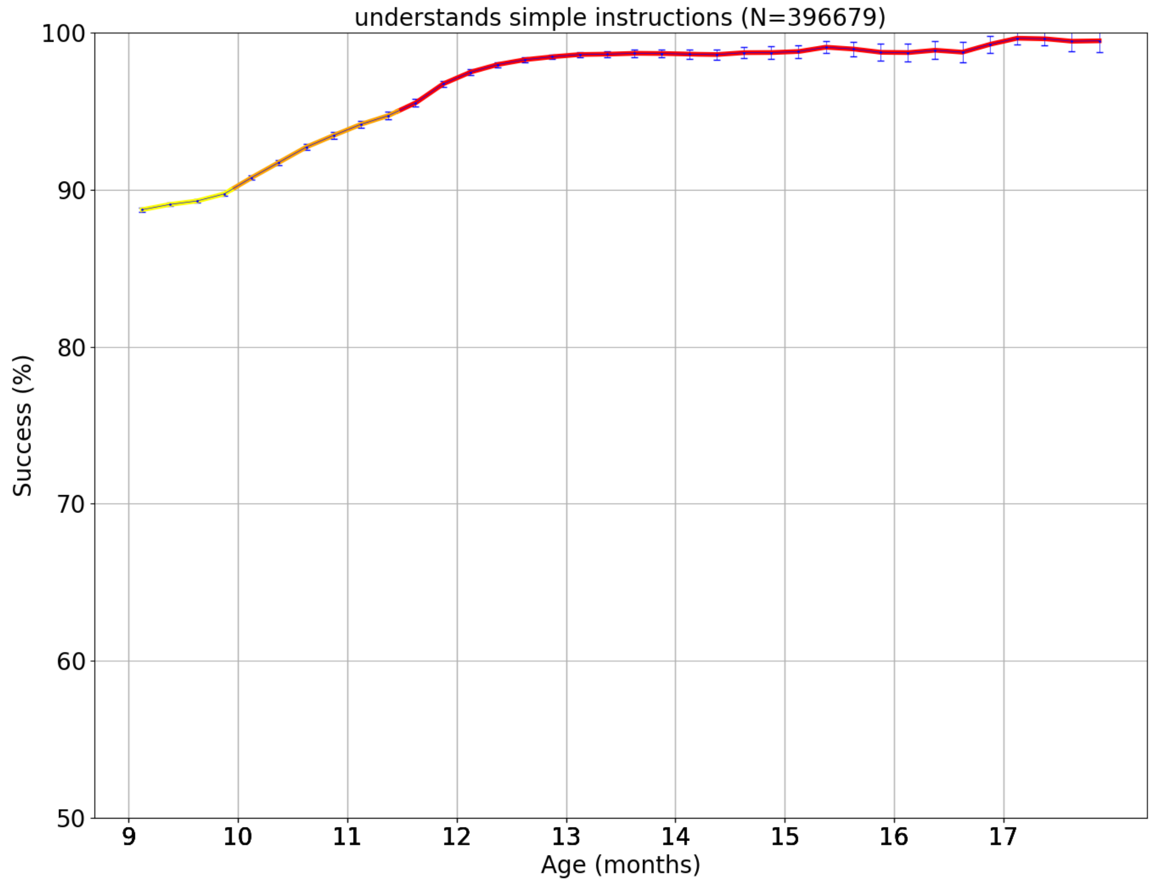

© 2022 Sudry T et al. *JAMA Network Open.*

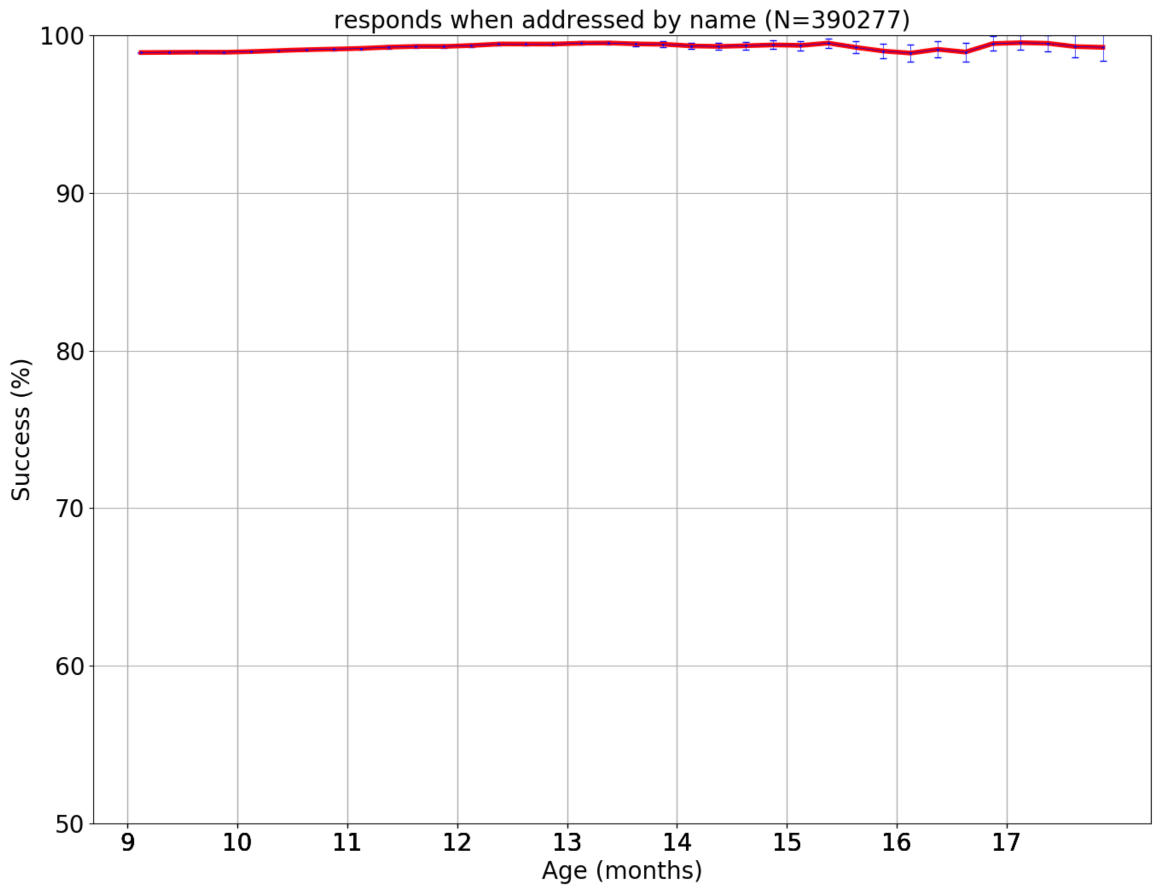

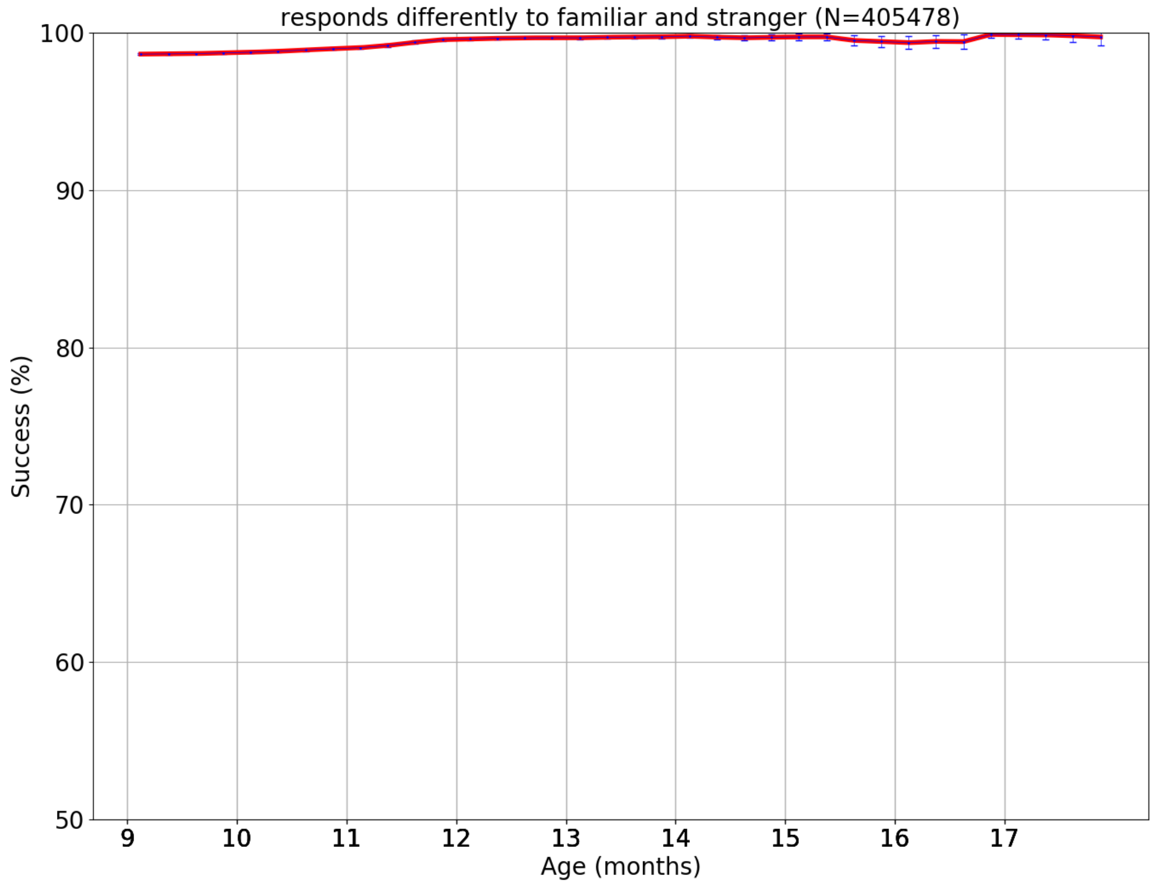

© 2022 Sudry T et al. *JAMA Network Open.*

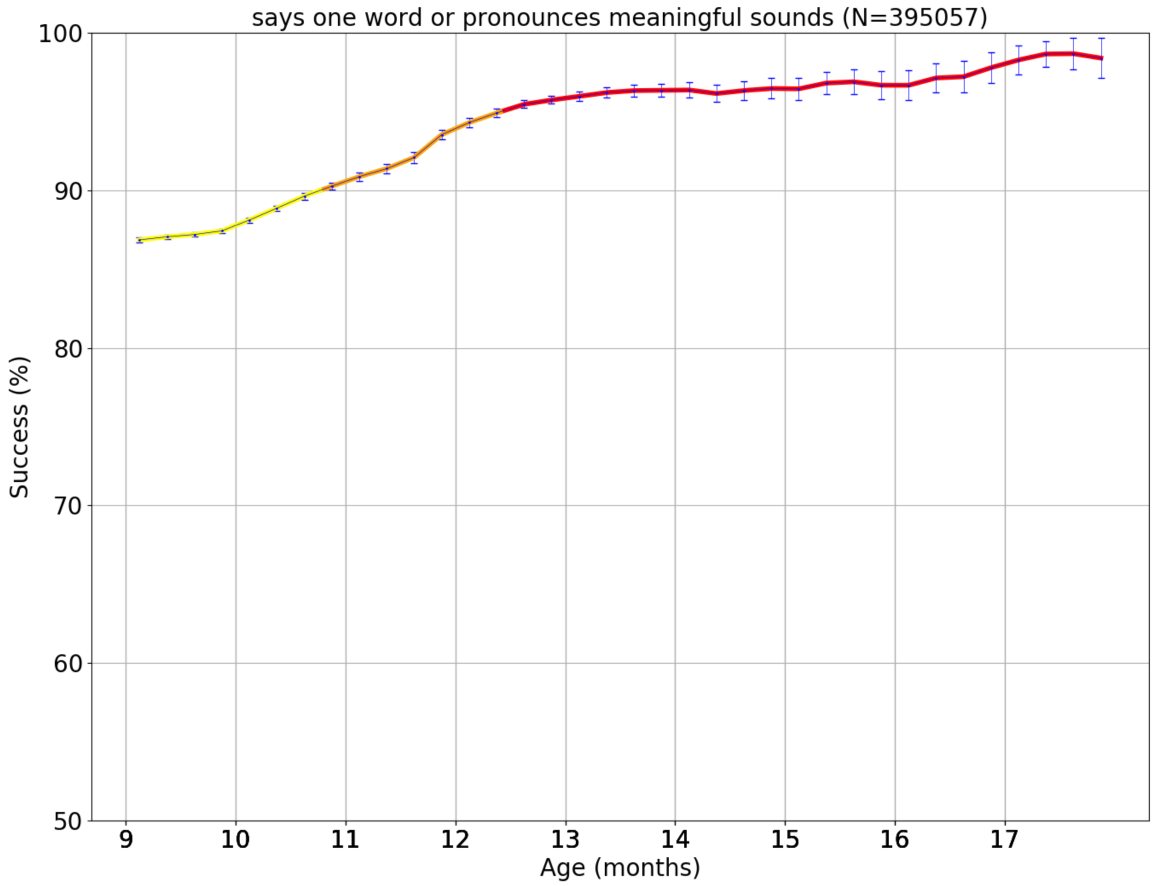

eFigure 4: The success rates over time of the different milestones evaluated between the ages 9-12 months. The achievement rates of < 75%, 75-90%, 90-95% and > 95% of children are indicated by the colors green, yellow, orange and red, respectively. The error bars are 95% confidence intervals of a binomial proportion, where larger bars indicate smaller sample size.

© 2022 Sudry T et al. *JAMA Network Open.*

Achievement rate
75%>
75-90%
90-95%
95%<

eFigure 5. Task Performance Results Over Time at the Age 12-18 Months

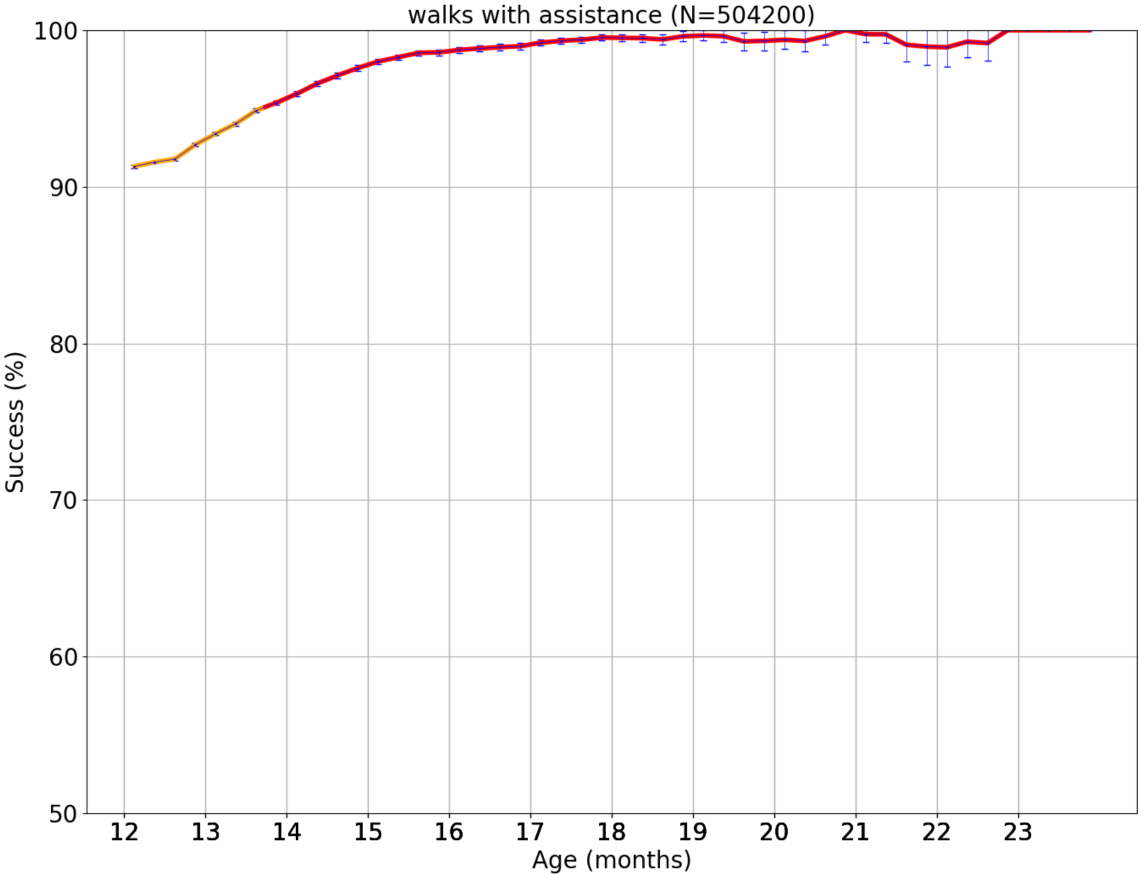

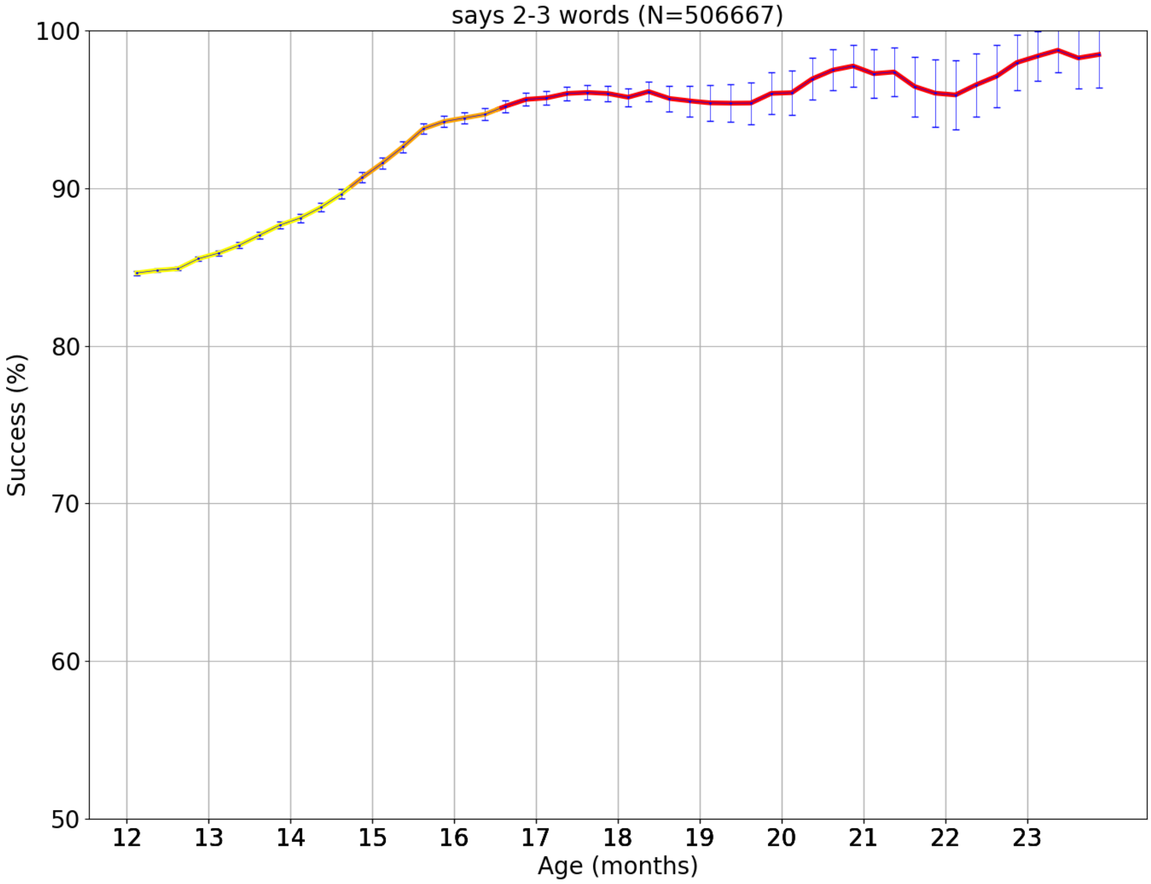

© 2022 Sudry T et al. *JAMA Network Open.*

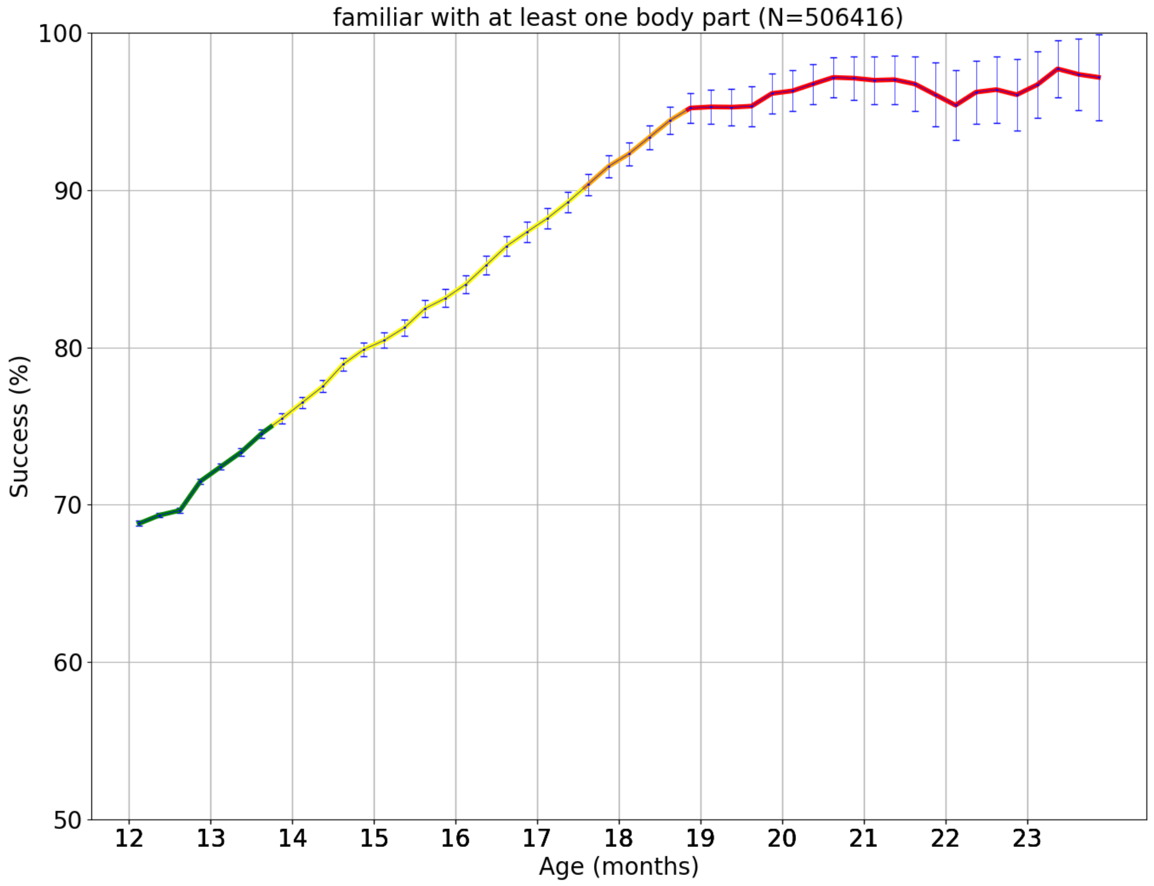

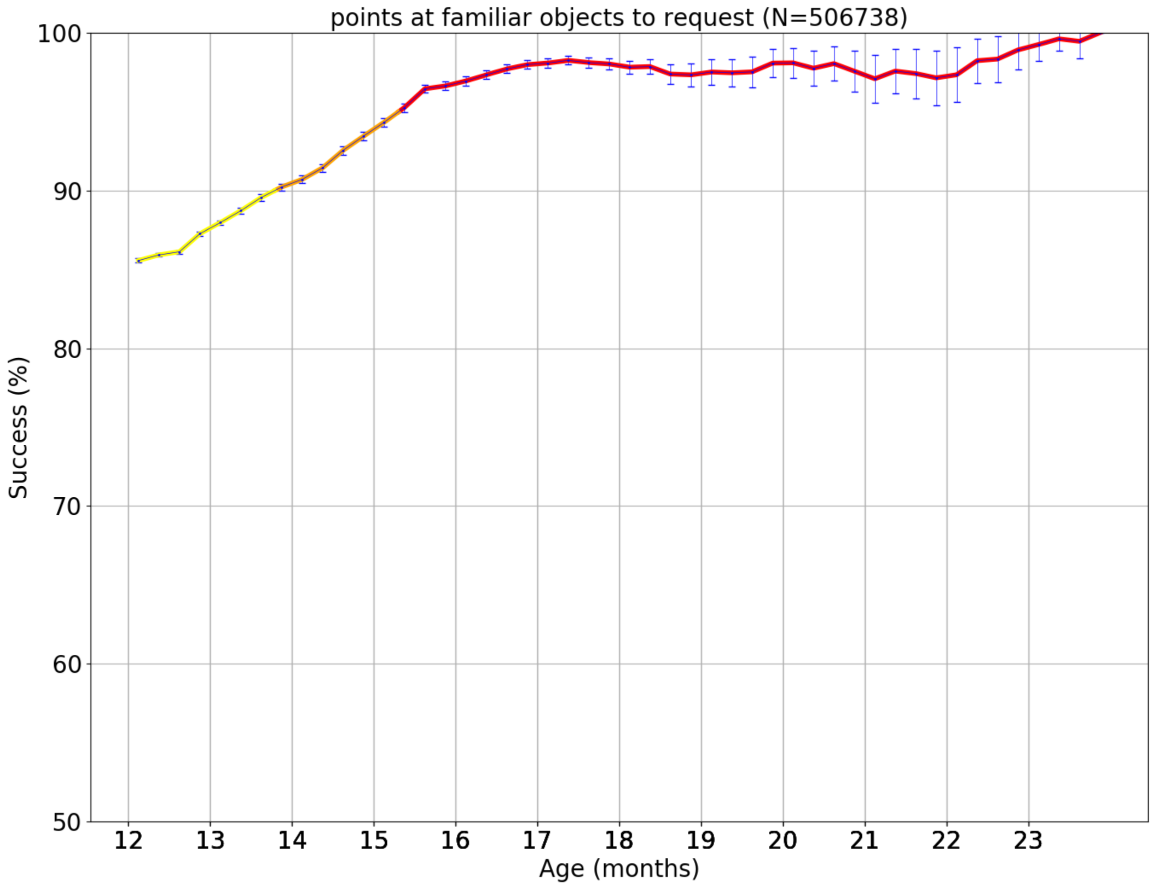

© 2022 Sudry T et al. *JAMA Network Open.*

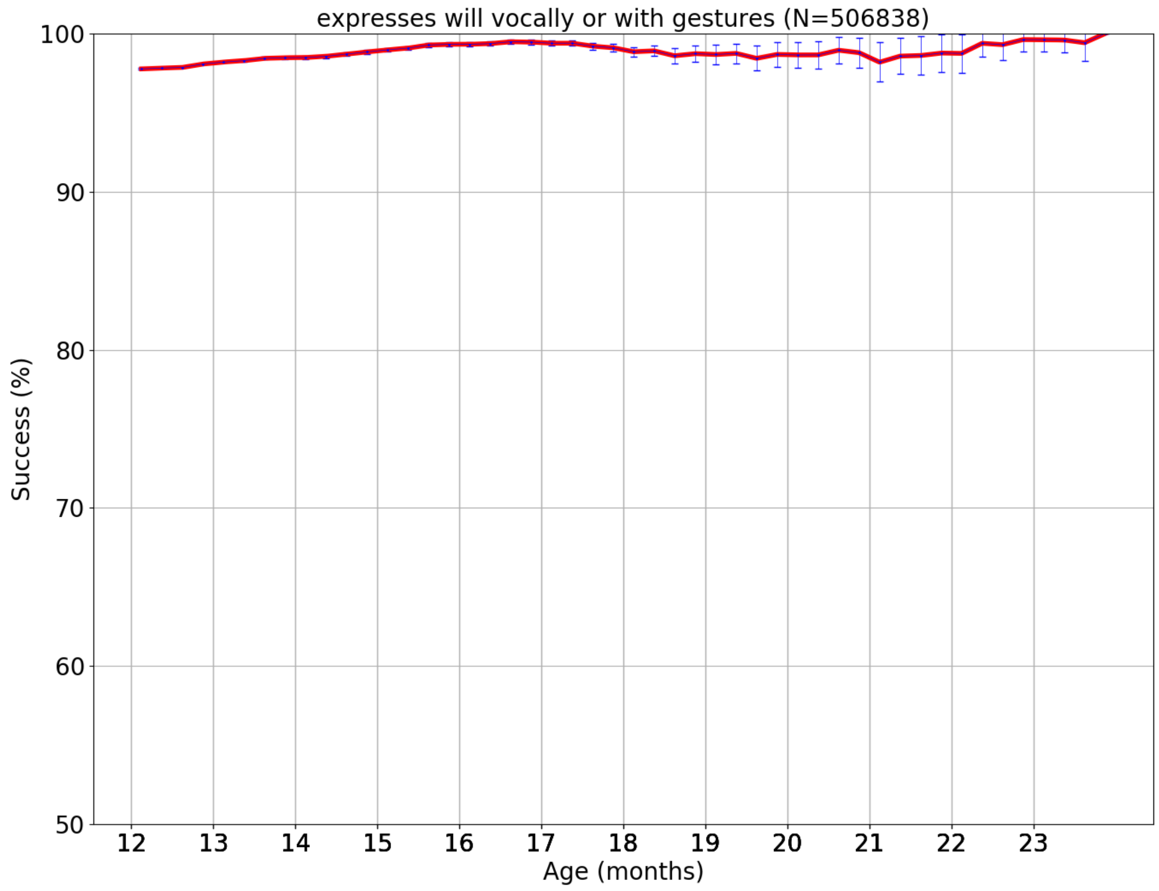

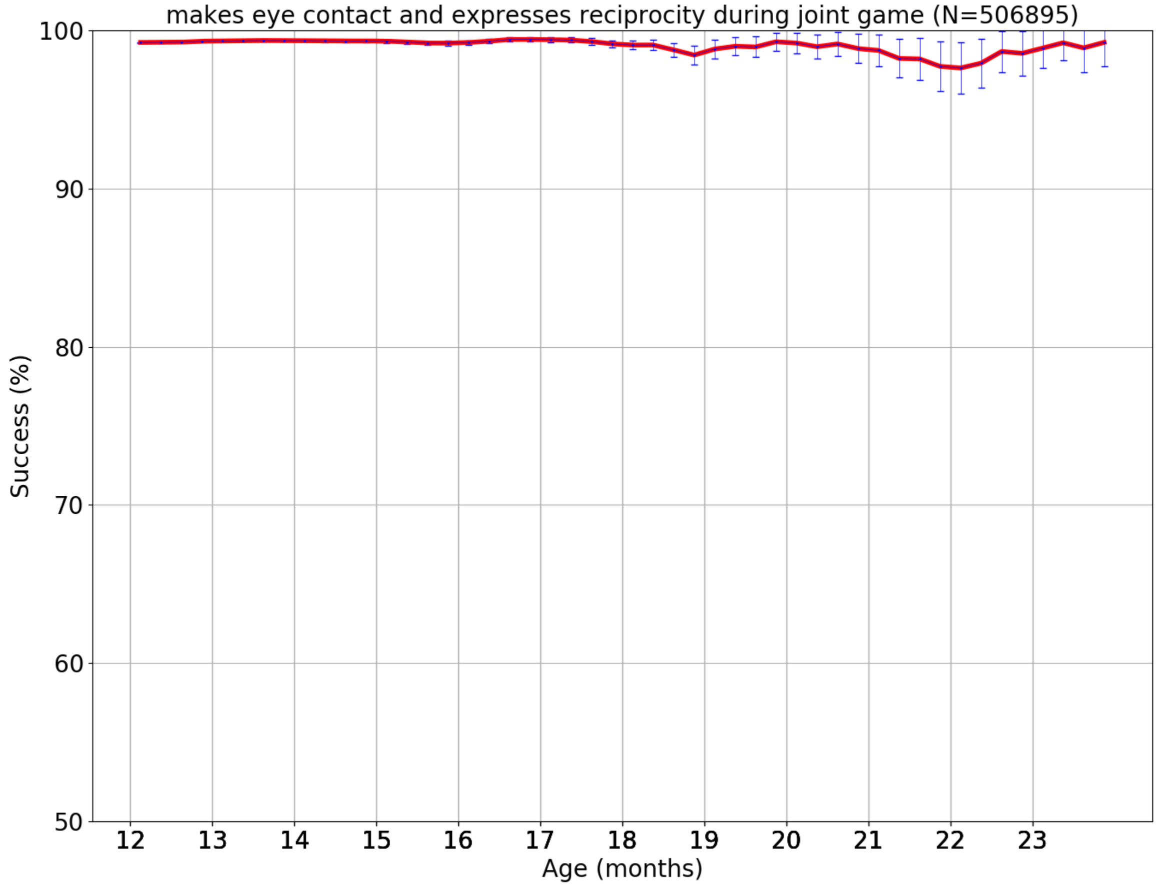

eFigure 5: The success rates over time of the different milestones evaluated between the ages 12-18 months. The achievement rates of < 75%, 75-90%, 90-95% and > 95% of children are indicated by the colors green, yellow, orange and red, respectively. The error bars are 95% confidence intervals of a binomial proportion, where larger bars indicate smaller sample size.

© 2022 Sudry T et al. *JAMA Network Open.*

Achievement rate
75%>
75-90%
90-95%
95%<

eFigure 6. Task Performance Results Over Time at the Age 18-24 Months

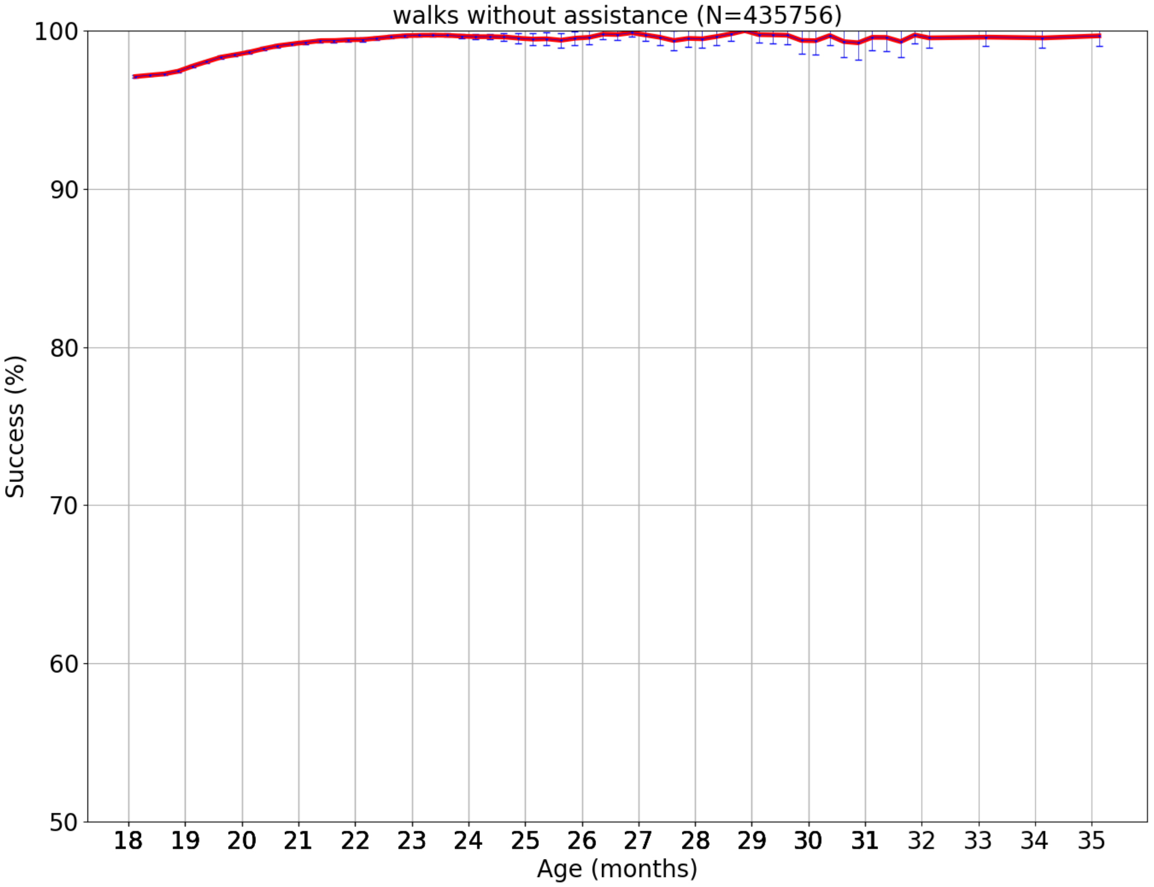

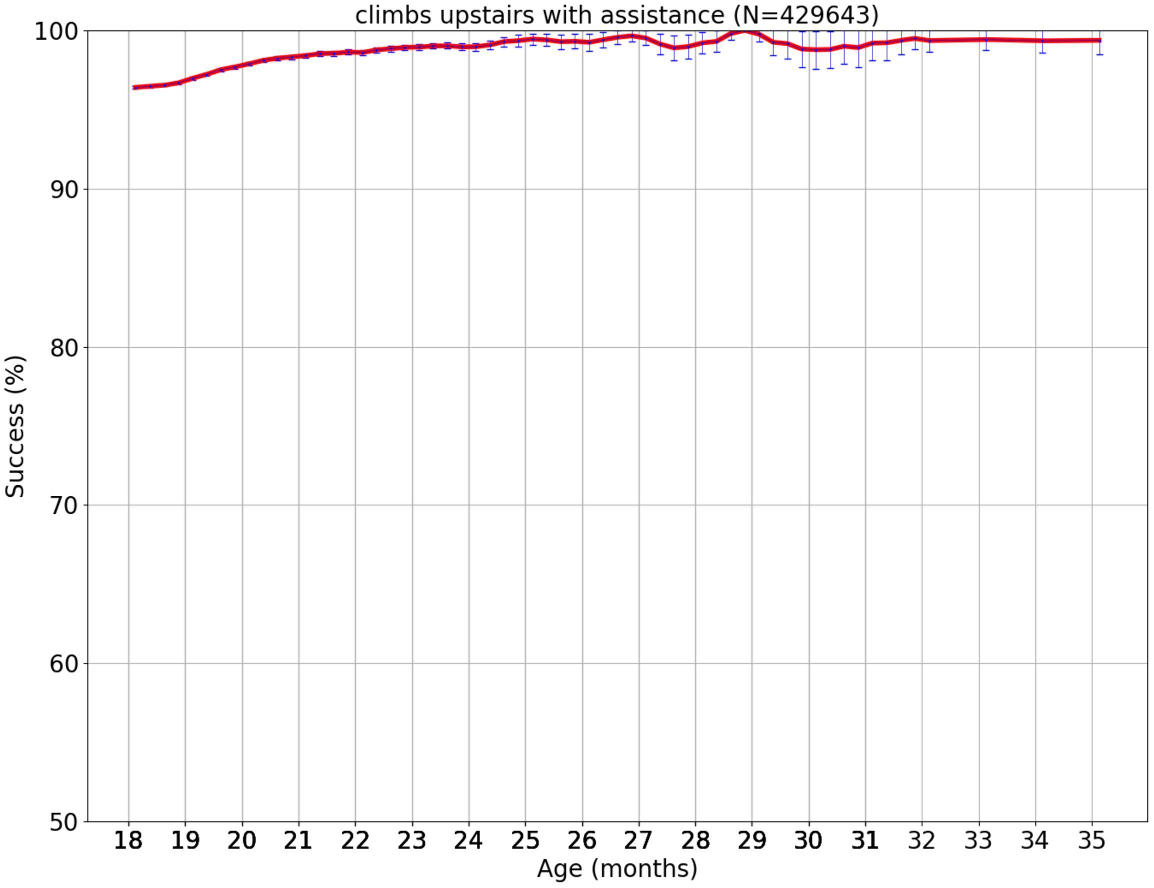

© 2022 Sudry T et al. *JAMA Network Open.*

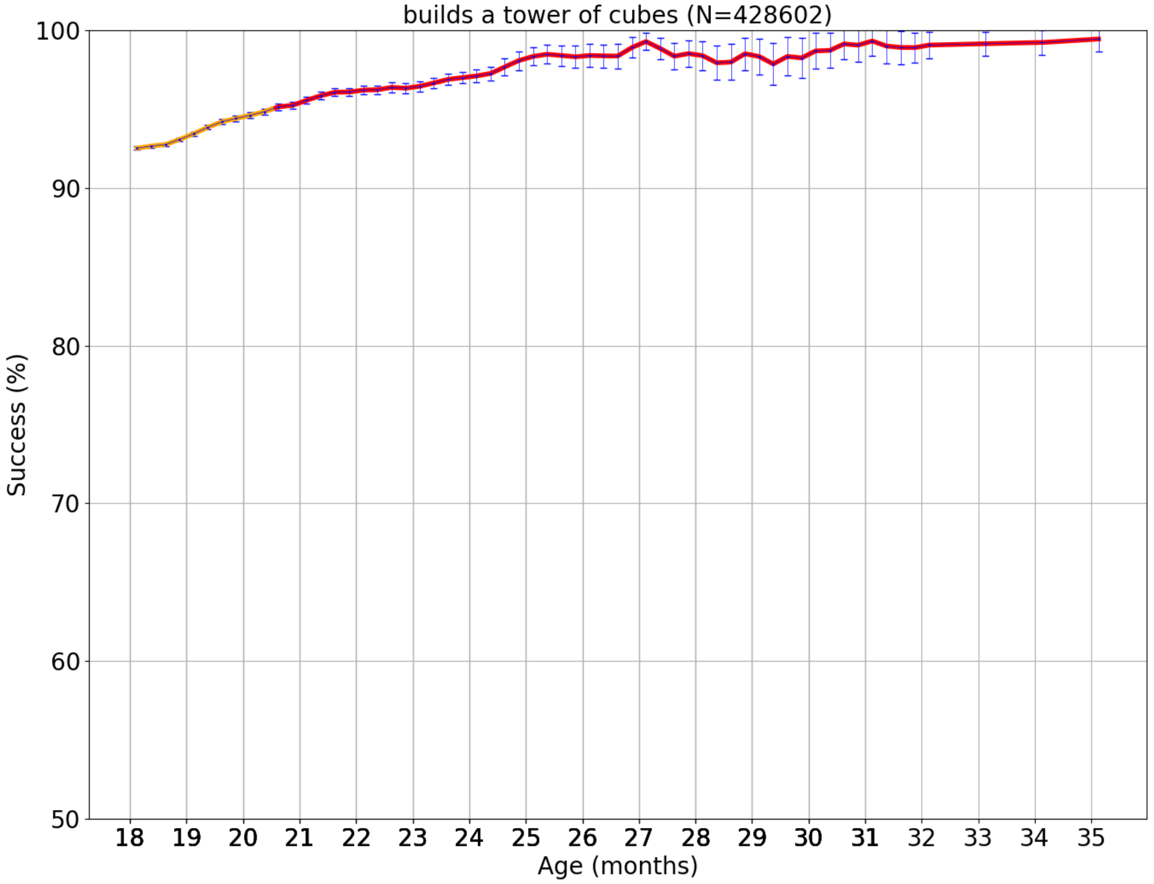

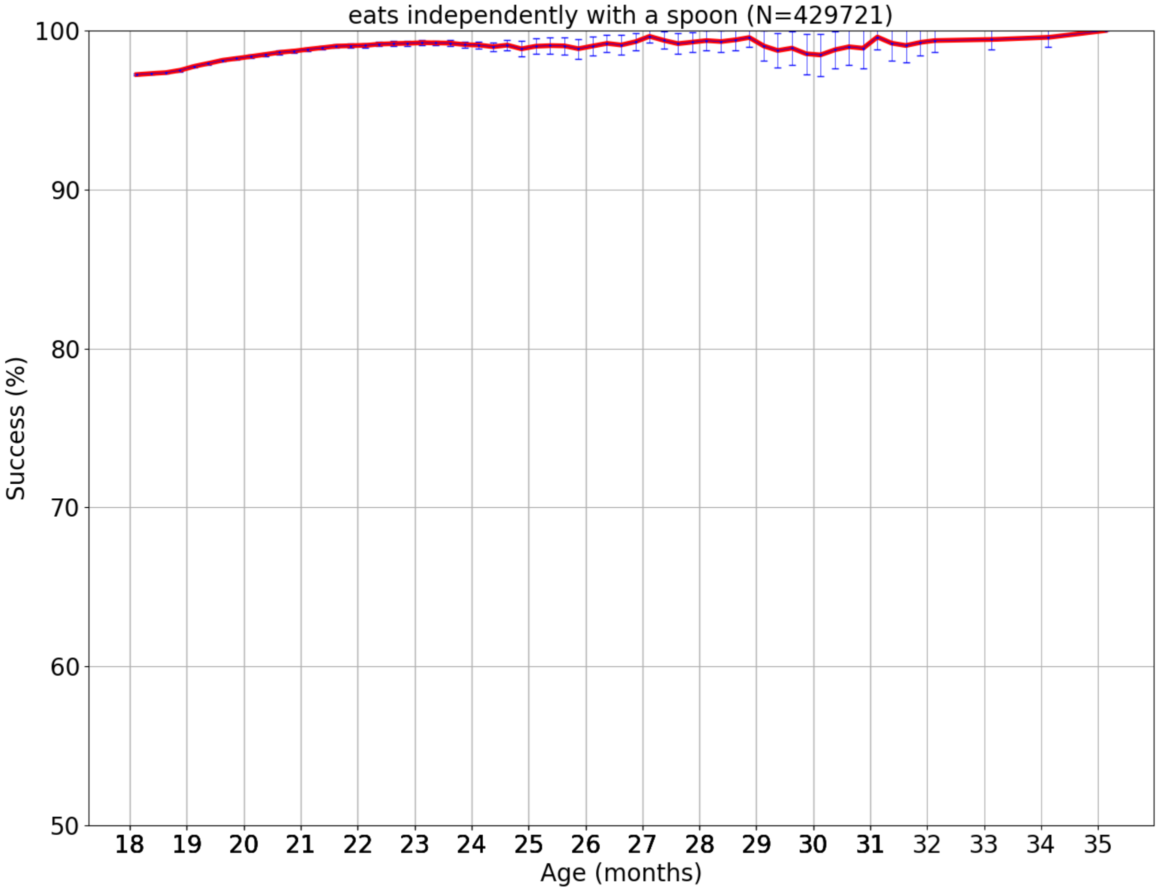

© 2022 Sudry T et al. *JAMA Network Open.*

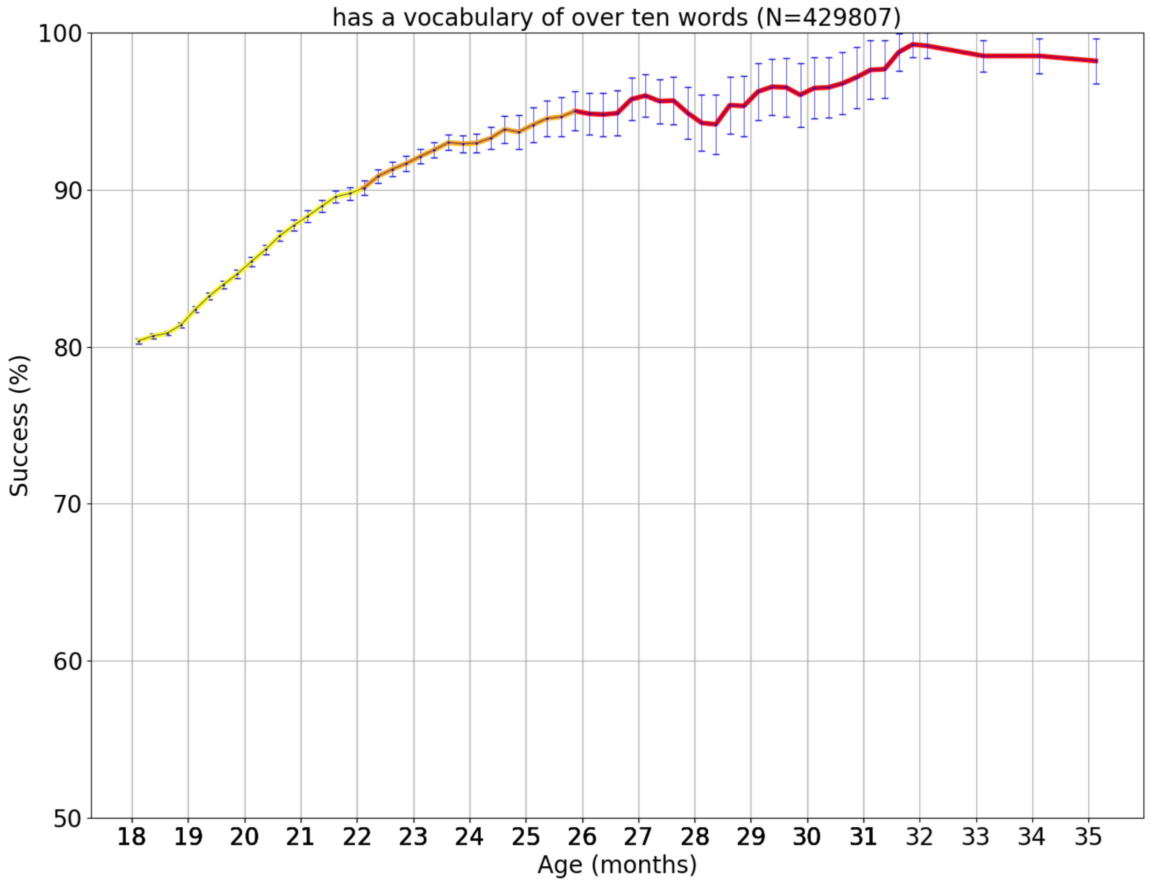

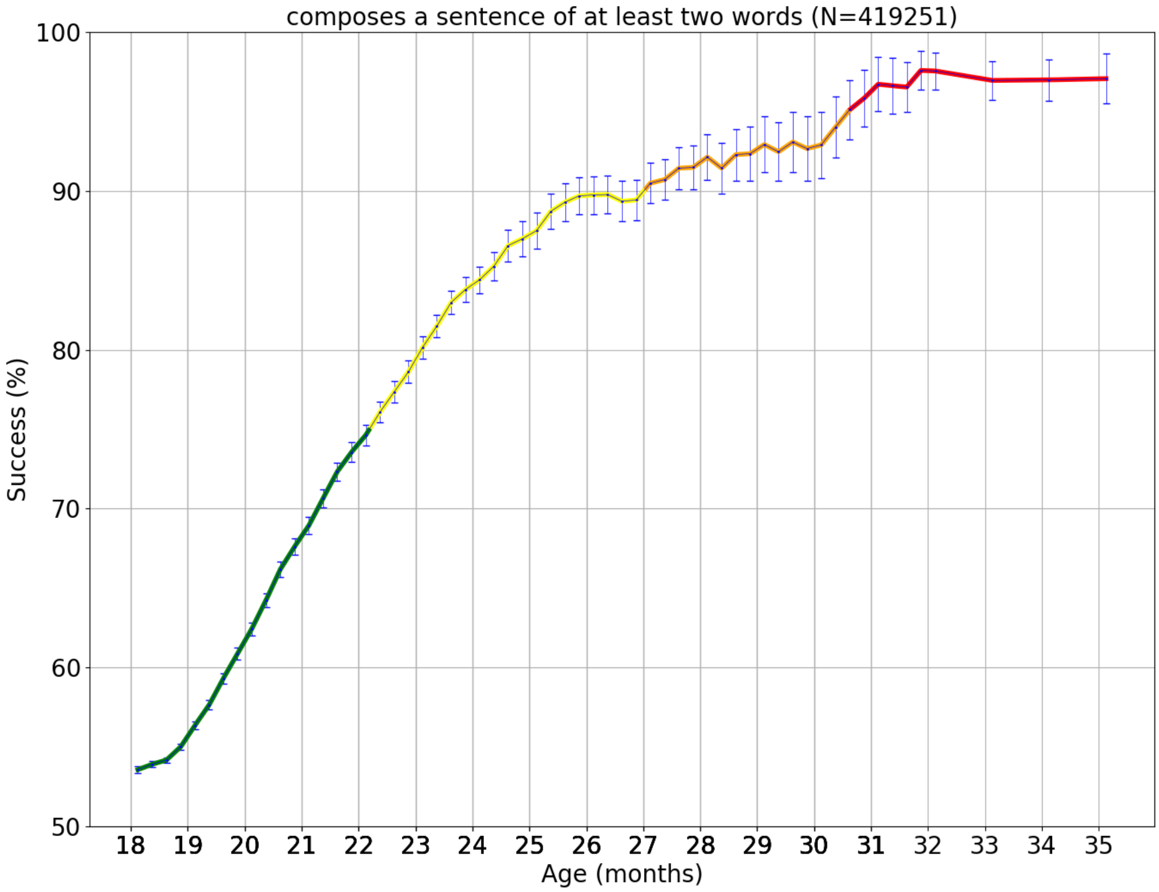

© 2022 Sudry T et al. *JAMA Network Open.*

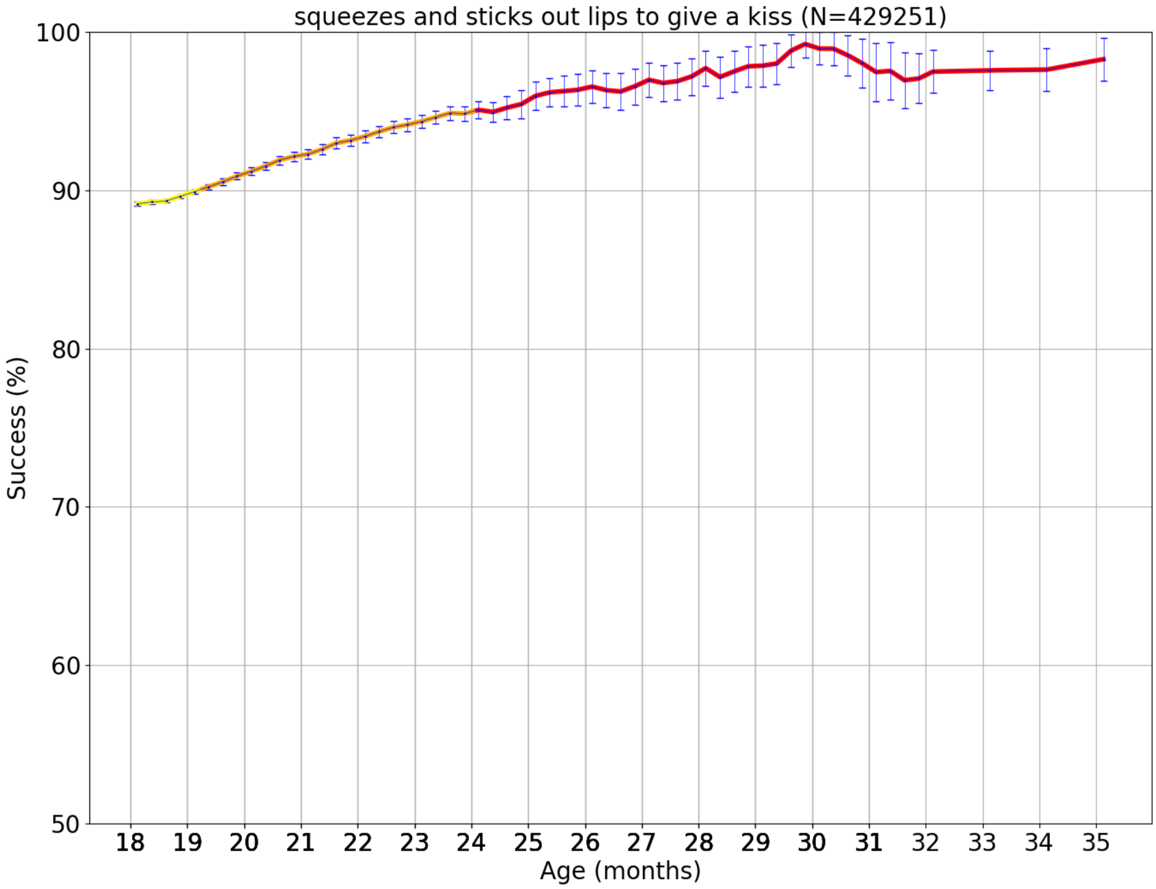

eFigure 6: The success rates over time of the different milestones evaluated between the ages 18-24 months. The achievement rates of < 75%, 75-90%, 90-95% and > 95% of children are indicated by the colors green, yellow, orange and red, respectively. The error bars are 95% confidence intervals of a binomial proportion, where larger bars indicate smaller sample size.

© 2022 Sudry T et al. *JAMA Network Open.*

eFigure 7. Task Performance Results Over Time at the Age 2-3 Years (24-36 Months)

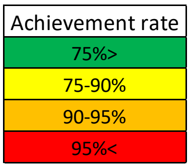

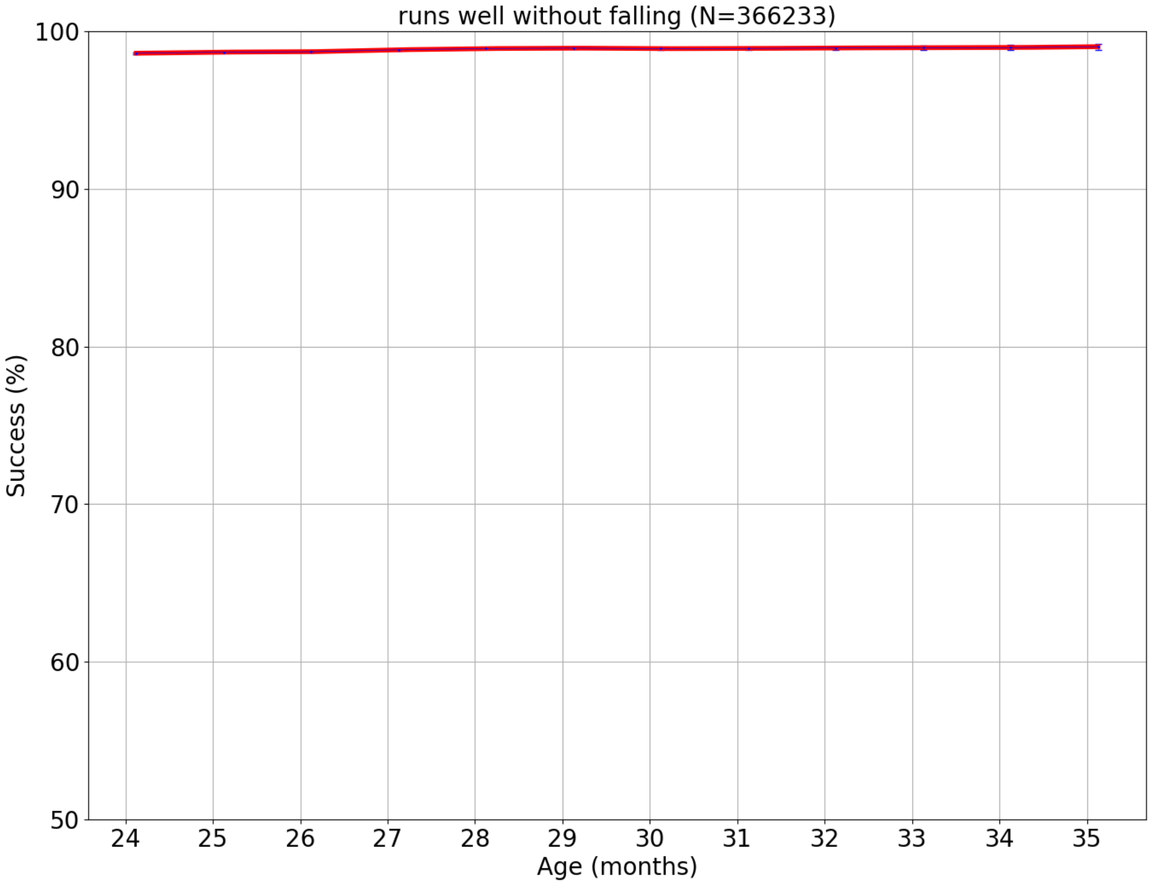

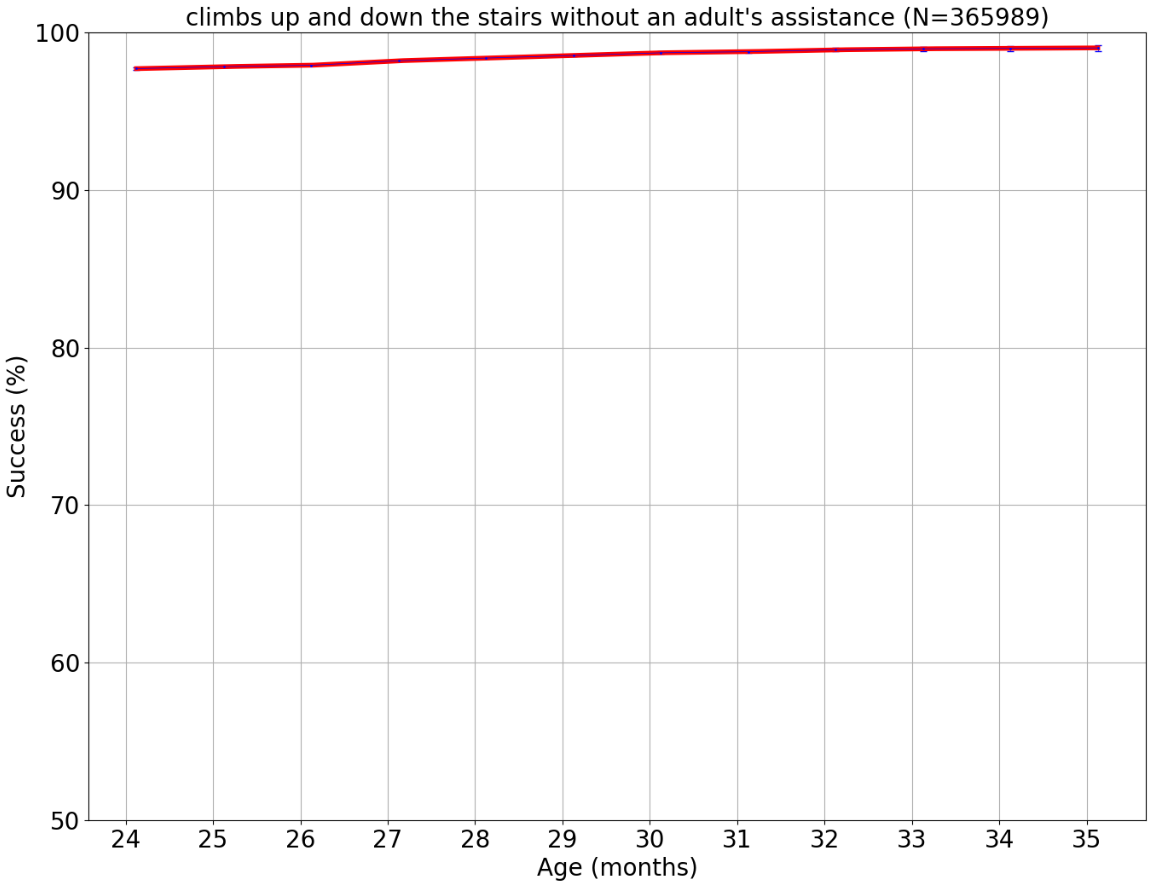

© 2022 Sudry T et al. *JAMA Network Open.*

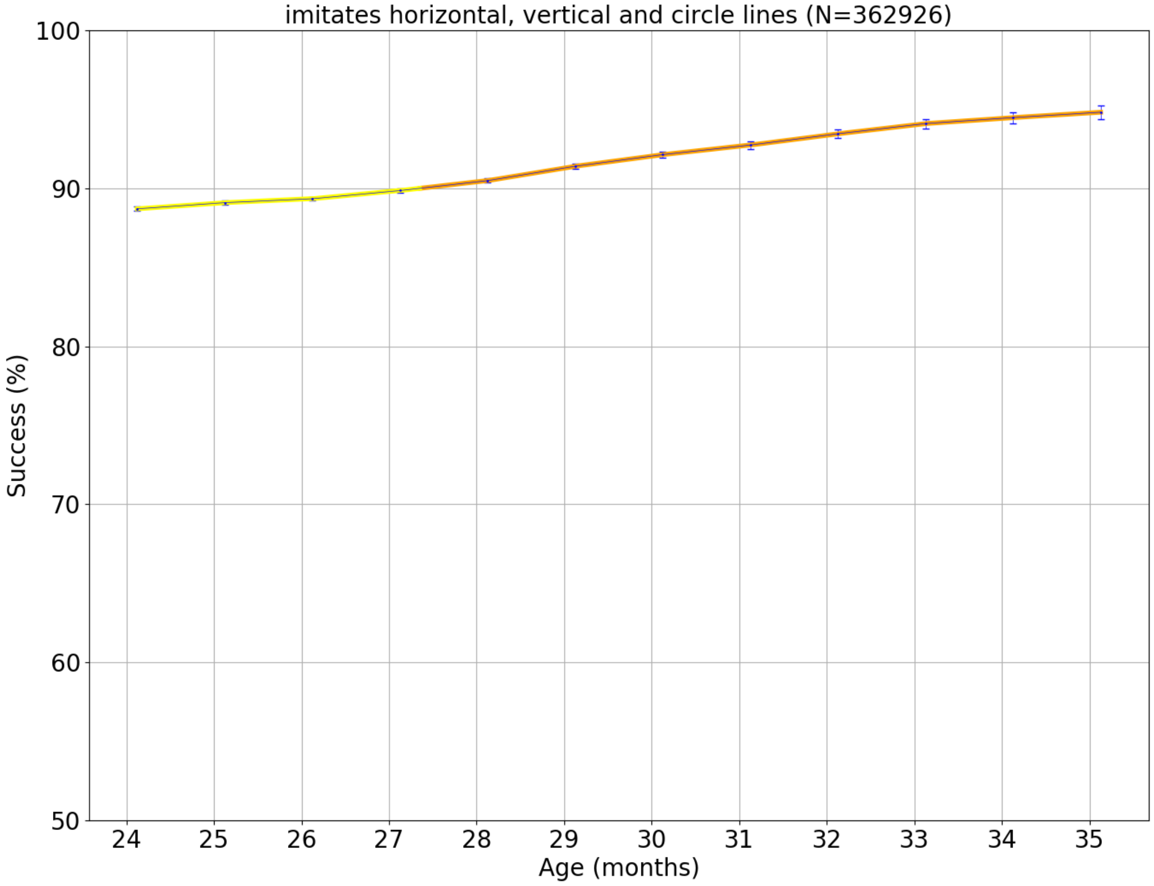

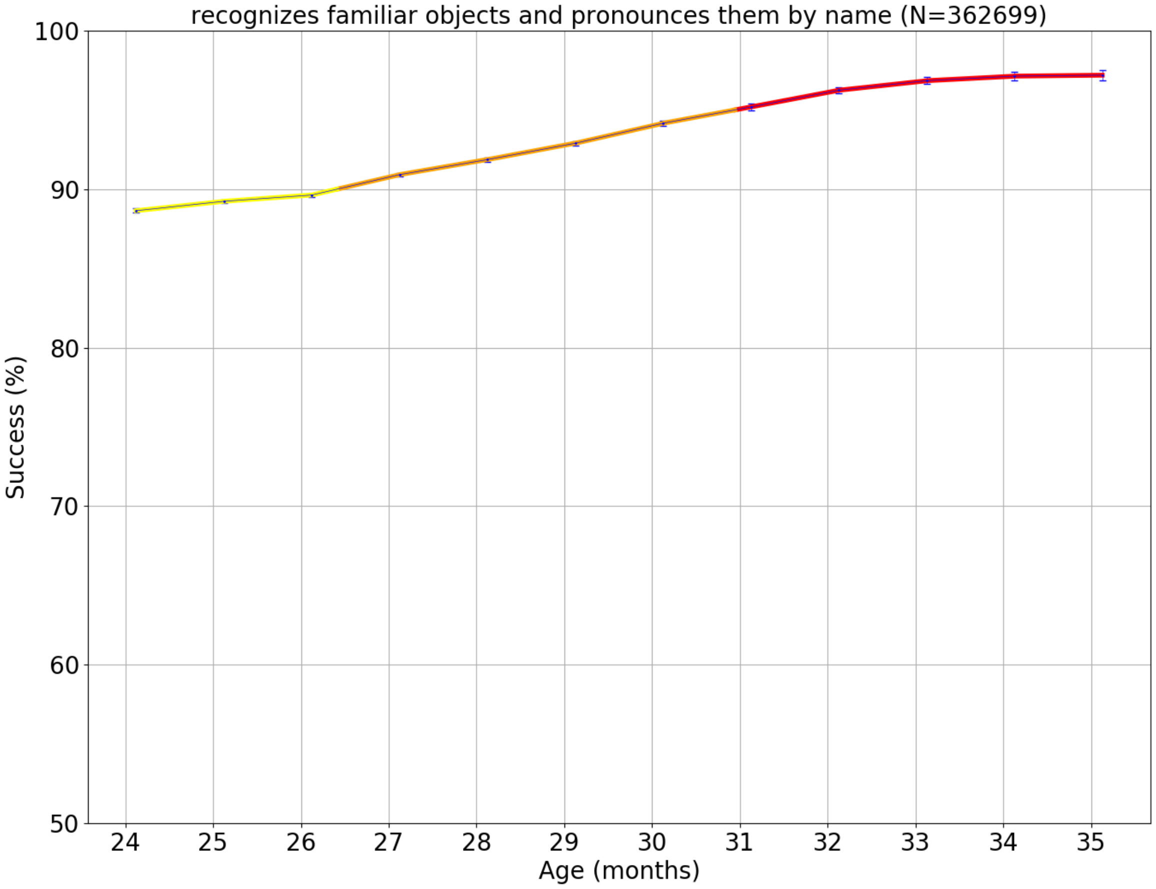

© 2022 Sudry T et al. *JAMA Network Open.*

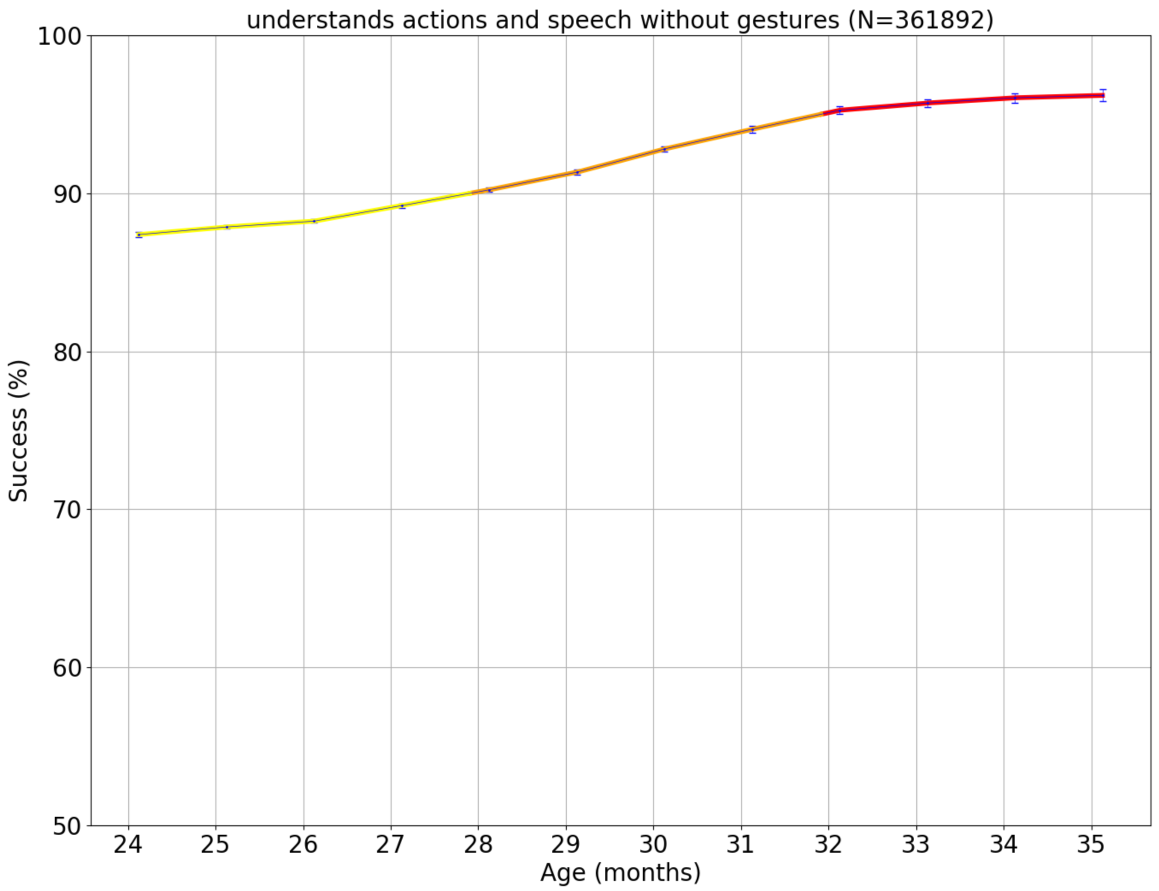

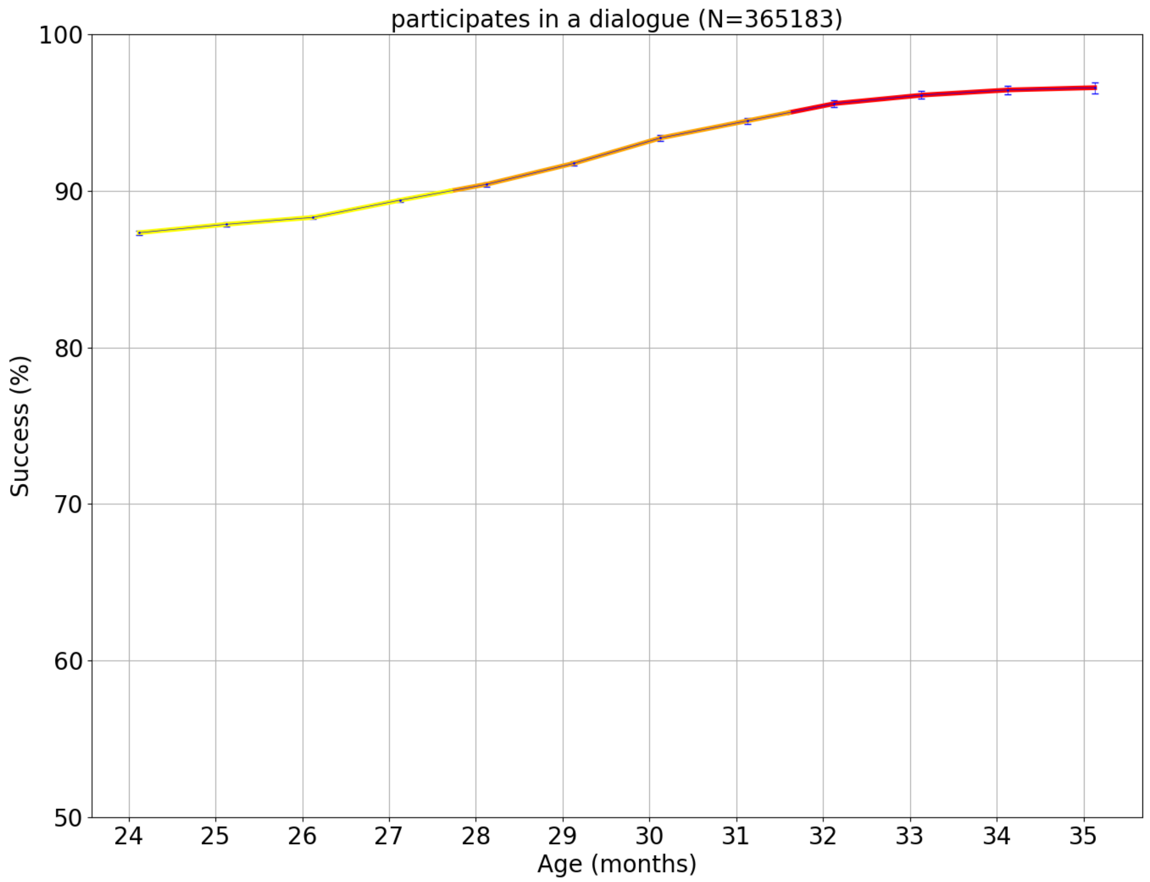

© 2022 Sudry T et al. *JAMA Network Open.*

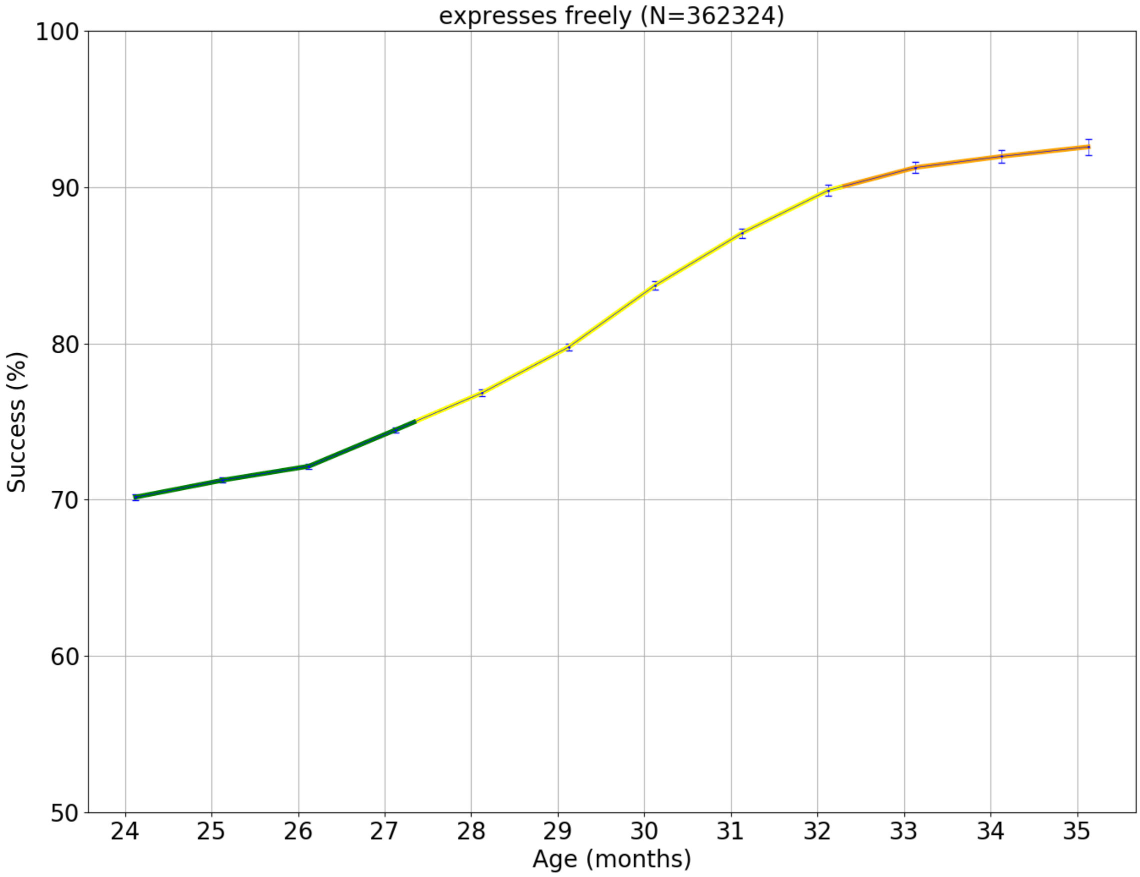

eFigure 7: The success rates over time of the different milestones evaluated between the ages 24-36 months. The achievement rates of < 75%, 75-90%, 90-95% and > 95% of children are indicated by the colors green, yellow, orange and red, respectively. The error bars are 95% confidence intervals of a binomial proportion, where larger bars indicate smaller sample size.

© 2022 Sudry T et al. *JAMA Network Open.*

Achievement rate
75%>
75-90%
90-95%
95%<

eFigure 8. Task Performance Results Over Time at the Age 3-4 Years (36-48 Months)

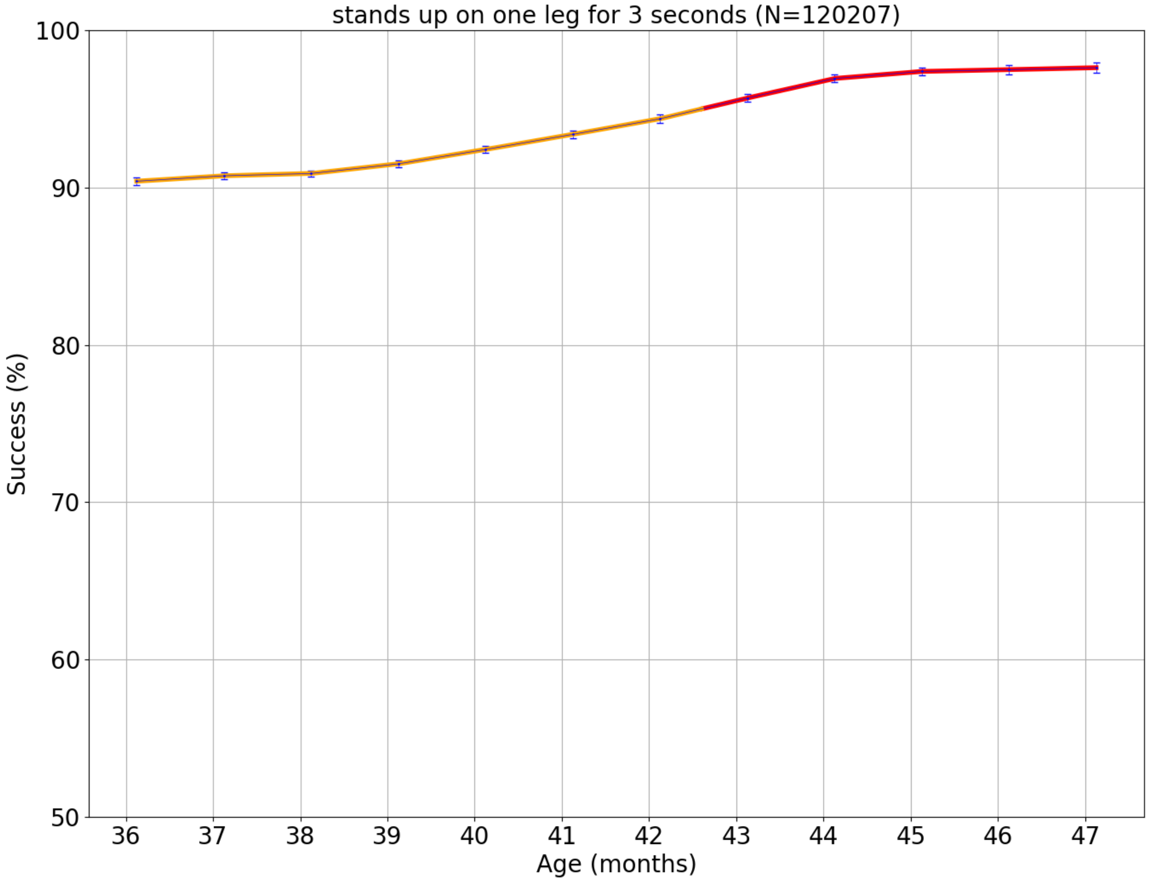

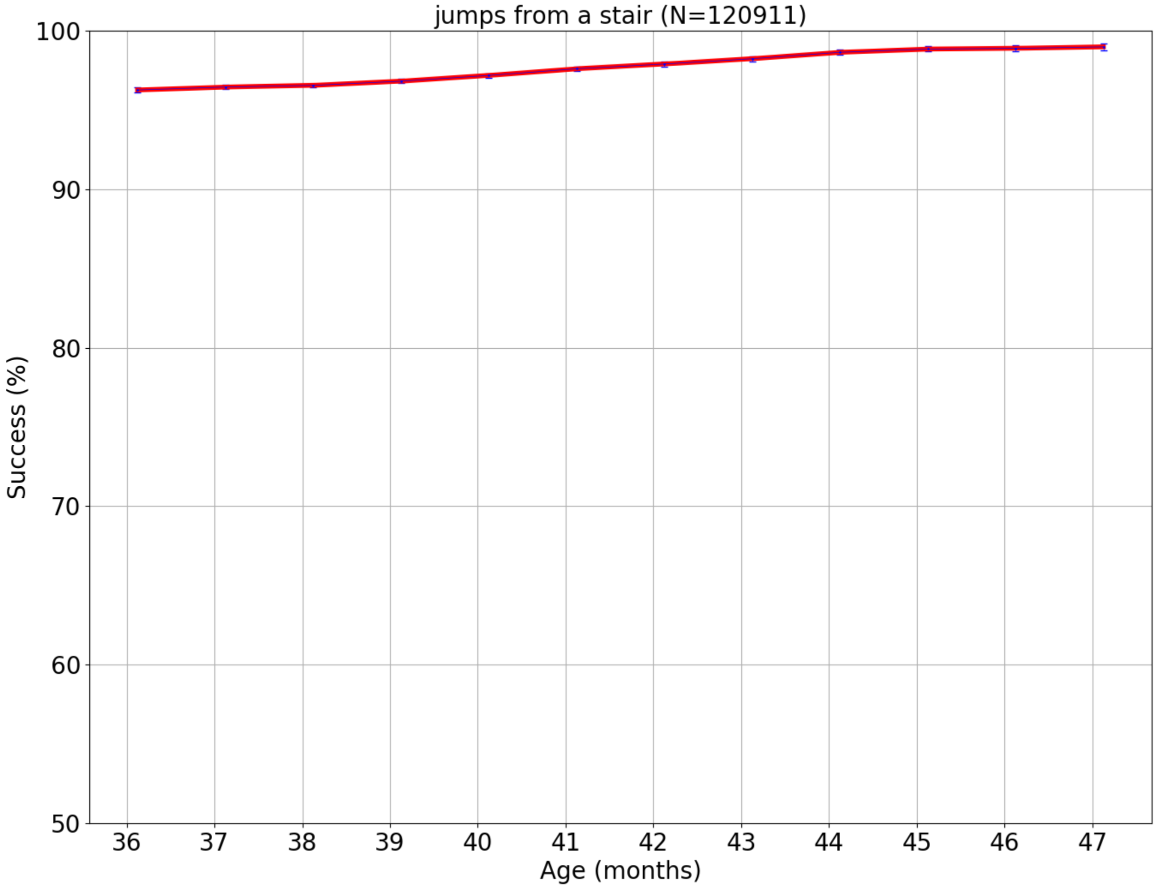

© 2022 Sudry T et al. *JAMA Network Open.*

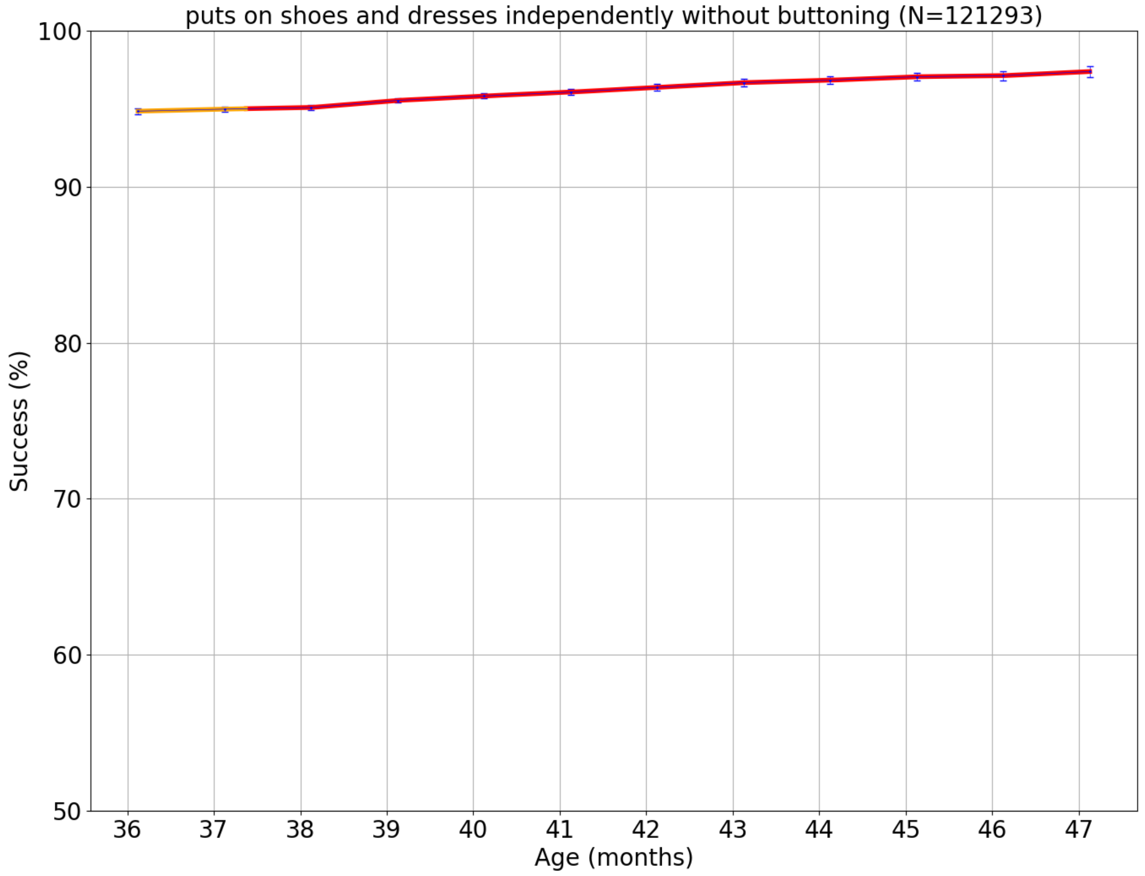

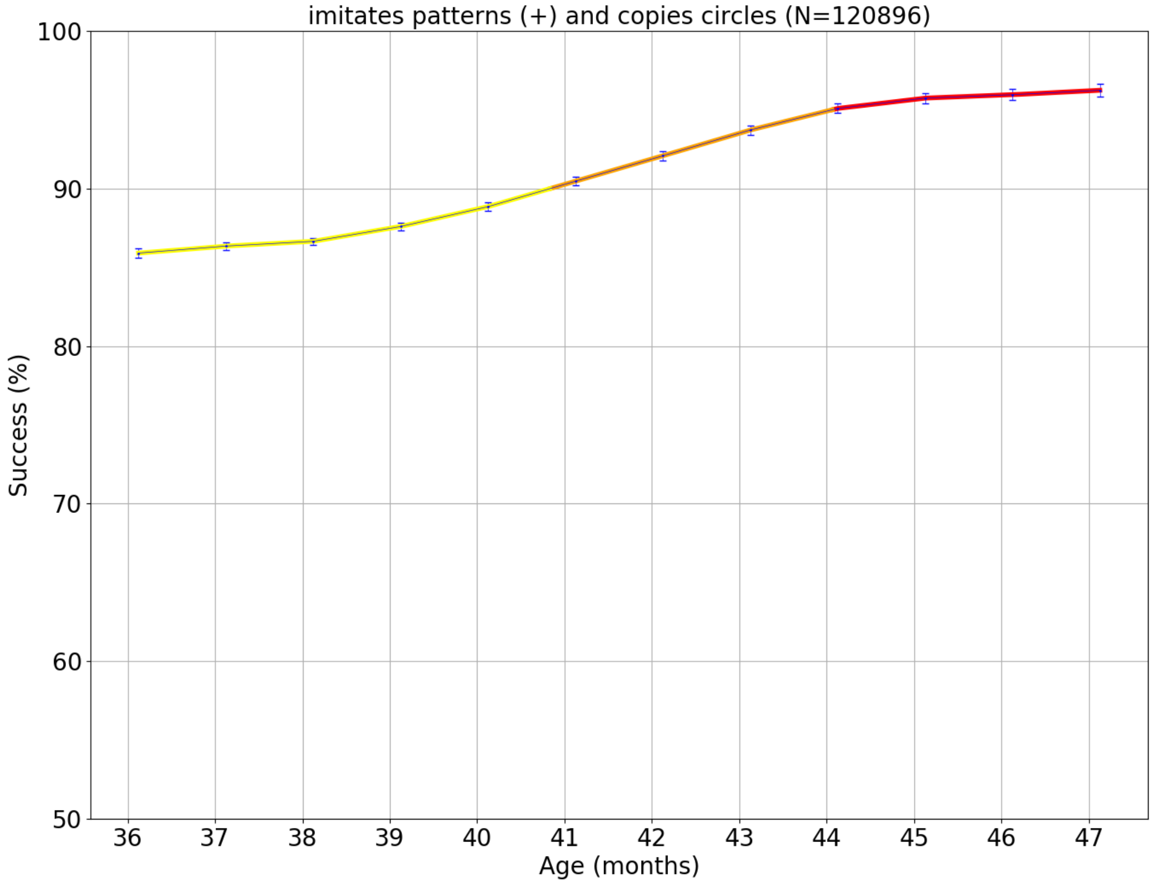

© 2022 Sudry T et al. *JAMA Network Open.*

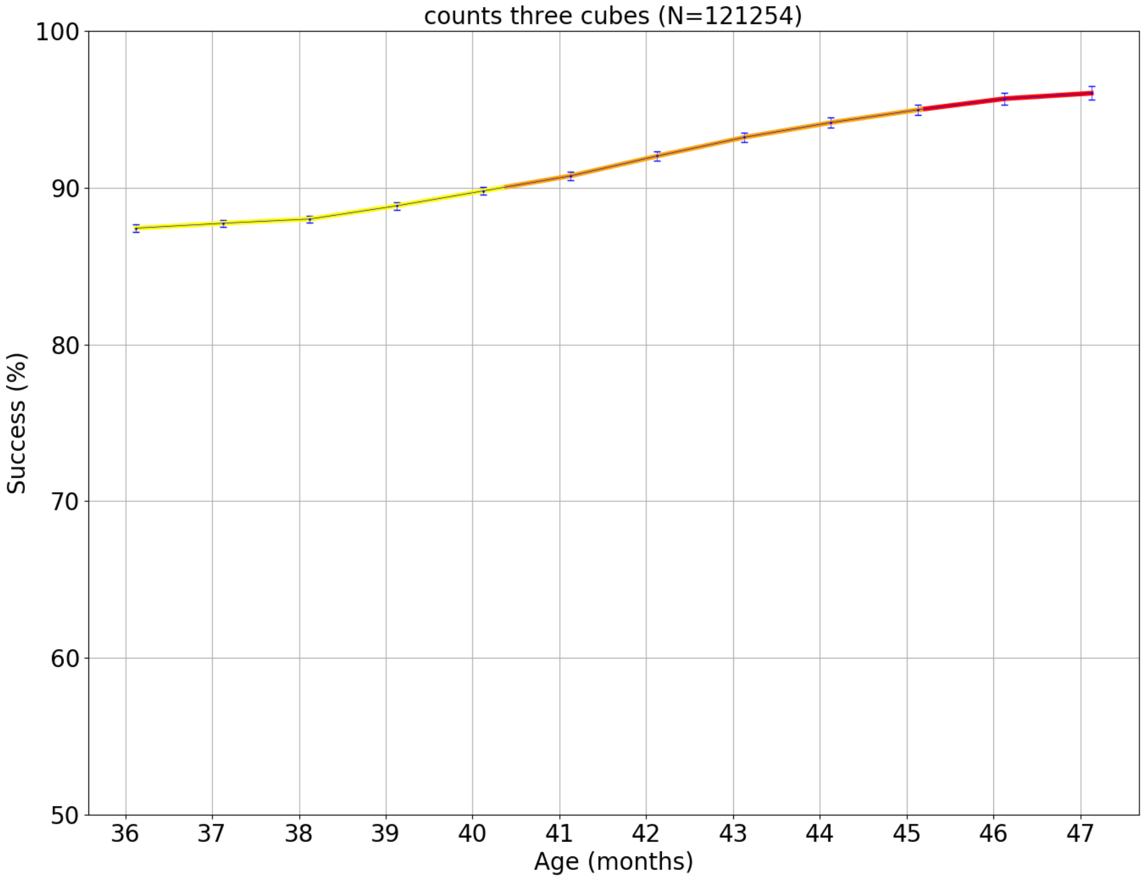

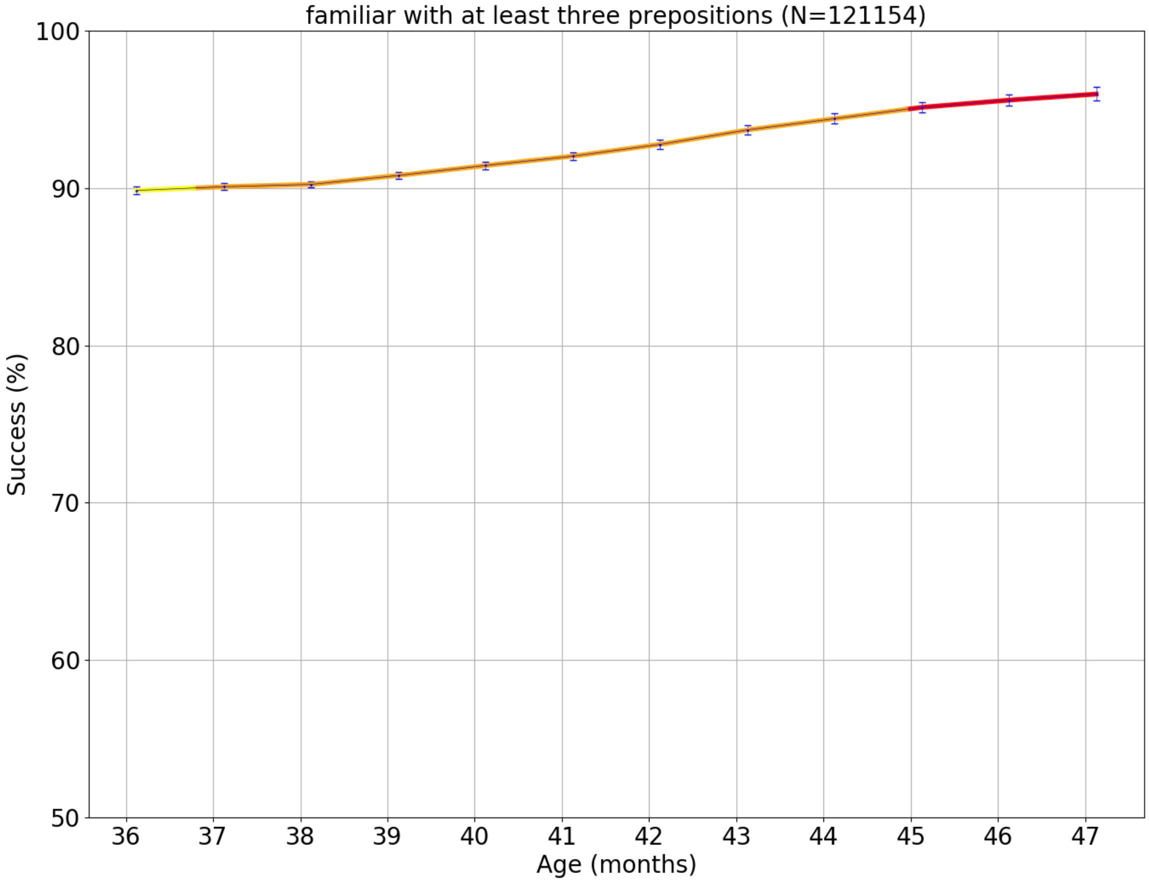

© 2022 Sudry T et al. *JAMA Network Open.*

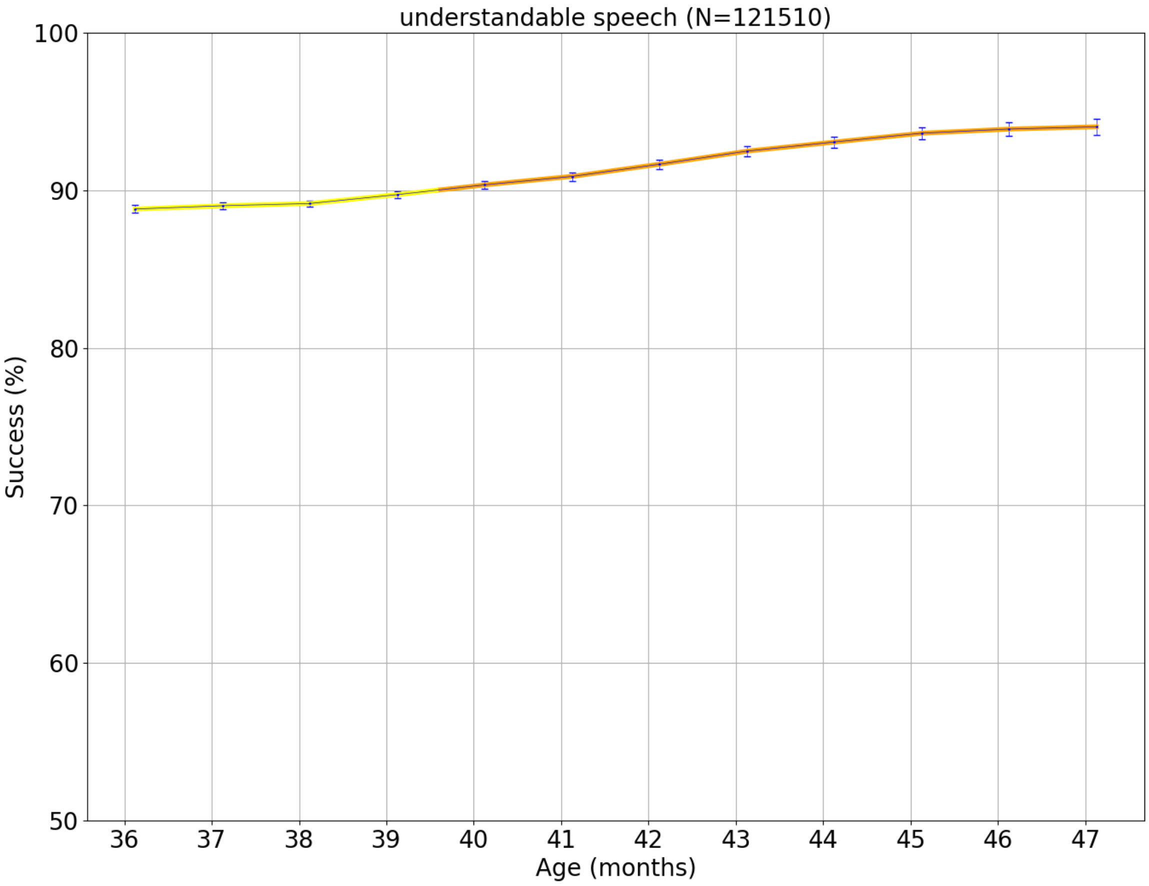

eFigure 8: The success rates over time of the different milestones evaluated between the ages 36-48 months. The achievement rates of < 75%, 75-90%, 90-95% and > 95% of children are indicated by the colors green, yellow, orange and red, respectively. The error bars are 95% confidence intervals of a binomial proportion, where larger bars indicate smaller sample size.

© 2022 Sudry T et al. *JAMA Network Open.*

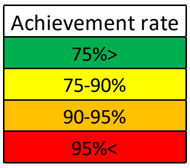

eFigure 9. Task Performance Results Over Time at the Age 4-6 Years (48-72 Months)

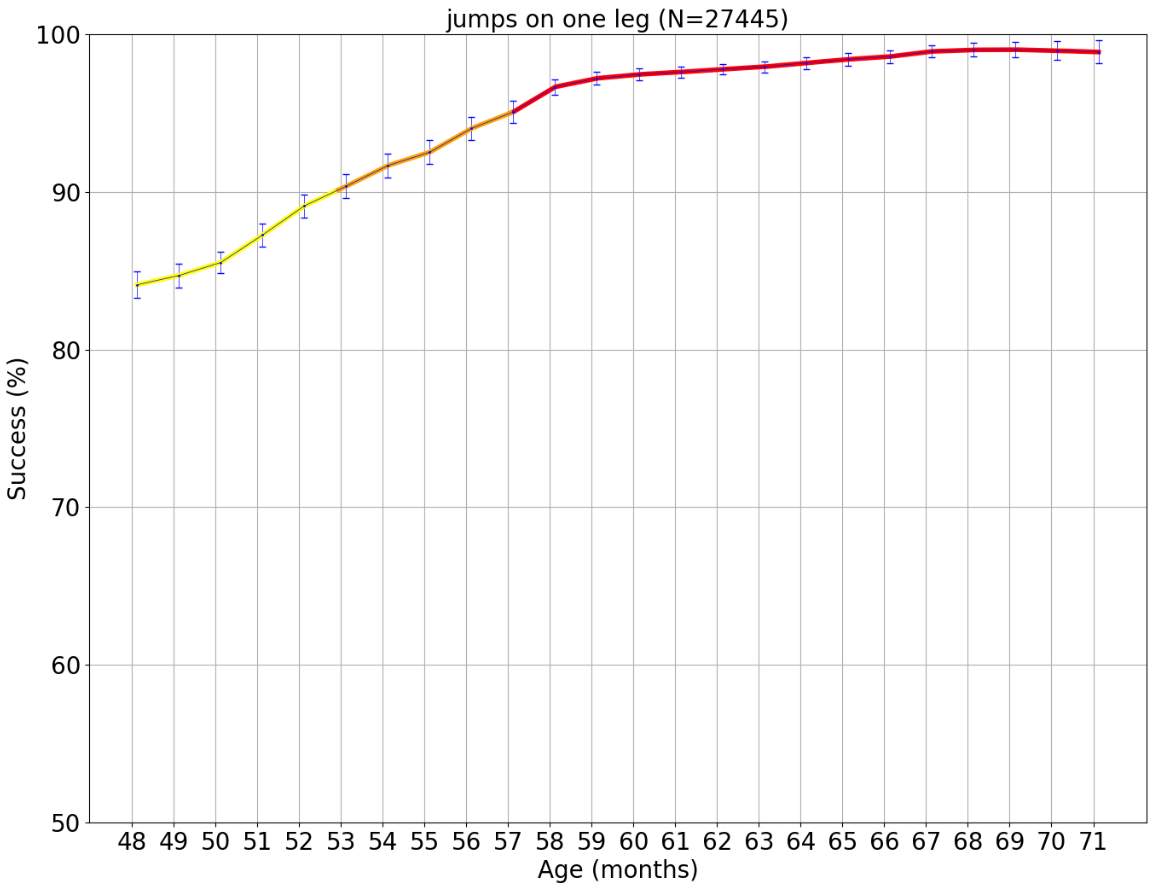

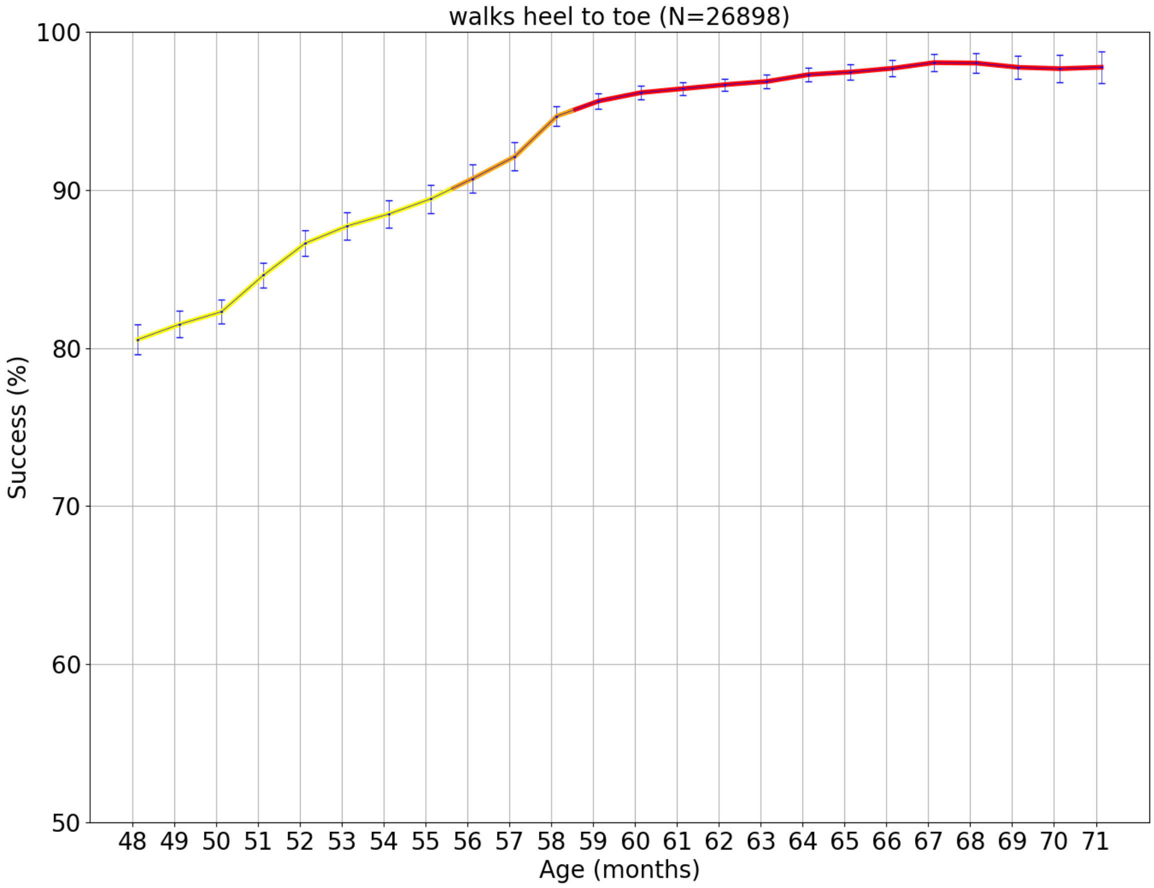

© 2022 Sudry T et al. *JAMA Network Open.*

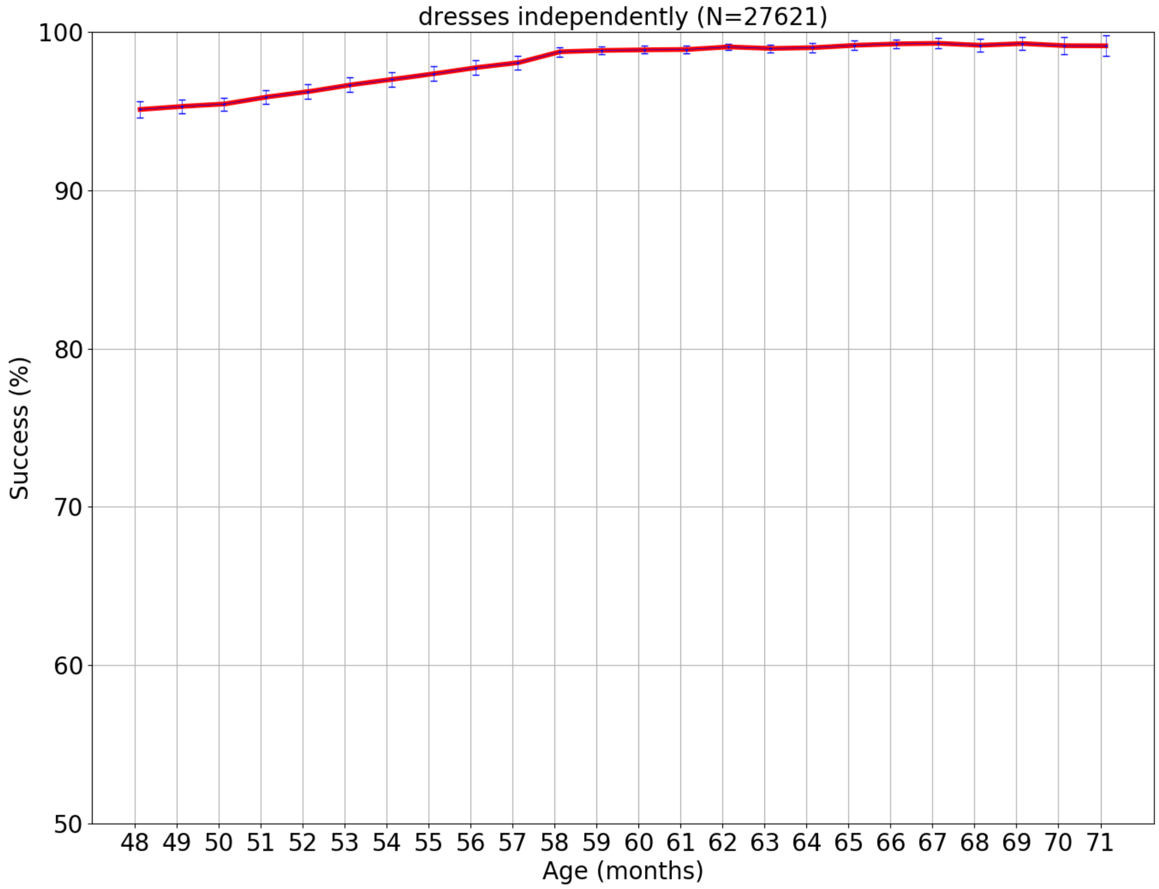

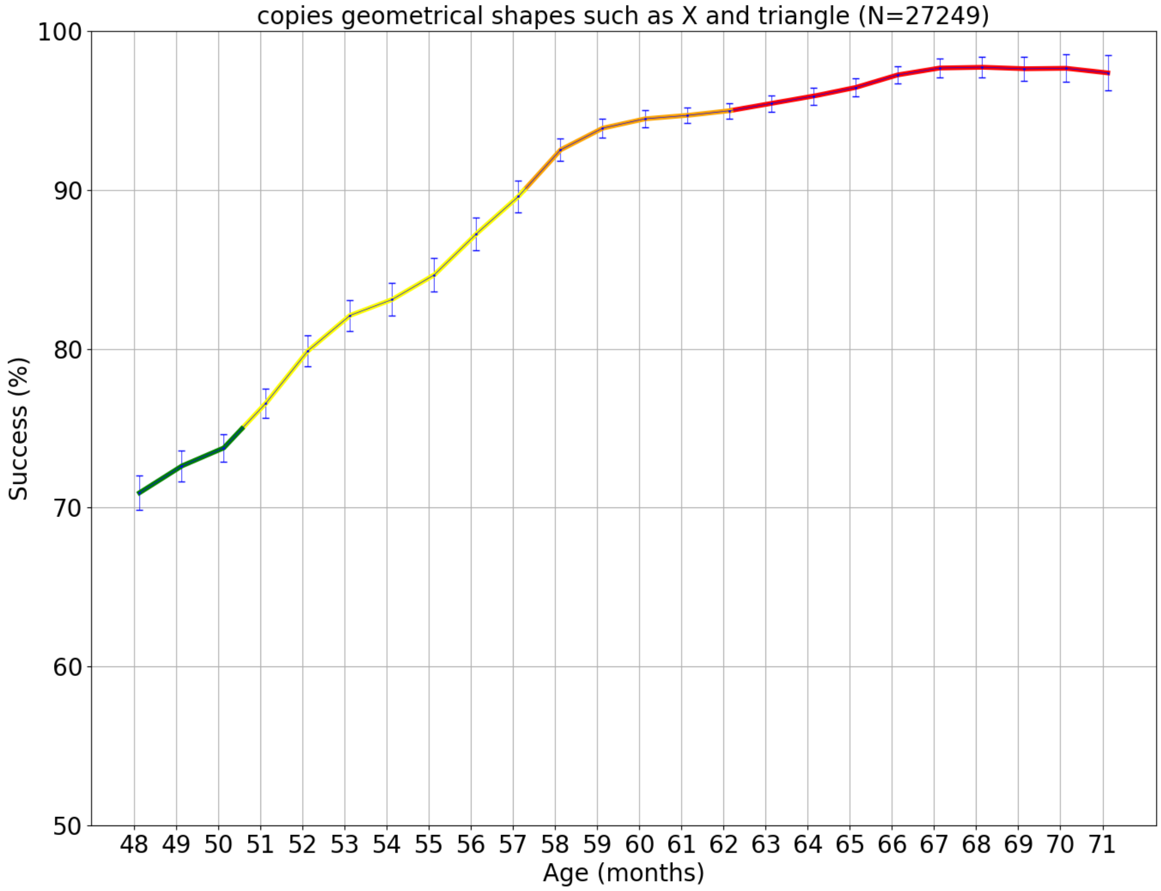

© 2022 Sudry T et al. *JAMA Network Open.*

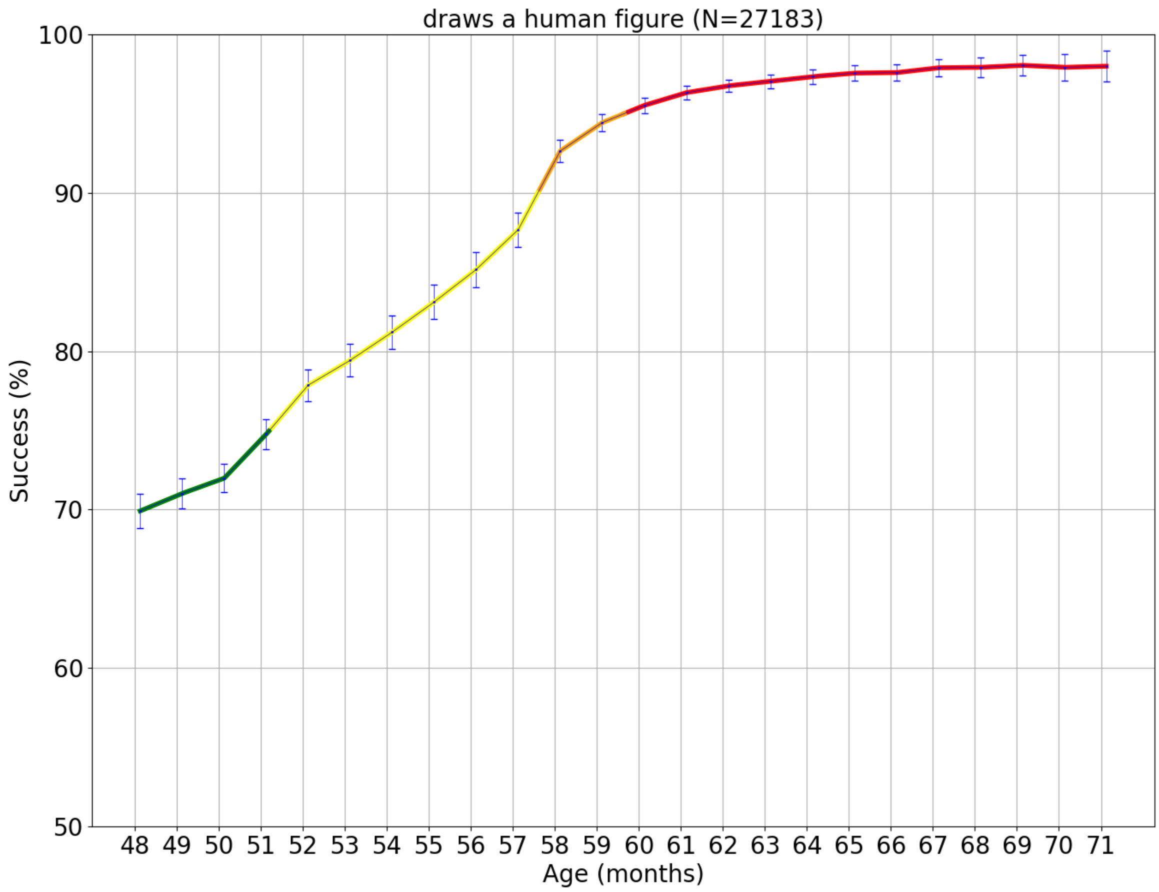

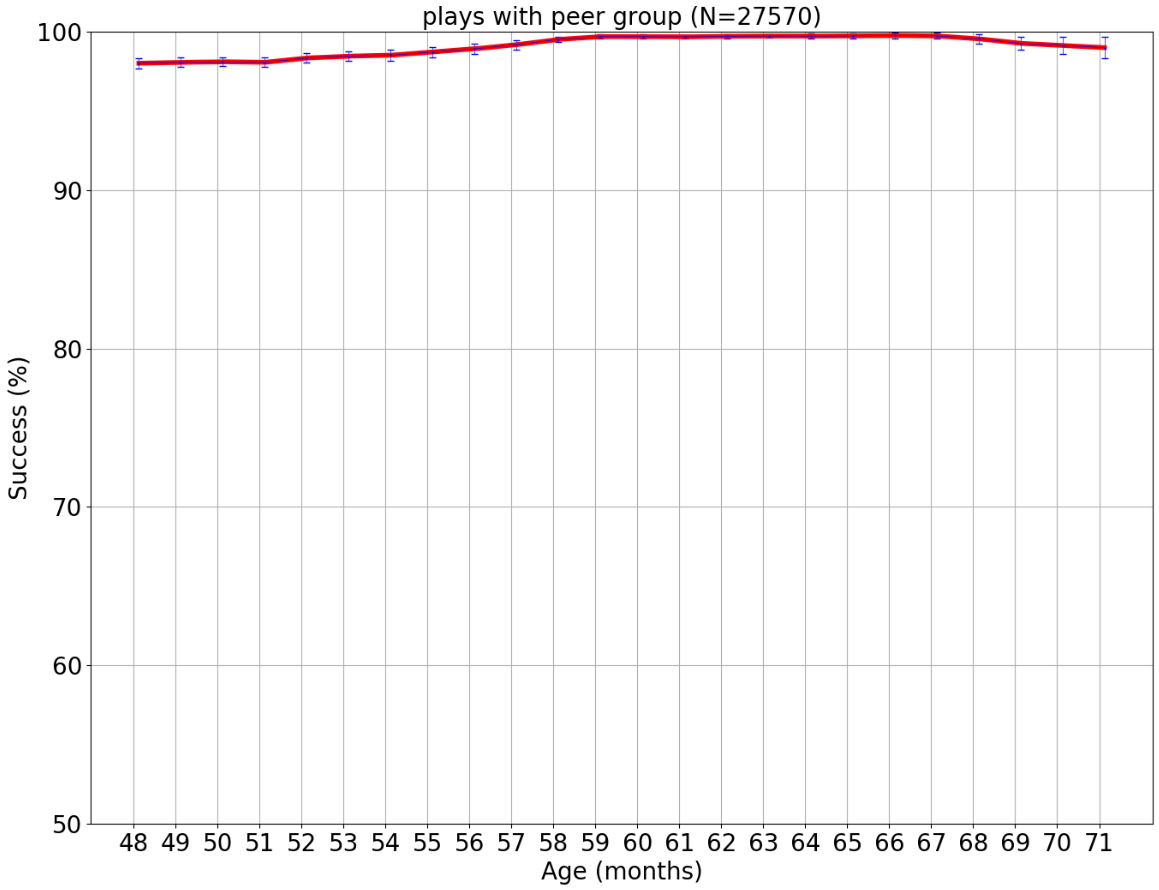

© 2022 Sudry T et al. *JAMA Network Open.*

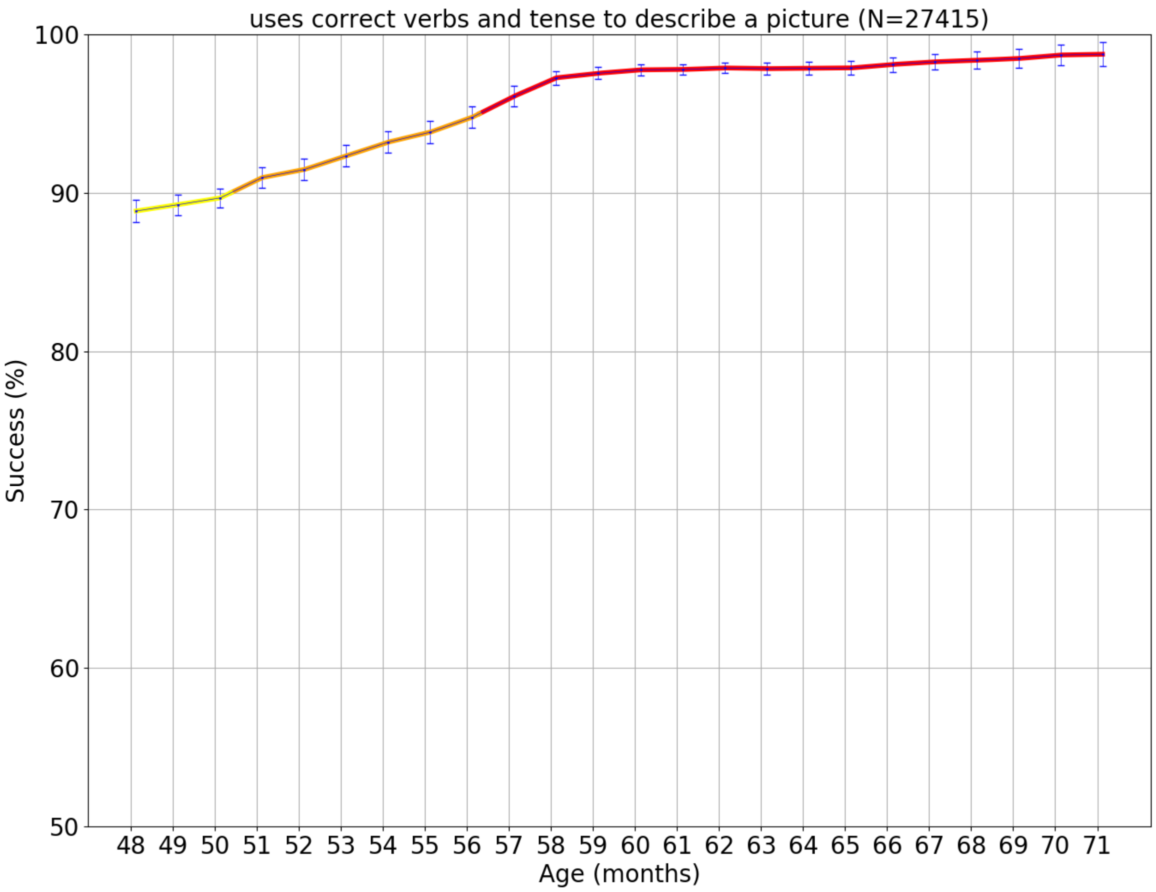

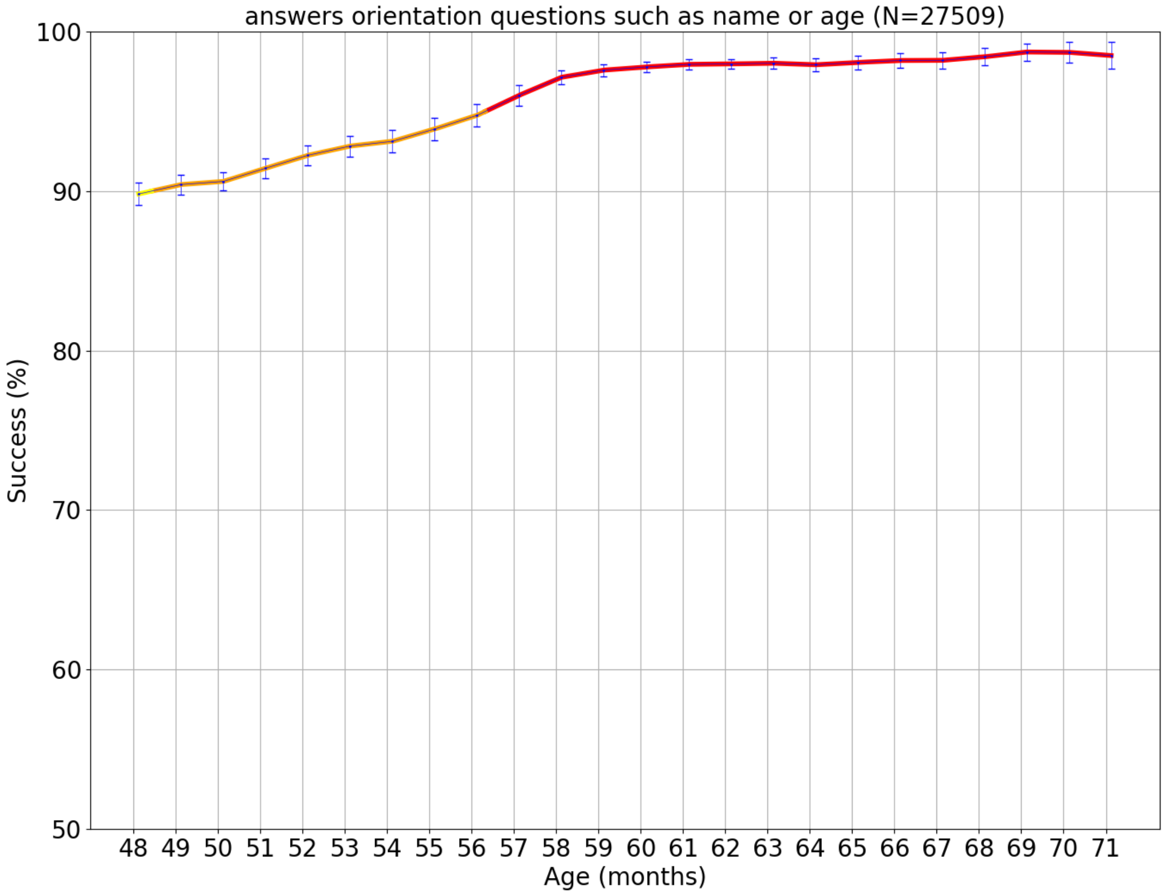

eFigure 9: The success rates over time of the different milestones evaluated between the ages 48-72 months. The achievement rates of < 75%, 75-90%, 90-95% and > 95% of children are indicated by the colors green, yellow, orange and red, respectively. The error bars are 95% confidence intervals of a binomial proportion, where larger bars indicate smaller sample size.

© 2022 Sudry T et al. *JAMA Network Open.*

eFigure 10. Comparison of Milestone Achievement Threshold by Clinical Observation and Parental Report Versus Clinical Observation Alone

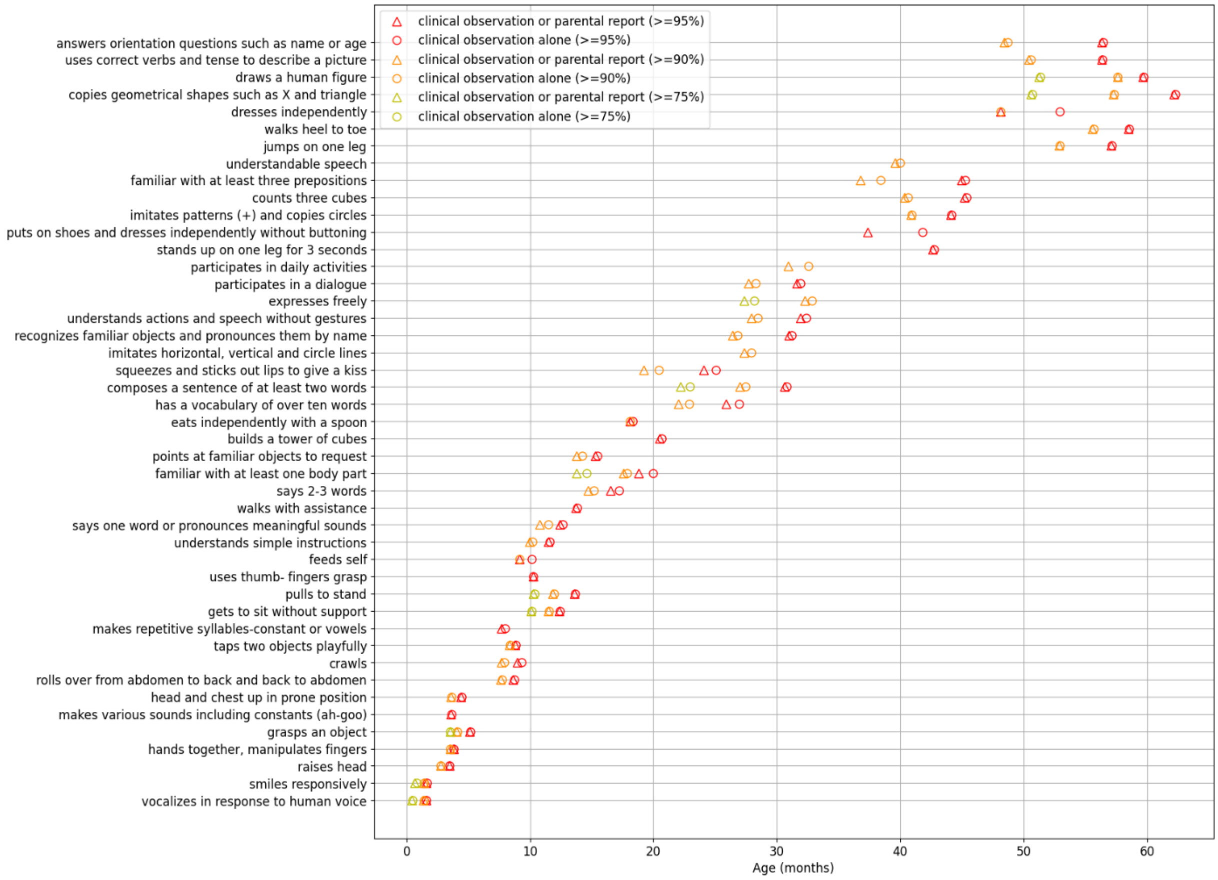

eFigure 10: comparison of milestone achievement threshold by clinical observation and parental report versus clinical observation alone. The triangle shape represents the threshold determined for the milestone achievement based on clinical observation and parental report, whereas the circle shape represents the achievement threshold based on clinical observation alone. The different colors represent the success rate of 75-90%, 90-95% and >95% of evaluated children, using yellow, orange and red color respectively.

© 2022 Sudry T et al. *JAMA Network Open.*

eTable 1. Israeli Ministry of Health: A Guide to Performing Developmental Assessments for Infants and Toddlers From Birth up to the Age of Six Years

milestone	Method of evaluation	pass
Age 6 weeks to 3 months		
	The baby lies on a flat surface, secure	
Visually follows a moving object horizontally	While lying on the back, the baby focuses his gaze on an object such as a colorful ring or a ball, the face of the parent or the examiner.<br><br>The object, the face of the examiner or the parents should be within 20 to 30 centimetres from the baby’s face.<br><br>Place the object or face in front of the baby's face and move in slow motion approximately 45 degrees to one side, back to the center, and then to the other side.<br><br>The test should not be accompanied by sounds or voices.	The baby visually follows a moving object 45 degrees horizontally both directions
Vocalizes in response to human voice	Establishing eye contact with the baby is it preliminary condition to the accomplishment of this task.<br><br>it is recommended to guide the parent to make speech sounds to the baby, while providing a recess, to allow the baby to listen and respond.	The baby makes sounds of joy or vocalize differently according to different needs.
Smiles responsively	Establishing eye contact with the baby is a preliminary condition to the accomplishment of this task.<br><br>The child's response to a stimulus sound or human face during the interaction should be examined.<br><br>To evoke a smile, the examiner can bring his face closer to the baby with a wide smile and speak softly.	The baby smiles in response to human face or human voice or stimuli.
Raises head	The baby lies on his abdomen. To see spontaneous movements, the examiner should refrain from placing his hand on the baby’s back or the pelvis.	The baby lifts his head off the surface momentarily and turn to either side.
Age 3-6 months		
Visually follows a moving object vertically	The baby follows a moving object vertically: up or down, within the middle line in a distance of 20 centimeters from his face	The baby follows a moving object vertically.

© 2022 Sudry T et al. *JAMA Network Open.*

Responds to rattling sound	The baby lies down or is in his parents' lap. A noise is produced by a rattle (a noisemaker, a knocking noise) in a distance of 50 centimeters from both sides of the baby's head and outside his vision range.<br><br>The noise production will be made in the same plane as the baby's ears and not higher.<br><br>Please note that there are no background noises during the evaluation.	The baby responds to the rattling sound in some manner, including: cessation of activity, eye blinking, head turning towards the sound origin.
Responds to human presence	When the baby is relaxed, a response to a human figure, touch or voice is examined.	The baby smiles spontaneously at a human figure, touch or human voice, or if when playing alone, stops crying when spoken to and sometimes mimics facial expressions.
makes various sounds including constants (I.e. Mm rr gg)	Various voices including constants such as ahh or goo.<br>During the entire visit attention should be paid to the production of these various voices.<br>The baby plays "vocally" by himself or when played with makes noises when he is excited or dissatisfied.	A variety of sounds are heard including consonants, such as – gg, grr, brr, mm, pp and etc.
Hands together, manipulates fingers	The action is observed while the baby lies on his back.<br><br>The disappearance of the initial congenital reflexes allows the baby to play with his hands in front of the center axis, in the midline.	The baby brings his hands to the midline, to his mouth or combines fingers.<br><br>Attention must be paid to the symmetry in hands movements to the midline while lying on the back.
grasps an object	During the examination the baby lies on his back. The task varies mildly according to the age in which the child arrives to the evaluation.	At the age 3-4 months the baby grasps an object that was placed in his palm, in each palm separately.<br><br>At the age of 4-5 months the baby reaches his hand to an object served to him at the middle line or placed on the chest and grasps with both hands.<br><br>At the age of 5-6 months the baby reaches his hand to objects that are within reachable distance, grabs them and approximates them to his body or looks at them.
Head and chest up in prone position	The baby lies on the abdomen, the examiner should refrain from intervening by placing hand on the baby's back or pelvis. The baby's movements are directed towards achieving a goal, such as a directed look at an object or figure. His curiosity in exploring the environment should also be noted.	The baby lifts his head and chest while leaning on the forearms.
Age 6-9 months		

© 2022 Sudry T et al. *JAMA Network Open.*

transfers an object from one hand to the other	The baby is lying on the back or sitting on the parent's lap, the examiner in front of him presents him with a toy in the middle line.	The baby reaches out one hand to the toy, grabs it and passes it to the other.<br><br>Watch the transfer in both directions and pay attention to the symmetry in the hand movements.
makes repetitive syllables-constant or vowels	Voice productions should be listened to throughout the visit.<br><br>During the "conversation" with the baby give time to allow him to listen and respond.	The baby makes mumbling sounds of syllables. The pronunciations are similar to the consonants and vowels of the language to which the baby is exposed, for example – mamha, dadha.
rolls over from abdomen to back and back to abdomen	please notice that the roll over is from the right side to the left and from the left side to the right.<br><br>The examiner can interest the baby with a toy placed within an angle of 45 degrees above the head.	The baby rolls over from the back to the abdomen and from the abdomen to the back.
Age 9-12 months		
taps two objects playfully	The task should be performed when the baby lies on the back or sits on the parent's lap. The examiner presents 2 toys or cubes, one for each hand.	The baby holds an object (cube) in each hand and taps between them.<br>The baby enjoys the sound and will continue to do so with pleasure.<br><br>Note symmetry in hands movements.
crawls	The child lies on his abdomen. The examiner should try to encourage the baby to move forward in crawling.<br>Observing the style of crawling and the symmetry of activation of both sides of the body alternately.	The baby is observed crawling. All or part of the crawling styles are possible.
vocalizes in a dialogue	Voice productions should be listened to throughout the visit. During the "conversation" with the baby give a break to allow him to listen and respond.	The baby initiates and produces double syllables in a dialogue such as "aa-dada", parts of words, or alternatively makes sounds of animals.
responds when addressed by name	The mother or the nurse refer to the child by name (the name in which the parents are accustomed of using) while being relaxed and in a soothing environment. The baby must be addressed once and then response should be waited for. this could be repeated.	the baby responds when addressed by name. Responses can be various including cessation of activity, referral of the head or look, vocal response
responds differently to familiar and stranger	During the visit, the child's reactions to the familiar parent and to the examiner whom the child does not know should be monitored and evaluated.	There is a different reaction to the familiar parent, opposed to a certain reluctance to the unfamiliar character - the child will check, examine, or cry as a reaction to the examiner.
feeds self	Serve the child food to hold in his hand or a bottle	The baby feeds himself hand-held food, such as a cookie or a cooked vegetable.<br>He Drinks from a bottle independently and from a cup with assistance.

© 2022 Sudry T et al. *JAMA Network Open.*

uses thumb-fingers grasp	A small pea-sized object (such as a raisin or cereal) is served to the baby within a reachable distance, under observation.<br>Each hand should be evaluated separately.<br>The child should be prevented from bringing the object to the mouth. Melted cereals should be used.	The child holds a small object in his thumb and 2 fingers.<br>Later the child is able to grasp the object at the tip of the fingers between finger and thumb in a pinching motion.<br>Symmetry should be monitored.
understands simple instructions	Understanding instructions is evaluated by looking or pointing, such as: "Where is the light?", "Where is the bottle / cup?", "Applause", "wave hello", etc. without guidance or demonstration.<br>Ask the toddler to point or look at a familiar object in the room.	The baby follows three simple instructions, one of them must refer to a human figure, such as “where is mom\dad?”.
gets to sit without support	The child lies on his stomach or back. The child sits by himself from various positions.	The baby moves from lying to sitting position. Sitting can be in a variety of poses.
Age 12-18 months		
says one word or pronounces meaningful sounds	Encourage the baby to converse while giving time to allow him to listen and respond.<br>It is important to listen to the baby during the entire visit.	The child says one word or pronounces meaningful sounds
expresses will vocally or with gestures	During the visit, the child's reactions to the familiar parent and to the examiner whom the child does not know should be monitored and evaluated.<br><br>The toddler can express desires in words, in a vote or by gesture. The toddler initiates.	The child initiates the expression of desires<br>vocally - uses words or parts of words.<br>By voting - for example on a bottle - "drink", or a door - "walk";<br>Or in a gesture - for example, raises his hands to be picked up.
makes eye contact and expresses reciprocity during joint game	The toddler plays with the examiner or parent with a ball, car, dice, doll - according to his preferences.	The child focuses his gaze and follows both the object and the parent or examiner during the game.<br>There is reciprocity - the child reacts to the other and shares the other while playing.
walks with assistance	The parent or examiner encourages walking by offering an interesting game at a suitable height and at a reasonable distance, when the child is assisted by furniture support (a stable chair, a stable stroller, etc.).	The toddler walks with support, moves between one furniture to the other, around walls and uses them for mobility, and / or with the help of an adult’s hand.
points at familiar objects to request	The toddler is asked to point to a familiar object in the room. For example: show me where is the ball.	The toddler understands and executes simple instructions with gestures, like pointing on an object upon request.

© 2022 Sudry T et al. *JAMA Network Open.*

pulls to stand	The toddler should be encouraged to pull himself to a standing position with the help of a chair or a low table, without an adult's assistance.	The toddler manages to rise from the floor with support in various manners, and sets himself up to a standing position.
says 2-3 words	Voice productions should be listened to throughout the visit.	The toddler consistently and regularly says 2-3 words including "dad" "mom" or pronounces consistent expressions understood to the environment and the family, such as: "Nana" for banana, "How" for dog.<br><br>In bilingual families, all words in each language must be referred to (for example, ball in both languages - is considered 2 words).
Age 18-24 months		
climbs upstairs with assistance	The child should be encouraged to climb stairs with a handrail that he can hold under supervision. the parent or examiner may also hold his hand in support.	The toddler goes up and down the stairs with the assistance of a helping hand or a handrail.
walks without assistance	The parent or examiner encourages walking by offering an interesting game at a suitable height and at a reasonable distance.	The toddler walks without assistance.
eats independently with a spoon	Information from the parent or primary caregiver should be provided regarding the manner of eating, the times and types of foods and textures; Toddler integration at family meals and experience of new foods.	The toddler eats independently with a spoon.
familiar with at least one body part	The toddler will be asked to show one or more body parts upon request and can show this to the parent or doll.	The toddler recognizes at least one body part.
builds a tower of cubes	While sitting at a small table or on a carpet, the parent or examiner presents cubes to the toddler and asks him to build a tower. If the toddler does not perform by request, the parent or examiner will demonstrate building a tower.	At the age of 18 months - The toddler builds a tower from 3 cubes or more.<br>At the age of 21 months - The toddler builds a tower from5 cubes or more.<br>Towards the age of two years - he builds a tower from 6 cubes or more.<br>It should be noted that the toddler uses both hands during the tower building.
has a vocabulary of over ten words	The conversation between the parent and the toddler should be listened to at all times during the visit.<br>It is recommended to show the toddler a picture book and encourage him to talk, it is possible to use the parent's assistance.	The toddler has a vocabulary of over ten words.<br>You can get a report from the parent on the number of words or expressions in which the toddler uses.
squeezes and sticks out lips to give a kiss	The parent or examiner ask the toddler to kiss the parent or a doll.	The toddler squeezes and sticks out lips to give a kiss.

© 2022 Sudry T et al. *JAMA Network Open.*

Age 2-3 years		
composes a sentence of at least two words	The conversation between the parent and the toddler should be listened to at all times during the visit.<br>It is recommended to show the toddler a picture book and encourage him to talk, it is possible to use the parent's assistance.<br>It is possible to get a report from the parent regarding sentences in which the toddler uses.	The toddler succesfully combines two words into a sentence, for example - father-come, mother-water, want-water.<br>It is possible to get a report from the parent regarding sentences in which the toddler uses.
runs well without falling	If possible, performed during the visit at the station. The examiner should pay attention to the child's organization before performing the task.<br>Multiple falls requires attention and reference.	The toddler runs freely and confidently, stepping on each foot fully, while paying attention to obstacles in his path and his ability to bypass them without dropping them.
climbs up and down the stairs without an adult's assistance	If possible, performed during the visit at the station. The child should be encouraged to go up and down the stairs with a railing that he can hold under supervision. Do not hold his hand in support.	The toddler goes up and down the stairs with the help of a railing, without the support of an adult.
recognizes familiar objects and pronounces them by name	The toddler is asked to name a variety of items or pictures that the examiner points to in the room while asking "What is it? What is it called?"	The toddler will name a number of items from the familiar environment, such as: clothing, furniture, games, fruits, animals and more.<br>Mispronunciations are acceptable.
understands actions and speech without gestures	The toddler is required to understand and perform actions asked by the examiner, literally without marking with a gesture or hint, with objects from the assessment kit. For example:<br>Show me what do we drink with, or give me the cup.	The toddler understands at least 3 actions such as: give, show, put.<br>Understands at least one preposition such as - on, within.
participates in a dialogue	It is advisable to listen during the entire visit to the conversation that develops between the mother and the toddler. It should be monitored and seen whether a dialogue develops with the adults during the evaluation, whether the child expresses feelings and desires appropriately. Does he ask situation-appropriate questions like: who? what? where?.	The toddler initiates a verbal dialogue with the examiner or parent in a suitable situation, expresses his desires, responds to his interlocutor's response and is able to share experiences from everyday life.<br>Please note: Reverberating words without meaning or intention of communication will not be considered as speech aimed at communication purposes, and requires further evaluation.
participates in daily activities	Interview with the parent regarding: dressing, eating, bathing, urine and bladder control, playing.<br><br>A question about diaper weaning should be asked with great sensitivity according to the nurse's acquaintance with the family, its customs and culture, and following the proper previous guidance.	

© 2022 Sudry T et al. *JAMA Network Open.*

imitates horizontal, vertical and circle lines	The toddler sits on a chair at a small table next to the parent. The examiner or the parent hands the child a sheet of paper and a pencil, shows him how to draw horizontal, vertical and circle lines.	The toddler imitates horizontal, vertical and circle lines.
Age 3-4 years		
expresses freely	The examiner can use individual pictures or a book (descriptions of the child's daily life), and ask open-ended questions: what's going on here? or tell me or mom a story about what is happening in the picture - to allow the toddler to answer in a complete sentence.<br>The toddler is able to describe a number of pictures or different situations performed by the nurse.	The toddler's ability to describe pictures and combine words is evaluated.<br>The child performs successfully if there are two-word combinations, one of which is a verb, such as: child traveling, drinking milk, and also combinations such as: want to eat, or two-words combinations (which are not verbs), which are connected by prepositional letters such as: on, in. For example: a doll in a stroller, a bear in bed, a child on a bicycle, a doll in a chair.
jumps from a stair	The child is asked to jump off a step, independently - without support.<br>Up to 3 attempts should be allowed.<br>The action can be demonstrated.	The toddler jumps from a step and lands on both feet, independently - without support.
puts on shoes and dresses independently without buttoning	The examiner should ask if the child dresses by himself, independently.	The child dresses himself but needs help with buttoning and tying laces. Wearing only one item of clothing and shoes is sufficient.<br><br>The child should not be expected to differentiate between a right shoe and a left shoe.
imitates patterns (+) and copies circles	The child sits on a chair at a small table next to the parent. The examiner or the parent hands the child a sheet of paper and a pencil, the child is asked to draw a + pattern and an O pattern.	The child successfully draws a + or O pattern.
Age 4-5 years		
stands up on one leg for 3 seconds	The child is asked to stand on one leg. Standing should be on one leg without support when the raised leg is free in the air. You can repeat the action up to 3 times. Initially the child will choose for himself on which leg to stand and then he should be encouraged to stand on the other leg.<br>Differences between the quality of action on one leg compared to the other leg are expected.	The child stands up on one leg for 3-4 seconds.

© 2022 Sudry T et al. *JAMA Network Open.*

counts three cubes	The examiner places cubes in front of the toddler and asks him to count them. Going forward the examiner extends his palm and asks to put 3 cubes in it. After the toddler has completed the task, waiting, and counting along with him the number of cubes he placed in the palm of the examiners hand. The toddler should associate each number with a cube.	The child counts at least three cubes.
familiar with at least three prepositions	It is advisable to place an object in a certain place so that the toddler will be able to understand and use the appropriate prepositions accordingly. For example: put a cube underneath the table. You can use pictures / book.	The child is familiar with at least 3 prepositions such as: on top, next to, underneath and more.
plays with peer group	Parental report regarding playing with peer group.	The child plays in a joint game with other children, initiates and develops imagination and imitation games. Is able to concentrate on board games. Familiar with simple rules of the game.
dresses independently	When preparing for a weight and height test check if the toddler is capable to undress and dress by himself. The ability to fasten buttons and zipper can be evaluated with the appropriate item in the development kit. Demonstration is possible if necessary.	The child is able to undress and dress himself, including button fastening.
understandable speech	During the visit at the station, listen to the developing conversation between the parent and the toddler: the toddler is able to tell a story, repeat a recitation / song, knows his name, uses male / female correctly.	The speech is clear and understandable to everyone.
Age 5-6 years		
uses correct verbs and tense to describe a picture	A book or pictures (including personal and family pictures) can be used to encourage the toddler to tell a story around the picture, or describing a simple story in sequence from pictures. In addition, you can evaluate the language skills during a free conversation with the toddler, from listening to him during the visit.	The child describes a picture using correst verbs and tenses.
answers orientation questions such as name or age	Ask the child to say his full name, age and address.	The chils says his full name, age and address.
jumps on one leg	The child is required to jump 2-3 times on one leg in a row, out of 2-3 attempts. Demonstration is possible if necessary. An attempt to jump on the other leg should be evaluated, although better control on one leg is expected.	The child is required to jump 2-3 times on one leg in a row, out of 2-3 attempts.

© 2022 Sudry T et al. *JAMA Network Open.*

walks heel to toe	The child is asked to walk in a straight-line heel to toe (thumb side heel), 4-7 steps in 2 out of 3 attempts.<br>Demonstration is possible.	The child walks in a straight-line heel to toe (thumb side heel), 4-7 steps in 2 out of 3 attempts.
draws a human figure	The child sits on a chair at a small table next to the parent. The examiner or the parent hands the child a sheet of paper and a pencil, the child is asked to draw a human figure. Do not guide the child while performing the task to notice and complete missing parts (organs) in the drawing.	The child draws a human figure with at least 6 clear and understandable organs.
copies geometrical shapes such as X and triangle	The child sits on a chair at a small table next to the parent. The examiner or the parent hands the child a sheet of paper and a pencil. The examiner or parent shows the child a drawing of a X and triangle and asks the child to copy the shapes.	The child copies the X or triangle.

eTable 1: The Israeli Ministry of Health protocol for developmental evaluation of infants and toddlers from birth up to the age of six years. The protocol describes the evaluated milestones for each age group, the method of evaluation and the definition of which behaviour qualifies as success.

© 2022 Sudry T et al. *JAMA Network Open.*

eTable 2. Population Analysis of Children Evaluated at Each Visit at the Age 0-3 Months

Cohort characteristics		Q1	Q2	Q3	Q4
n		132737	160024	145399	144766
Age, median [Q1,Q3]		44.0 [34.0,55.0]	62.0 [61.0,63.0]	65.0 [64.0,67.0]	75.0 [71.0,82.0]
Child Sex, n (%)	Female	66527 (50.1)	79040 (49.4)	72407 (49.8)	71585 (49.4)
	Male	66210 (49.9)	80984 (50.6)	72992 (50.2)	73181 (50.6)
Ethnic group, n (%)	Arab	33046 (24.9)	40838 (25.5)	32957 (22.7)	26577 (18.4)
	Druse	4393 (3.3)	3390 (2.1)	2466 (1.7)	1226 (0.8)
	Jewish	73944 (55.7)	92427 (57.8)	88360 (60.8)	94710 (65.4)
	Missing	16083 (12.1)	16409 (10.3)	16176 (11.1)	18613 (12.9)
	Other[a]	5271 (4.0)	6960 (4.3)	5440 (3.7)	3640 (2.5)
Mother birth country, n (%)	Israel	110428 (83.2)	130267 (81.4)	119226 (82.0)	120064 (82.9)
	Other[b]	12545 (9.5)	13335 (8.3)	12514 (8.6)	13209 (9.1)
	Europe	4598 (3.5)	6539 (4.1)	5758 (4.0)	5567 (3.8)
	FSU	3716 (2.8)	7183 (4.5)	5656 (3.9)	4193 (2.9)
	Ethiopia	1438 (1.1)	2689 (1.7)	2231 (1.5)	1723 (1.2)
	Missing	12 (0.0)	11 (0.0)	14 (0.0)	10 (0.0)
Employment status, n (%)	Missing	39766 (30.0)	47081 (29.4)	44608 (30.7)	46883 (32.4)
	Not Working	30955 (23.3)	32070 (20.0)	27936 (19.2)	28206 (19.5)
	Student	6559 (4.9)	7165 (4.5)	6427 (4.4)	5914 (4.1)
	Working	55457 (41.8)	73708 (46.1)	66428 (45.7)	63763 (44.0)
Mother's education level, n (%)	Academic	34492 (26.0)	55113 (34.4)	48393 (33.3)	42891 (29.6)
	Elementary	3280 (2.5)	2941 (1.8)	2784 (1.9)	3203 (2.2)
	High School	36667 (27.6)	45062 (28.2)	39165 (26.9)	37329 (25.8)
	Missing	43097 (32.5)	42824 (26.8)	41767 (28.7)	46352 (32.0)
	Tertiary Education	15201 (11.5)	14084 (8.8)	13290 (9.1)	14991 (10.4)
Mather's family status, n (%)	Married	118014 (88.9)	141320 (88.3)	128021 (88.0)	126635 (87.5)
	Missing	9248 (7.0)	9711 (6.1)	9032 (6.2)	10008 (6.9)
	Unmarried	3214 (2.4)	5408 (3.4)	5063 (3.5)	4669 (3.2)
	Other	1161 (0.9)	1741 (1.1)	1657 (1.1)	1890 (1.3)
	Divorced	1030 (0.8)	1758 (1.1)	1570 (1.1)	1470 (1.0)
	Widower	70 (0.1)	86 (0.1)	56 (0.0)	94 (0.1)
Consanguinity, n (%)	NO	122386 (92.2)	150888 (94.3)	137305 (94.4)	136999 (94.6)
	YES	10351 (7.8)	9136 (5.7)	8094 (5.6)	7767 (5.4)
Pregnancy week, median [Q1,Q3]		39.6 [39.0,40.4]	39.5 [38.6,40.2]	39.5 [38.6,40.3]	39.5 [38.6,40.3]
Birth weight, mean (SD)		3.4 (0.4)	3.3 (0.4)	3.3 (0.4)	3.3 (0.4)
Apgar score one minute, n (%)	<8	3697 (2.8)	4858 (3.0)	4414 (3.0)	4251 (2.9)
	>=8	127142 (95.8)	153353 (95.8)	139238 (95.8)	138010 (95.3)
	Missed	1898 (1.4)	1813 (1.1)	1747 (1.2)	2505 (1.7)
Apgar score five minutes, n (%)	<8	871 (0.7)	917 (0.6)	890 (0.6)	1018 (0.7)
	>=8	129140 (97.3)	156432 (97.8)	141873 (97.6)	140439 (97.0)
	Missed	2726 (2.1)	2675 (1.7)	2636 (1.8)	3309 (2.3)

[a] Circassians, Other Christians (non Arab), Unknown
[b] North America, South/Central America, Asia, Africa, Australia & Oceania

© 2022 Sudry T et al. *JAMA Network Open.*

Head circumference, mean (SD)		34.5 (1.3)	34.5 (1.3)	34.4 (1.3)	34.4 (1.3)
Type of birth, n (%)	Caesarean section	16535 (13.3)	26823 (17.6)	21510 (15.5)	20323 (14.8)
	Instrumental	6632 (5.3)	9256 (6.1)	8277 (6.0)	7056 (5.1)
	Spontaneous	100882 (81.3)	116566 (76.4)	108860 (78.5)	110029 (80.1)
Newborn position, n (%)	Breech	2578 (1.9)	4273 (2.7)	3371 (2.3)	3170 (2.2)
	Head	110138 (83.0)	135104 (84.4)	123297 (84.8)	121122 (83.7)
	Missing	18763 (14.1)	19121 (11.9)	17409 (12.0)	18994 (13.1)
	Other	1258 (0.9)	1526 (1.0)	1322 (0.9)	1480 (1.0)
Mother's age, n (%)	<=20	1608 (1.2)	1749 (1.1)	1547 (1.1)	1468 (1.0)
	<=40	121393 (91.5)	145839 (91.1)	132277 (91.0)	130772 (90.3)
	>40	8295 (6.2)	11174 (7.0)	10155 (7.0)	10893 (7.5)
	Missing	1441 (1.1)	1262 (0.8)	1420 (1.0)	1633 (1.1)

eTable 2 - A comparison of the study population characteristics at the ages 0-3 months. The evaluated population was divided into four quarters according to their visit time.

© 2022 Sudry T et al. *JAMA Network Open.*

eTable 3. Population Analysis of Children Evaluated at Each Visit at the Age 3-6 Months

Cohort characteristics		Q1	Q2	Q3	Q4
n		120063	185727	131599	134702
Age, median [Q1,Q3]		106.0 [95.0,120.0]	126.0 [124.0,127.0]	133.0 [131.0,135.0]	149.0 [143.0,160.0]
Child Sex, n (%)	Female	59643 (49.7)	92568 (49.8)	65353 (49.7)	66692 (49.5)
	Male	60420 (50.3)	93159 (50.2)	66246 (50.3)	68010 (50.5)
Ethnic group, n (%)	Arab	23485 (19.6)	49678 (26.7)	31507 (23.9)	26140 (19.4)
	Druse	2430 (2.0)	5076 (2.7)	2573 (2.0)	1127 (0.8)
	Jewish	74579 (62.1)	103219 (55.6)	77476 (58.9)	87033 (64.6)
	Missing	14655 (12.2)	19347 (10.4)	15335 (11.7)	17378 (12.9)
	Other[a]	4914 (4.1)	8407 (4.5)	4708 (3.6)	3024 (2.2)
Mother birth country, n (%)	Israel	96215 (80.1)	152362 (82.0)	109520 (83.2)	112659 (83.6)
	Other[b]	11305 (9.4)	15497 (8.3)	11388 (8.7)	12603 (9.4)
	Europe	5346 (4.5)	7318 (3.9)	4628 (3.5)	4831 (3.6)
	FSU	5388 (4.5)	7645 (4.1)	4313 (3.3)	3164 (2.3)
	Ethiopia	1800 (1.5)	2890 (1.6)	1736 (1.3)	1440 (1.1)
	Missing	9 (0.0)	15 (0.0)	14 (0.0)	5 (0.0)
Employment status, n (%)	Missing	38052 (31.7)	54178 (29.2)	40484 (30.8)	43322 (32.2)
	Not Working	24246 (20.2)	39587 (21.3)	26588 (20.2)	27526 (20.4)
	Student	4888 (4.1)	8511 (4.6)	6145 (4.7)	5883 (4.4)
	Working	52877 (44.0)	83451 (44.9)	58382 (44.4)	57971 (43.0)
Mother's education level, n (%)	Academic	36141 (30.1)	62123 (33.4)	42594 (32.4)	37229 (27.6)
	Elementary	2787 (2.3)	3609 (1.9)	2527 (1.9)	3123 (2.3)
	High School	32263 (26.9)	52838 (28.4)	35071 (26.6)	35258 (26.2)
	Missing	37597 (31.3)	50846 (27.4)	38950 (29.6)	43701 (32.4)
	Tertiary Education	11275 (9.4)	16311 (8.8)	12457 (9.5)	15391 (11.4)
Mather's family status, n (%)	Married	104663 (87.2)	164518 (88.6)	116323 (88.4)	118701 (88.1)
	Missing	8095 (6.7)	11155 (6.0)	8419 (6.4)	9522 (7.1)
	Unmarried	4275 (3.6)	6036 (3.2)	4158 (3.2)	3705 (2.8)
	Other	1519 (1.3)	1990 (1.1)	1423 (1.1)	1498 (1.1)
	Divorced	1433 (1.2)	1949 (1.0)	1212 (0.9)	1201 (0.9)
	Widower	78 (0.1)	79 (0.0)	64 (0.0)	75 (0.1)
Consanguinity, n (%)	NO	113066 (94.2)	173934 (93.7)	123555 (93.9)	126970 (94.3)
	YES	6997 (5.8)	11793 (6.3)	8044 (6.1)	7732 (5.7)
Pregnancy week, median [Q1,Q3]		39.5 [38.6,40.3]	39.5 [38.6,40.3]	39.5 [38.6,40.3]	39.6 [38.6,40.3]
Birth weight, mean (SD)		3.3 (0.4)	3.3 (0.4)	3.3 (0.4)	3.3 (0.4)
Apgar score one minute, n (%)	<8	3855 (3.2)	5654 (3.0)	3796 (2.9)	3640 (2.7)
	>=8	114201 (95.1)	177891 (95.8)	126241 (95.9)	128682 (95.5)
	Missed	2007 (1.7)	2182 (1.2)	1562 (1.2)	2380 (1.8)
	<8	908 (0.8)	1063 (0.6)	792 (0.6)	892 (0.7)
	>=8	116451 (97.0)	181379 (97.7)	128503 (97.6)	130646 (97.0)

[a] Circassians, Other Christians (non Arab), Unknown

[b] North America, South/Central America, Asia, Africa, Australia & Oceania

© 2022 Sudry T et al. *JAMA Network Open.*

Apgar score five minutes, n (%)	Missed	2704 (2.3)	3285 (1.8)	2304 (1.8)	3164 (2.3)
Head circumference, mean (SD)		34.5 (1.3)	34.5 (1.3)	34.4 (1.3)	34.4 (1.3)
Type of birth, n (%)	Caesarean section	17047 (15.2)	29833 (16.9)	19520 (15.6)	17781 (13.9)
	Instrumental	6643 (5.9)	10676 (6.0)	7054 (5.6)	6317 (4.9)
	Spontaneous	88279 (78.8)	136400 (77.1)	98890 (78.8)	104021 (81.2)
Newborn position, n (%)	Breech	2675 (2.2)	4796 (2.6)	3072 (2.3)	2756 (2.0)
	Head	97283 (81.0)	157233 (84.7)	111989 (85.1)	113273 (84.1)
	Missing	18924 (15.8)	21991 (11.8)	15310 (11.6)	17242 (12.8)
	Other	1181 (1.0)	1707 (0.9)	1228 (0.9)	1431 (1.1)
Mother's age, n (%)	<=20	1292 (1.1)	2184 (1.2)	1508 (1.1)	1485 (1.1)
	<=40	108767 (90.6)	169549 (91.3)	119794 (91.0)	121983 (90.6)
	>40	9020 (7.5)	12569 (6.8)	9016 (6.9)	9548 (7.1)
	Missing	984 (0.8)	1425 (0.8)	1281 (1.0)	1686 (1.3)

eTable 3 - A comparison of the study population characteristics at the ages 3-6 months. The evaluated population was divided into four quarters according to their visit time.

© 2022 Sudry T et al. *JAMA Network Open.*

eTable 4. Population Analysis of Children Evaluated at Each Visit at the Age 6-9 Months

Cohort characteristics		Q1	Q2	Q3	Q4
n		130823	145840	133850	131994
Age, median [Q1,Q3]		186.0 [184.0,188.0]	194.0 [192.0,196.0]	204.0 [201.0,209.0]	229.0 [220.0,241.0]
Child Sex, n (%)	Female	65583 (50.1)	72809 (49.9)	66555 (49.7)	65070 (49.3)
	Male	65240 (49.9)	73031 (50.1)	67295 (50.3)	66924 (50.7)
Ethnic group, n (%)	Arab	30556 (23.4)	38525 (26.4)	32325 (24.2)	23851 (18.1)
	Druse	3924 (3.0)	3840 (2.6)	2268 (1.7)	858 (0.7)
	Jewish	75880 (58.0)	80861 (55.4)	78706 (58.8)	87187 (66.1)
	Missing	14030 (10.7)	15986 (11.0)	15936 (11.9)	17381 (13.2)
	Other[a]	6433 (4.9)	6628 (4.5)	4615 (3.4)	2717 (2.1)
Mother birth country, n (%)	Israel	104389 (79.8)	119938 (82.2)	111621 (83.4)	110244 (83.5)
	Other[b]	11319 (8.7)	12197 (8.4)	11678 (8.7)	12635 (9.6)
	Europe	5890 (4.5)	5674 (3.9)	4768 (3.6)	4734 (3.6)
	FSU	6860 (5.2)	5835 (4.0)	4163 (3.1)	2988 (2.3)
	Ethiopia	2358 (1.8)	2183 (1.5)	1612 (1.2)	1382 (1.0)
	Missing	7 (0.0)	13 (0.0)	8 (0.0)	11 (0.0)
Employment status, n (%)	Missing	39454 (30.2)	43492 (29.8)	41479 (31.0)	43228 (32.7)
	Not Working	26881 (20.5)	31172 (21.4)	27919 (20.9)	26304 (19.9)
	Student	5572 (4.3)	6695 (4.6)	6063 (4.5)	5678 (4.3)
	Working	58916 (45.0)	64481 (44.2)	58389 (43.6)	56784 (43.0)
Mother's education level, n (%)	Academic	43500 (33.3)	48854 (33.5)	42279 (31.6)	35424 (26.8)
	Elementary	2644 (2.0)	2765 (1.9)	2758 (2.1)	3133 (2.4)
	High School	37464 (28.6)	40776 (28.0)	36313 (27.1)	33865 (25.7)
	Missing	36269 (27.7)	40898 (28.0)	39665 (29.6)	44140 (33.4)
	Tertiary Education	10946 (8.4)	12547 (8.6)	12835 (9.6)	15432 (11.7)
Mather's family status, n (%)	Married	114944 (87.9)	128914 (88.4)	118435 (88.5)	116356 (88.2)
	Missing	7776 (5.9)	8889 (6.1)	8537 (6.4)	9391 (7.1)
	Unmarried	4889 (3.7)	4794 (3.3)	4015 (3.0)	3546 (2.7)
	Other	1586 (1.2)	1621 (1.1)	1503 (1.1)	1437 (1.1)
	Divorced	1549 (1.2)	1545 (1.1)	1290 (1.0)	1192 (0.9)
	Widower	79 (0.1)	77 (0.1)	70 (0.1)	72 (0.1)
Consanguinity, n (%)	NO	123270 (94.2)	136495 (93.6)	125451 (93.7)	124354 (94.2)
	YES	7553 (5.8)	9345 (6.4)	8399 (6.3)	7640 (5.8)
Pregnancy week, median [Q1,Q3]		39.5 [38.6,40.3]	39.5 [38.6,40.2]	39.5 [38.6,40.3]	39.6 [39.0,40.3]
Birth weight, mean (SD)		3.3 (0.4)	3.3 (0.4)	3.3 (0.4)	3.3 (0.4)
Apgar score one minute, n (%)	<8	4199 (3.2)	4515 (3.1)	3895 (2.9)	3514 (2.7)
	>=8	124941 (95.5)	139604 (95.7)	128180 (95.8)	125924 (95.4)
	Missed	1683 (1.3)	1721 (1.2)	1775 (1.3)	2556 (1.9)

[a] Circassians, Other Christians (non Arab), Unknown

[b] North America, South/Central America, Asia, Africa, Australia & Oceania

© 2022 Sudry T et al. *JAMA Network Open.*

Apgar score five minutes, n (%)	<8	878 (0.7)	897 (0.6)	823 (0.6)	867 (0.7)
	>=8	127504 (97.5)	142384 (97.6)	130535 (97.5)	127785 (96.8)
	Missed	2441 (1.9)	2559 (1.8)	2492 (1.9)	3342 (2.5)
Head circumference, mean (SD)		34.5 (1.3)	34.5 (1.3)	34.4 (1.4)	34.5 (1.3)
Type of birth, n (%)	Caesarean section	20516 (16.7)	23059 (16.6)	19905 (15.6)	16783 (13.4)
	Instrumental	7608 (6.2)	8456 (6.1)	7131 (5.6)	6067 (4.8)
	Spontaneous	94467 (77.1)	107327 (77.3)	100250 (78.8)	102349 (81.7)
Newborn position, n (%)	Breech	3311 (2.5)	3659 (2.5)	3107 (2.3)	2655 (2.0)
	Head	107667 (82.3)	123332 (84.6)	113002 (84.4)	110321 (83.6)
	Missing	18758 (14.3)	17531 (12.0)	16450 (12.3)	17594 (13.3)
	Other	1087 (0.8)	1318 (0.9)	1291 (1.0)	1424 (1.1)
Mother's age, n (%)	<=20	1451 (1.1)	1698 (1.2)	1648 (1.2)	1442 (1.1)
	<=40	119264 (91.2)	133110 (91.3)	121596 (90.8)	119551 (90.6)
	>40	9340 (7.1)	9926 (6.8)	9341 (7.0)	9349 (7.1)
	Missing	768 (0.6)	1106 (0.8)	1265 (0.9)	1652 (1.3)

eTable 4 - A comparison of the study population characteristics at the ages 6-9 months. The evaluated population was divided into four quarters according to their visit time.

© 2022 Sudry T et al. *JAMA Network Open.*

eTable 5. Population Analysis of Children Evaluated at Each Visit at the Age 9-12 Months

Cohort characteristics		Q1	Q2	Q3	Q4
n		98723	113957	97676	102553
Age, median [Q1,Q3]		273.0 [206.0,275.0]	280.0 [278.0,283.0]	294.0 [290.0,300.0]	340.0 [319.0,376.0]
Child Sex, n (%)	Female	49366 (50.0)	56871 (49.9)	48508 (49.7)	50528 (49.3)
	Male	49357 (50.0)	57086 (50.1)	49168 (50.3)	52025 (50.7)
Ethnic group, n (%)	Arab	24042 (24.4)	30251 (26.5)	23601 (24.2)	24604 (24.0)
	Druse	2719 (2.8)	2954 (2.6)	2248 (2.3)	1502 (1.5)
	Jewish	56570 (57.3)	62164 (54.6)	56564 (57.9)	61245 (59.7)
	Missing	10628 (10.8)	12978 (11.4)	11345 (11.6)	12191 (11.9)
	Other[a]	4764 (4.8)	5610 (4.9)	3918 (4.0)	3011 (2.9)
Mother birth country, n (%)	Israel	80292 (81.3)	92375 (81.1)	80153 (82.1)	85507 (83.4)
	Other[b]	7943 (8.0)	9541 (8.4)	8525 (8.7)	9476 (9.2)
	FSU	4710 (4.8)	5478 (4.8)	3795 (3.9)	2902 (2.8)
	Europe	4290 (4.3)	4626 (4.1)	3780 (3.9)	3414 (3.3)
	Ethiopia	1480 (1.5)	1921 (1.7)	1419 (1.5)	1249 (1.2)
	Missing	8 (0.0)	16 (0.0)	4 (0.0)	5 (0.0)
Employment status, n (%)	Missing	29762 (30.1)	34217 (30.0)	30438 (31.2)	31697 (30.9)
	Not Working	21546 (21.8)	24604 (21.6)	20294 (20.8)	22478 (21.9)
	Student	4192 (4.2)	5032 (4.4)	4396 (4.5)	4481 (4.4)
	Working	43223 (43.8)	50104 (44.0)	42548 (43.6)	43897 (42.8)
Mother's education level, n (%)	Academic	33061 (33.5)	39583 (34.7)	33110 (33.9)	30610 (29.8)
	Elementary	2091 (2.1)	2415 (2.1)	2003 (2.1)	2643 (2.6)
	High School	29008 (29.4)	32338 (28.4)	26158 (26.8)	28849 (28.1)
	Missing	26244 (26.6)	30422 (26.7)	28035 (28.7)	30270 (29.5)
	Tertiary Education	8319 (8.4)	9199 (8.1)	8370 (8.6)	10181 (9.9)
Mather's family status, n (%)	Married	87708 (88.8)	100071 (87.8)	86156 (88.2)	90365 (88.1)
	Missing	5173 (5.2)	6743 (5.9)	5921 (6.1)	7035 (6.9)
	Unmarried	3344 (3.4)	4316 (3.8)	3278 (3.4)	3037 (3.0)
	Other	1274 (1.3)	1464 (1.3)	1177 (1.2)	1070 (1.0)
	Divorced	1164 (1.2)	1296 (1.1)	1083 (1.1)	1001 (1.0)
	Widower	60 (0.1)	67 (0.1)	61 (0.1)	45 (0.0)
Consanguinity, n (%)	NO	92633 (93.8)	106493 (93.5)	91700 (93.9)	95300 (92.9)
	YES	6090 (6.2)	7464 (6.5)	5976 (6.1)	7253 (7.1)
Pregnancy week, median [Q1,Q3]		39.5 [39.0,40.2]	39.5 [38.6,40.3]	39.5 [38.6,40.3]	39.5 [38.6,40.3]
Birth weight, mean (SD)		3.3 (0.4)	3.3 (0.4)	3.3 (0.4)	3.3 (0.4)
Apgar score one minute, n (%)	<8	3556 (3.6)	3733 (3.3)	2885 (3.0)	2674 (2.6)
	>=8	94081 (95.3)	108859 (95.5)	93430 (95.7)	97928 (95.5)
	Missed	1086 (1.1)	1365 (1.2)	1361 (1.4)	1951 (1.9)
	<8	1128 (1.1)	643 (0.6)	540 (0.6)	552 (0.5)

[a] Circassians, Other Christians (non Arab), Unknown

[b] North America, South/Central America, Asia, Africa, Australia & Oceania

© 2022 Sudry T et al. *JAMA Network Open.*

Apgar score five minutes, n (%)	>=8	96127 (97.4)	111269 (97.6)	95239 (97.5)	99508 (97.0)
	Missed	1468 (1.5)	2045 (1.8)	1897 (1.9)	2493 (2.4)
Head circumferenc e, mean (SD)		34.5 (1.4)	34.4 (1.3)	34.4 (1.3)	34.5 (1.3)
Type of birth, n (%)	Caesarean section	12184 (16.4)	19084 (17.0)	15844 (16.5)	15173 (15.0)
	Instrumental	4653 (6.2)	7149 (6.4)	5852 (6.1)	4965 (4.9)
	Spontaneous	57629 (77.4)	86307 (76.7)	74561 (77.5)	80873 (80.1)
Newborn position, n (%)	Breech	1899 (1.9)	3065 (2.7)	2509 (2.6)	2458 (2.4)
	Head	64555 (65.4)	100341 (88.1)	85329 (87.4)	89650 (87.4)
	Missing	31640 (32.0)	9525 (8.4)	8890 (9.1)	9409 (9.2)
	Other	629 (0.6)	1026 (0.9)	948 (1.0)	1036 (1.0)
Mother's age, n (%)	<=20	920 (0.9)	1498 (1.3)	1291 (1.3)	1191 (1.2)
	<=40	90138 (91.3)	103664 (91.0)	88753 (90.9)	93702 (91.4)
	>40	7253 (7.3)	8053 (7.1)	6895 (7.1)	7006 (6.8)
	Missing	412 (0.4)	742 (0.7)	737 (0.8)	654 (0.6)

eTable 5 - A comparison of the study population characteristics at the ages 1-3 months. The evaluated population was divided into four quarters according to their visit time.

© 2022 Sudry T et al. *JAMA Network Open.*

eTable 6. Population Analysis of Children Evaluated at Each Visit at the Age 12-18 Months

Cohort characteristics		Q1	Q2	Q3	Q4
n		122723	134172	126203	124693
Age, median [Q1,Q3]		367.0 [366.0,369.0]	375.0 [372.0,378.0]	388.0 [384.0,393.0]	428.0 [411.0,462.0]
Child Sex, n (%)	Female	61265 (49.9)	67219 (50.1)	62671 (49.7)	61529 (49.3)
	Male	61458 (50.1)	66953 (49.9)	63532 (50.3)	63164 (50.7)
Ethnic group, n (%)	Arab	37965 (30.9)	36511 (27.2)	26579 (21.1)	16142 (12.9)
	Druse	3262 (2.7)	4065 (3.0)	2196 (1.7)	653 (0.5)
	Jewish	62366 (50.8)	72544 (54.1)	77408 (61.3)	88349 (70.9)
	Missing	13987 (11.4)	14770 (11.0)	15273 (12.1)	16816 (13.5)
	Other[a]	5143 (4.2)	6282 (4.7)	4747 (3.8)	2733 (2.2)
Mother birth country, n (%)	Israel	100853 (82.2)	109812 (81.8)	103800 (82.2)	103608 (83.1)
	Other[b]	11428 (9.3)	11476 (8.6)	10762 (8.5)	11358 (9.1)
	Europe	4191 (3.4)	5178 (3.9)	5108 (4.0)	4869 (3.9)
	FSU	4553 (3.7)	5616 (4.2)	4712 (3.7)	3466 (2.8)
	Ethiopia	1690 (1.4)	2085 (1.6)	1808 (1.4)	1381 (1.1)
	Missing	8 (0.0)	5 (0.0)	13 (0.0)	11 (0.0)
Employment status, n (%)	Missing	35446 (28.9)	40298 (30.0)	40878 (32.4)	43098 (34.6)
	Not Working	31011 (25.3)	29779 (22.2)	23968 (19.0)	21548 (17.3)
	Student	5661 (4.6)	6014 (4.5)	5453 (4.3)	5121 (4.1)
	Working	50605 (41.2)	58081 (43.3)	55904 (44.3)	54926 (44.0)
Mother's education level, n (%)	Academic	36536 (29.8)	43889 (32.7)	41371 (32.8)	36306 (29.1)
	Elementary	3134 (2.6)	2751 (2.1)	2386 (1.9)	2461 (2.0)
	High School	36528 (29.8)	38357 (28.6)	33442 (26.5)	30750 (24.7)
	Missing	34873 (28.4)	37442 (27.9)	37909 (30.0)	41898 (33.6)
	Tertiary Education	11652 (9.5)	11733 (8.7)	11095 (8.8)	13278 (10.6)
Mather's family status, n (%)	Married	108343 (88.3)	118805 (88.5)	111598 (88.4)	109810 (88.1)
	Missing	8040 (6.6)	7970 (5.9)	7699 (6.1)	8387 (6.7)
	Unmarried	3737 (3.0)	4348 (3.2)	3976 (3.2)	3753 (3.0)
	Other	1324 (1.1)	1506 (1.1)	1514 (1.2)	1417 (1.1)
	Divorced	1207 (1.0)	1477 (1.1)	1344 (1.1)	1268 (1.0)
	Widower	72 (0.1)	66 (0.0)	72 (0.1)	58 (0.0)
Consanguinity, n (%)	NO	113113 (92.2)	125437 (93.5)	119272 (94.5)	119134 (95.5)
	YES	9610 (7.8)	8735 (6.5)	6931 (5.5)	5559 (4.5)
Pregnancy week, median [Q1,Q3]		39.5 [38.6,40.3]	39.5 [38.6,40.2]	39.5 [38.6,40.3]	39.6 [39.0,40.3]
Birth weight, mean (SD)		3.3 (0.4)	3.3 (0.4)	3.3 (0.4)	3.3 (0.4)
Apgar score one minute, n (%)	<8	3743 (3.0)	4173 (3.1)	3749 (3.0)	3315 (2.7)
	>=8	117316 (95.6)	128235 (95.6)	120708 (95.6)	118800 (95.3)
	Missed	1664 (1.4)	1764 (1.3)	1746 (1.4)	2578 (2.1)

[a] Circassians, Other Christians (non Arab), Unknown

[b] North America, South/Central America, Asia, Africa, Australia & Oceania

© 2022 Sudry T et al. *JAMA Network Open.*

Apgar score five minutes, n (%)	<8	790 (0.6)	852 (0.6)	796 (0.6)	866 (0.7)
	>=8	119589 (97.4)	130843 (97.5)	122953 (97.4)	120586 (96.7)
	Missed	2344 (1.9)	2477 (1.8)	2454 (1.9)	3241 (2.6)
Head circumference, mean (SD)		34.5 (1.3)	34.4 (1.3)	34.5 (1.3)	34.5 (1.3)
Type of birth, n (%)	Caesarean section	17792 (15.3)	21076 (16.6)	19057 (16.1)	16024 (13.7)
	Instrumental	6793 (5.8)	7638 (6.0)	6871 (5.8)	5729 (4.9)
	Spontaneous	91903 (78.9)	97966 (77.3)	92789 (78.2)	95216 (81.4)
Newborn position, n (%)	Breech	2812 (2.3)	3409 (2.5)	3015 (2.4)	2579 (2.1)
	Head	103739 (84.5)	111968 (83.5)	104594 (82.9)	102113 (81.9)
	Missing	15012 (12.2)	17592 (13.1)	17527 (13.9)	18771 (15.1)
	Other	1160 (0.9)	1203 (0.9)	1067 (0.8)	1230 (1.0)
Mother's age, n (%)	<=20	1673 (1.4)	1630 (1.2)	1491 (1.2)	1331 (1.1)
	<=40	111104 (90.5)	122051 (91.0)	115237 (91.3)	113611 (91.1)
	>40	8891 (7.2)	9446 (7.0)	8506 (6.7)	8742 (7.0)
	Missing	1055 (0.9)	1045 (0.8)	969 (0.8)	1009 (0.8)

eTable 6 - A comparison of the study population characteristics at the ages 12-18 months. The evaluated population was divided into four quarters according to their visit time.

© 2022 Sudry T et al. *JAMA Network Open.*

eTable 7. Population Analysis of Children Evaluated at Each Visit at the Age 18-24 Months

Cohort characteristics		Q1	Q2	Q3	Q4
n		96267	123169	108051	108967
Age, median [Q1,Q3]		548.0 [445.0,549.0]	554.0 [552.0,557.0]	571.0 [565.0,578.0]	623.0 [601.0,664.0]
Child Sex, n (%)	Female	48291 (50.2)	61463 (49.9)	53758 (49.8)	53755 (49.3)
	Male	47976 (49.8)	61706 (50.1)	54293 (50.2)	55212 (50.7)
Ethnic group, n (%)	Arab	26510 (27.5)	34034 (27.6)	25149 (23.3)	17408 (16.0)
	Druse	2785 (2.9)	3445 (2.8)	2381 (2.2)	622 (0.6)
	Jewish	51885 (53.9)	65830 (53.4)	63417 (58.7)	73827 (67.8)
	Missing	10447 (10.9)	14503 (11.8)	12939 (12.0)	14424 (13.2)
	Other[a]	4640 (4.8)	5357 (4.3)	4165 (3.9)	2686 (2.5)
Mother birth country, n (%)	Israel	78543 (81.6)	101784 (82.6)	89610 (82.9)	90228 (82.8)
	Other[b]	8248 (8.6)	10498 (8.5)	9202 (8.5)	9818 (9.0)
	Europe	3473 (3.6)	4575 (3.7)	3966 (3.7)	4310 (4.0)
	FSU	4373 (4.5)	4576 (3.7)	3870 (3.6)	3320 (3.0)
	Ethiopia	1623 (1.7)	1726 (1.4)	1397 (1.3)	1281 (1.2)
	Missing	7 (0.0)	10 (0.0)	6 (0.0)	10 (0.0)
Employment status, n (%)	Missing	28171 (29.3)	38280 (31.1)	34472 (31.9)	37167 (34.1)
	Not Working	22966 (23.9)	27614 (22.4)	21924 (20.3)	20182 (18.5)
	Student	4448 (4.6)	5352 (4.3)	4783 (4.4)	4467 (4.1)
	Working	40682 (42.3)	51923 (42.2)	46872 (43.4)	47151 (43.3)
Mother's education level, n (%)	Academic	30232 (31.4)	40208 (32.6)	35270 (32.6)	31739 (29.1)
	Elementary	2148 (2.2)	2626 (2.1)	2101 (1.9)	2360 (2.2)
	High School	28919 (30.0)	34422 (27.9)	29821 (27.6)	27840 (25.5)
	Missing	26142 (27.2)	35285 (28.6)	31392 (29.1)	35844 (32.9)
	Tertiary Education	8826 (9.2)	10628 (8.6)	9467 (8.8)	11184 (10.3)
Mather's family status, n (%)	Married	85512 (88.8)	109312 (88.7)	95976 (88.8)	96176 (88.3)
	Missing	5480 (5.7)	7279 (5.9)	6374 (5.9)	7084 (6.5)
	Unmarried	3114 (3.2)	3873 (3.1)	3251 (3.0)	3190 (2.9)
	Other	1097 (1.1)	1358 (1.1)	1207 (1.1)	1278 (1.2)
	Divorced	1018 (1.1)	1269 (1.0)	1185 (1.1)	1184 (1.1)
	Widower	46 (0.0)	78 (0.1)	58 (0.1)	55 (0.1)
Consanguinity, n (%)	NO	89743 (93.2)	114903 (93.3)	101674 (94.1)	103262 (94.8)
	YES	6524 (6.8)	8266 (6.7)	6377 (5.9)	5705 (5.2)
Pregnancy week, median [Q1,Q3]		39.5 [39.0,40.3]	39.5 [38.6,40.2]	39.5 [38.6,40.3]	39.5 [38.6,40.3]
Birth weight, mean (SD)		3.3 (0.4)	3.3 (0.4)	3.3 (0.4)	3.3 (0.4)
Apgar score one minute, n (%)	<8	2959 (3.1)	3764 (3.1)	3222 (3.0)	3097 (2.8)
	>=8	91994 (95.6)	117900 (95.7)	103229 (95.5)	103745 (95.2)
	Missed	1314 (1.4)	1505 (1.2)	1600 (1.5)	2125 (2.0)

[a] Circassians, Other Christians (non Arab), Unknown

[b] North America, South/Central America, Asia, Africa, Australia & Oceania

© 2022 Sudry T et al. *JAMA Network Open.*

Apgar score five minutes, n (%)	<8	615 (0.6)	795 (0.6)	704 (0.7)	796 (0.7)
	>=8	93840 (97.5)	120219 (97.6)	105179 (97.3)	105486 (96.8)
	Missed	1812 (1.9)	2155 (1.7)	2168 (2.0)	2685 (2.5)
Head circumference, mean (SD)		34.4 (1.3)	34.4 (1.3)	34.5 (1.3)	34.5 (1.4)
Type of birth, n (%)	Caesarean section	14028 (15.4)	19181 (16.5)	16209 (16.0)	14374 (14.3)
	Instrumental	5425 (6.0)	7119 (6.1)	5797 (5.7)	4958 (4.9)
	Spontaneous	71538 (78.6)	89834 (77.4)	79085 (78.2)	81463 (80.8)
Newborn position, n (%)	Breech	2201 (2.3)	3185 (2.6)	2561 (2.4)	2280 (2.1)
	Head	80227 (83.3)	102515 (83.2)	88968 (82.3)	88325 (81.1)
	Missing	13004 (13.5)	16406 (13.3)	15558 (14.4)	17299 (15.9)
	Other	835 (0.9)	1063 (0.9)	964 (0.9)	1063 (1.0)
Mother's age, n (%)	<=20	1346 (1.4)	1738 (1.4)	1372 (1.3)	1308 (1.2)
	<=40	87765 (91.2)	112264 (91.1)	98901 (91.5)	99427 (91.2)
	>40	6607 (6.9)	8327 (6.8)	7127 (6.6)	7511 (6.9)
	Missing	549 (0.6)	840 (0.7)	651 (0.6)	721 (0.7)

eTable 7 - A comparison of the study population characteristics at the ages 18-24 months. The evaluated population was divided into four quarters according to their visit time.

© 2022 Sudry T et al. *JAMA Network Open.*

eTable 8. Population Analysis of Children Evaluated at Each Visit at the Age 2-3 Years (24-36 Months)

Cohort characteristics		Q1	Q2	Q3	Q4
n		89856	94627	90871	91649
Age, median [Q1,Q3]		743.0 [737.0,750.0]	778.0 [768.0,790.0]	823.0 [811.0,832.0]	898.0 [867.0,956.0]
Child Sex, n (%)	Female	44683 (49.7)	47110 (49.8)	45214 (49.8)	45428 (49.6)
	Male	45173 (50.3)	47517 (50.2)	45657 (50.2)	46221 (50.4)
Ethnic group, n (%)	Arab	27213 (30.3)	26276 (27.8)	21088 (23.2)	14163 (15.5)
	Druse	1268 (1.4)	2671 (2.8)	3218 (3.5)	814 (0.9)
	Jewish	45721 (50.9)	50224 (53.1)	52912 (58.2)	62871 (68.6)
	Missing	11737 (13.1)	11374 (12.0)	9981 (11.0)	11225 (12.2)
	Other[a]	3917 (4.4)	4082 (4.3)	3672 (4.0)	2576 (2.8)
Mother birth country, n (%)	Israel	73272 (81.5)	79157 (83.7)	76150 (83.8)	76052 (83.0)
	Other[b]	8407 (9.4)	7577 (8.0)	6925 (7.6)	7341 (8.0)
	Europe	3387 (3.8)	3427 (3.6)	3272 (3.6)	3860 (4.2)
	FSU	3543 (3.9)	3303 (3.5)	3264 (3.6)	3131 (3.4)
	Ethiopia	1239 (1.4)	1158 (1.2)	1252 (1.4)	1256 (1.4)
	Missing	8 (0.0)	5 (0.0)	8 (0.0)	9 (0.0)
Employment status, n (%)	Missing	29310 (32.6)	29202 (30.9)	28806 (31.7)	31430 (34.3)
	Not Working	21715 (24.2)	21939 (23.2)	18670 (20.5)	16500 (18.0)
	Student	3918 (4.4)	4138 (4.4)	4109 (4.5)	3783 (4.1)
	Working	34913 (38.9)	39348 (41.6)	39286 (43.2)	39936 (43.6)
Mother's education level, n (%)	Academic	26379 (29.4)	30428 (32.2)	31185 (34.3)	27969 (30.5)
	Elementary	2157 (2.4)	1980 (2.1)	1721 (1.9)	1926 (2.1)
	High School	24651 (27.4)	26583 (28.1)	26609 (29.3)	24349 (26.6)
	Missing	29142 (32.4)	27539 (29.1)	23617 (26.0)	28185 (30.8)
	Tertiary Education	7527 (8.4)	8097 (8.6)	7739 (8.5)	9220 (10.1)
Mather's family status, n (%)	Married	78758 (87.6)	84458 (89.3)	81765 (90.0)	81848 (89.3)
	Missing	6044 (6.7)	5291 (5.6)	4531 (5.0)	5048 (5.5)
	Unmarried	2907 (3.2)	2811 (3.0)	2592 (2.9)	2621 (2.9)
	Other	1129 (1.3)	1032 (1.1)	942 (1.0)	1009 (1.1)
	Divorced	967 (1.1)	981 (1.0)	993 (1.1)	1064 (1.2)
	Widower	51 (0.1)	54 (0.1)	48 (0.1)	59 (0.1)
Consanguinity, n (%)	NO	83212 (92.6)	88158 (93.2)	85472 (94.1)	87398 (95.4)
	YES	6644 (7.4)	6469 (6.8)	5399 (5.9)	4251 (4.6)
Pregnancy week, median [Q1,Q3]		39.5 [38.6,40.3]	39.5 [38.6,40.3]	39.5 [38.6,40.2]	39.5 [38.6,40.3]
Birth weight, mean (SD)		3.3 (0.4)	3.3 (0.4)	3.3 (0.4)	3.3 (0.4)
Apgar score one minute, n (%)	<8	3069 (3.4)	2934 (3.1)	2600 (2.9)	2467 (2.7)
	>=8	85547 (95.2)	90406 (95.5)	87105 (95.9)	87642 (95.6)
	Missed	1240 (1.4)	1287 (1.4)	1166 (1.3)	1540 (1.7)

[a] Circassians, Other Christians (non Arab), Unknown

[b] North America, South/Central America, Asia, Africa, Australia & Oceania

© 2022 Sudry T et al. *JAMA Network Open.*

Apgar score five minutes, n (%)	<8	654 (0.7)	669 (0.7)	636 (0.7)	657 (0.7)
	>=8	87498 (97.4)	92180 (97.4)	88606 (97.5)	88996 (97.1)
	Missed	1704 (1.9)	1778 (1.9)	1629 (1.8)	1996 (2.2)
Head circumference, mean (SD)		34.5 (1.3)	34.5 (1.3)	34.5 (1.4)	34.5 (1.3)
Type of birth, n (%)	Caesarean section	13532 (15.9)	14220 (16.1)	13461 (16.1)	12104 (14.5)
	Instrumental	4963 (5.8)	5121 (5.8)	4857 (5.8)	4298 (5.2)
	Spontaneous	66600 (78.3)	68775 (78.1)	65327 (78.1)	66865 (80.3)
Newborn position, n (%)	Breech	2254 (2.5)	2298 (2.4)	2190 (2.4)	1913 (2.1)
	Head	74940 (83.4)	77595 (82.0)	73155 (80.5)	72061 (78.6)
	Missing	11800 (13.1)	13927 (14.7)	14854 (16.3)	16842 (18.4)
	Other	862 (1.0)	807 (0.9)	672 (0.7)	833 (0.9)
Mother's age, n (%)	<=20	1751 (1.9)	1464 (1.5)	1126 (1.2)	1219 (1.3)
	<=40	81734 (91.0)	86987 (91.9)	83774 (92.2)	84321 (92.0)
	>40	5768 (6.4)	5774 (6.1)	5668 (6.2)	5812 (6.3)
	Missing	603 (0.7)	402 (0.4)	303 (0.3)	297 (0.3)

eTable 8 - A comparison of the study population characteristics at the ages 2-3 years (24-36 months). The evaluated population was divided into four quarters according to their visit time.

© 2022 Sudry T et al. *JAMA Network Open.*

eTable 9. Population Analysis of Children Evaluated at Each Visit at the Age 3-4 Years (36-48 Months)

Cohort characteristics		Q1	Q2	Q3	Q4
n		30016	31379	30443	30399
Age, median [Q1,Q3]		1105.0 [1099.0,1114.0]	1150.0 [1136.0,1162.0]	1207.0 [1190.0,1226.0]	1325.0 [1283.0,1392.0]
Child Sex, n (%)	Female	15233 (50.7)	15962 (50.9)	15039 (49.4)	15110 (49.7)
	Male	14783 (49.3)	15417 (49.1)	15404 (50.6)	15289 (50.3)
Ethnic group, n (%)	Arab	10340 (34.4)	9649 (30.7)	7800 (25.6)	7160 (23.6)
	Druse	765 (2.5)	1636 (5.2)	1333 (4.4)	585 (1.9)
	Jewish	14111 (47.0)	15389 (49.0)	16994 (55.8)	18360 (60.4)
	Missing	3073 (10.2)	2883 (9.2)	2765 (9.1)	3115 (10.2)
	Other[a]	1727 (5.8)	1822 (5.8)	1551 (5.1)	1179 (3.9)
Mother birth country, n (%)	Israel	25334 (84.4)	26537 (84.6)	25568 (84.0)	25606 (84.2)
	Other[b]	1883 (6.3)	2014 (6.4)	1944 (6.4)	2079 (6.8)
	Europe	1100 (3.7)	1182 (3.8)	1180 (3.9)	1294 (4.3)
	FSU	1255 (4.2)	1185 (3.8)	1226 (4.0)	979 (3.2)
	Ethiopia	442 (1.5)	458 (1.5)	523 (1.7)	438 (1.4)
	Missing	2 (0.0)	3 (0.0)	2 (0.0)	3 (0.0)
Employment status, n (%)	Missing	9637 (32.1)	9420 (30.0)	9712 (31.9)	9974 (32.8)
	Not Working	7576 (25.2)	7795 (24.8)	6636 (21.8)	6551 (21.6)
	Student	1262 (4.2)	1463 (4.7)	1385 (4.5)	1313 (4.3)
	Working	11541 (38.4)	12701 (40.5)	12710 (41.8)	12561 (41.3)
Mother's education level, n (%)	Academic	10639 (35.4)	11155 (35.5)	10847 (35.6)	10002 (32.9)
	Elementary	534 (1.8)	608 (1.9)	605 (2.0)	746 (2.5)
	High School	8842 (29.5)	10022 (31.9)	9347 (30.7)	8816 (29.0)
	Missing	7922 (26.4)	7220 (23.0)	7322 (24.1)	8275 (27.2)
	Tertiary Education	2079 (6.9)	2374 (7.6)	2322 (7.6)	2560 (8.4)
Mather's family status, n (%)	Married	27034 (90.1)	28362 (90.4)	27686 (90.9)	27423 (90.2)
	Missing	1298 (4.3)	1374 (4.4)	1200 (3.9)	1414 (4.7)
	Unmarried	933 (3.1)	894 (2.8)	848 (2.8)	819 (2.7)
	Divorced	390 (1.3)	381 (1.2)	375 (1.2)	359 (1.2)
	Other	342 (1.1)	348 (1.1)	314 (1.0)	366 (1.2)
	Widower	19 (0.1)	20 (0.1)	20 (0.1)	18 (0.1)
Consanguinity, n (%)	NO	28057 (93.5)	29176 (93.0)	28497 (93.6)	28612 (94.1)
	YES	1959 (6.5)	2203 (7.0)	1946 (6.4)	1787 (5.9)
Pregnancy week, median [Q1,Q3]		39.5 [38.6,40.2]	39.5 [38.6,40.2]	39.5 [38.6,40.2]	39.5 [39.0,40.2]
Birth weight, mean (SD)		3.3 (0.4)	3.3 (0.4)	3.3 (0.4)	3.3 (0.4)
	<8	953 (3.2)	900 (2.9)	893 (2.9)	897 (3.0)
	>=8	28717 (95.7)	30115 (96.0)	29139 (95.7)	29047 (95.6)

[a] Circassians, Other Christians (non Arab), Unknown

[b] North America, South/Central America, Asia, Africa, Australia & Oceania

© 2022 Sudry T et al. *JAMA Network Open.*

Apgar score one minute, n (%)	Missed	346 (1.2)	364 (1.2)	411 (1.4)	455 (1.5)
Apgar score five minutes, n (%)	<8	209 (0.7)	192 (0.6)	224 (0.7)	247 (0.8)
	>=8	29322 (97.7)	30682 (97.8)	29677 (97.5)	29579 (97.3)
	Missed	485 (1.6)	505 (1.6)	542 (1.8)	573 (1.9)
Head circumference, mean (SD)		34.5 (1.3)	34.5 (1.3)	34.5 (1.4)	34.5 (1.4)
Type of birth, n (%)	Caesarean section	4657 (17.0)	4869 (17.2)	4469 (16.4)	4157 (15.8)
	Instrumental	1779 (6.5)	1776 (6.3)	1669 (6.1)	1558 (5.9)
	Spontaneous	20936 (76.5)	21698 (76.6)	21078 (77.4)	20547 (78.2)
Newborn position, n (%)	Breech	750 (2.5)	744 (2.4)	758 (2.5)	657 (2.2)
	Head	23750 (79.1)	24519 (78.1)	23643 (77.7)	22428 (73.8)
	Missing	5302 (17.7)	5890 (18.8)	5818 (19.1)	7078 (23.3)
	Other	214 (0.7)	226 (0.7)	224 (0.7)	236 (0.8)
Mother's age, n (%)	<=20	594 (2.0)	509 (1.6)	432 (1.4)	384 (1.3)
	<=40	27853 (92.8)	29409 (93.7)	28505 (93.6)	28420 (93.5)
	>40	1512 (5.0)	1412 (4.5)	1474 (4.8)	1536 (5.1)
	Missing	57 (0.2)	49 (0.2)	32 (0.1)	59 (0.2)

eTable 9 - A comparison of the study population characteristics at the ages 3-4 years (36-48 months). The evaluated population was divided into four quarters according to their visit time.

© 2022 Sudry T et al. *JAMA Network Open.*

eTable 10. Population Analysis of Children Evaluated at Each Visit at the Age 4-6 Years (48-72 Months)

Cohort characteristics		Q1	Q2	Q3	Q4
n		6982	7054	7030	6988
Age, median [Q1,Q3]		1487.0 [1470.0,1507.0]	1605.0 [1567.0,1650.0]	1832.0 [1785.0,1855.0]	1975.0 [1924.0,2054.0]
Child Sex, n (%)	Female	3386 (48.5)	3460 (49.1)	3569 (50.8)	3515 (50.3)
	Male	3596 (51.5)	3594 (50.9)	3461 (49.2)	3473 (49.7)
Ethnic group, n (%)	Arab	1893 (27.1)	1578 (22.4)	1918 (27.3)	1693 (24.2)
	Druse	175 (2.5)	165 (2.3)	150 (2.1)	48 (0.7)
	Jewish	3846 (55.1)	4297 (60.9)	4004 (57.0)	4213 (60.3)
	Missing	730 (10.5)	771 (10.9)	680 (9.7)	740 (10.6)
	Other[a]	338 (4.8)	243 (3.4)	278 (4.0)	294 (4.2)
Mother birth country, n (%)	Israel	5838 (83.6)	5920 (83.9)	5843 (83.1)	5663 (81.0)
	Other[b]	491 (7.0)	509 (7.2)	468 (6.7)	515 (7.4)
	Europe	265 (3.8)	284 (4.0)	306 (4.4)	358 (5.1)
	FSU	293 (4.2)	232 (3.3)	274 (3.9)	302 (4.3)
	Ethiopia	95 (1.4)	109 (1.5)	138 (2.0)	150 (2.1)
	Missing			1 (0.0)	
Employment status, n (%)	Missing	2220 (31.8)	2349 (33.3)	2326 (33.1)	2427 (34.7)
	Not Working	1681 (24.1)	1572 (22.3)	1578 (22.4)	1434 (20.5)
	Student	277 (4.0)	329 (4.7)	268 (3.8)	272 (3.9)
	Working	2804 (40.2)	2804 (39.8)	2858 (40.7)	2855 (40.9)
Mother's education level, n (%)	Academic	2133 (30.5)	2118 (30.0)	2402 (34.2)	2430 (34.8)
	Elementary	167 (2.4)	165 (2.3)	143 (2.0)	213 (3.0)
	High School	2217 (31.8)	2083 (29.5)	2068 (29.4)	1904 (27.2)
	Missing	1875 (26.9)	2013 (28.5)	1935 (27.5)	2012 (28.8)
	Tertiary Education	590 (8.5)	675 (9.6)	482 (6.9)	429 (6.1)
Mather's family status, n (%)	Married	6256 (89.6)	6365 (90.2)	6363 (90.5)	6191 (88.6)
	Missing	308 (4.4)	316 (4.5)	295 (4.2)	372 (5.3)
	Unmarried	236 (3.4)	199 (2.8)	199 (2.8)	221 (3.2)
	Divorced	108 (1.5)	81 (1.1)	92 (1.3)	110 (1.6)
	Other	70 (1.0)	85 (1.2)	77 (1.1)	87 (1.2)
	Widower	4 (0.1)	8 (0.1)	4 (0.1)	7 (0.1)
Consanguinity, n (%)	NO	6539 (93.7)	6606 (93.6)	6654 (94.7)	6640 (95.0)
	YES	443 (6.3)	448 (6.4)	376 (5.3)	348 (5.0)
Pregnancy week, median [Q1,Q3]		39.5 [38.6,40.2]	39.6 [39.0,40.2]	39.5 [39.0,40.1]	39.3 [38.6,40.0]
Birth weight, mean (SD)		3.3 (0.4)	3.3 (0.4)	3.3 (0.4)	3.3 (0.4)
	<8	209 (3.0)	212 (3.0)	274 (3.9)	278 (4.0)
	>=8	6666 (95.5)	6743 (95.6)	6664 (94.8)	6641 (95.0)

[a] Circassians, Other Christians (non Arab), Unknown

[b] North America, South/Central America, Asia, Africa, Australia & Oceania

© 2022 Sudry T et al. *JAMA Network Open.*

Apgar score one minute, n (%)	Missed	107 (1.5)	99 (1.4)	92 (1.3)	69 (1.0)
Apgar score five minutes, n (%)	<8	59 (0.8)	52 (0.7)	78 (1.1)	118 (1.7)
	>=8	6795 (97.3)	6866 (97.3)	6835 (97.2)	6784 (97.1)
	Missed	128 (1.8)	136 (1.9)	117 (1.7)	86 (1.2)
Head circumference, mean (SD)		34.5 (1.3)	34.5 (1.4)	34.5 (1.4)	34.5 (1.5)
Type of birth, n (%)	Caesarean section	1069 (18.1)	863 (14.8)	872 (16.3)	651 (15.7)
	Instrumental	330 (5.6)	328 (5.6)	323 (6.0)	268 (6.4)
	Spontaneous	4501 (76.3)	4659 (79.6)	4150 (77.6)	3239 (77.9)
Newborn position, n (%)	Breech	152 (2.2)	136 (1.9)	141 (2.0)	114 (1.6)
	Head	4962 (71.1)	4904 (69.5)	4374 (62.2)	3286 (47.0)
	Missing	1816 (26.0)	1979 (28.1)	2484 (35.3)	3557 (50.9)
	Other	52 (0.7)	35 (0.5)	31 (0.4)	31 (0.4)
Mother's age, n (%)	<=20	78 (1.1)	60 (0.9)	50 (0.7)	44 (0.6)
	<=40	6509 (93.2)	6576 (93.2)	6547 (93.1)	6477 (92.7)
	>40	384 (5.5)	405 (5.7)	424 (6.0)	454 (6.5)
	Missing	11 (0.2)	13 (0.2)	9 (0.1)	13 (0.2)

eTable 10 - A comparison of the study population characteristics at the ages 4-6 years (48-72 months). The evaluated population was divided into four quarters according to their visit time.

© 2022 Sudry T et al. *JAMA Network Open.*

eTable 11. Comparison of the Characteristics of Evaluated Children Between the Ages 0-3 Years and 3-6 Years

Cohort characteristics		Age 0-3 years	Age 3-6 years
n		643940	134378
Age, median [Q1,Q3]		64.0 [61.0,72.0]	1188.0 [1129.0,1289.0]
Child Sex, n (%)	Female	319552 (49.6)	67472 (50.2)
	Male	324388 (50.4)	66906 (49.8)
Ethnic group, n (%)	Arab	140525 (21.8)	37055 (27.6)
	Druse	11796 (1.8)	4494 (3.3)
	Jewish	392621 (61.0)	72830 (54.2)
	Missing	76328 (11.9)	13337 (9.9)
	Other[a]	22670 (3.5)	6662 (5.0)
Mother birth country, n (%)	Israel	528851 (82.1)	113157 (84.2)
	Other[b]	58979 (9.2)	8883 (6.6)
	Europe	25176 (3.9)	5267 (3.9)
	FSU	22240 (3.5)	5016 (3.7)
	Ethiopia	8644 (1.3)	2045 (1.5)
	Missing	50 (0.0)	10 (0.0)
Employment status, n (%)	Missing	199537 (31.0)	43089 (32.1)
	Not Working	131698 (20.5)	30890 (23.0)
	Student	28513 (4.4)	5924 (4.4)
	Working	284192 (44.1)	54475 (40.5)
Mother's education level, n (%)	Academic	195796 (30.4)	46303 (34.5)
	Elementary	13441 (2.1)	2775 (2.1)
	High School	171408 (26.6)	40263 (30.0)
	Missing	199011 (30.9)	34600 (25.7)
	Tertiary Education	64284 (10.0)	10437 (7.8)
Mather's family status, n (%)	Married	566735 (88.0)	121342 (90.3)
	Missing	43377 (6.7)	5914 (4.4)
	Unmarried	20004 (3.1)	3874 (2.9)
	Other	7158 (1.1)	1504 (1.1)
	Divorced	6343 (1.0)	1657 (1.2)
	Widower	323 (0.1)	87 (0.1)
Consanguinity, n (%)	NO	606211 (94.1)	125885 (93.7)
	YES	37729 (5.9)	8493 (6.3)
Pregnancy week, median [Q1,Q3]		39.5 [38.6,40.3]	39.5 [38.6,40.2]
Birth weight, mean (SD)		3.3 (0.4)	3.3 (0.4)
Apgar score one minute, n (%)	<8	18860 (2.9)	4073 (3.0)
	>=8	613734 (95.3)	128549 (95.7)
	Missed	11346 (1.8)	1756 (1.3)
Apgar score five minutes, n (%)	<8	4112 (0.6)	1021 (0.8)
	>=8	624673 (97.0)	131023 (97.5)
	Missed	15155 (2.4)	2334 (1.7)

[a] Circassians, Other Christians (non Arab), Unknown

[b] North America, South/Central America, Asia, Africa, Australia & Oceania

© 2022 Sudry T et al. *JAMA Network Open.*

Head circumference, mean (SD)		34.5 (1.3)	34.5 (1.4)
Type of birth, n (%)	Caesarean section	92538 (15.1)	19528 (16.5)
	Instrumental	34008 (5.6)	7278 (6.1)
	Spontaneous	485450 (79.3)	91637 (77.4)
Newborn position, n (%)	Breech	14532 (2.3)	3137 (2.3)
	Head	540290 (83.9)	101975 (75.9)
	Missing	82828 (12.9)	28304 (21.1)
	Other	6290 (1.0)	962 (0.7)
Mother's age, n (%)	<=20	7100 (1.1)	2014 (1.5)
	<=40	584820 (90.8)	125432 (93.3)
	>40	45092 (7.0)	6712 (5.0)
	Missing	6928 (1.1)	220 (0.2)

eTable 11 - A comparison of the study population characteristics divided to two groups - from birth to 3 years and 3-6 years.

© 2022 Sudry T et al. *JAMA Network Open.*
